# Vepafestinib is a pharmacologically advanced RET-selective inhibitor with high CNS penetration and inhibitory activity against RET solvent front mutations

**DOI:** 10.1038/s43018-023-00630-y

**Published:** 2023-09-21

**Authors:** Isao Miyazaki, Igor Odintsov, Keiji Ishida, Allan J. W. Lui, Masanori Kato, Tatsuya Suzuki, Tom Zhang, Kentaro Wakayama, Renate I. Kurth, Ryan Cheng, Hidenori Fujita, Lukas Delasos, Morana Vojnic, Inna Khodos, Yukari Yamada, Kota Ishizawa, Marissa S. Mattar, Kaoru Funabashi, Qing Chang, Shuichi Ohkubo, Wakako Yano, Ryuichiro Terada, Claudio Giuliano, Yue Christine Lu, Annalisa Bonifacio, Siddharth Kunte, Monika A. Davare, Emily H. Cheng, Elisa de Stanchina, Emanuela Lovati, Yoshikazu Iwasawa, Marc Ladanyi, Romel Somwar

**Affiliations:** 1 Taiho Pharmaceutical Co. Ltd., Tsukuba, Japan; 2 Department of Pathology and Laboratory Medicine, Memorial Sloan Kettering Cancer Center, New York, NY USA; 3 Human Oncology and Pathogenesis Program, Memorial Sloan Kettering Cancer Center, New York, NY USA; 4 Antitumor Assessment Core Facility, Molecular Pharmacology Program, Memorial Sloan Kettering Cancer Center, New York, NY USA; 5 Helsinn Healthcare SA, Lugano, Switzerland; 6 Department of Pediatrics, Oregon Health Sciences University, Portland, OR USA; 7 Present Address: Department of Pathology, Brigham and Women’s Hospital, Harvard Medical School, Boston, MA USA; 8 Present Address: Cancer Research UK Cambridge Institute, University of Cambridge, Cambridge, UK; 9 Present Address: Department of Hematology and Medical Oncology, Cleveland Clinic Taussig Cancer Institute, Cleveland, OH USA; 10 Present Address: Northwell Health Cancer Institute, Lenox Hill Hospital, New York, NY USA; 11 Present Address: Department of Education and Support for Regional Medicine, Tohoku University Hospital, Sendai, Japan; 12 Present Address: Dana Cancer Center, Toledo, OH USA

**Keywords:** Targeted therapies, Structure-based drug design, Cancer, Kinases, Mechanisms of disease

## Abstract

RET receptor tyrosine kinase is activated in various cancers (lung, thyroid, colon and pancreatic, among others) through oncogenic fusions or gain-of-function single-nucleotide variants. Small-molecule RET kinase inhibitors became standard-of-care therapy for advanced malignancies driven by RET. The therapeutic benefit of RET inhibitors is limited, however, by acquired mutations in the drug target as well as brain metastasis, presumably due to inadequate brain penetration. Here, we perform preclinical characterization of vepafestinib (TAS0953/HM06), a next-generation RET inhibitor with a unique binding mode. We demonstrate that vepafestinib has best-in-class selectivity against RET, while exerting activity against commonly reported on-target resistance mutations (variants in RET^L730^, RET^V804^ and RET^G810^), and shows superior pharmacokinetic properties in the brain when compared to currently approved RET drugs. We further show that these properties translate into improved tumor control in an intracranial model of RET-driven cancer. Our results underscore the clinical potential of vepafestinib in treating RET-driven cancers.

## Abstract (web summary)

Miyazaki et al. characterize vepafestinib, a next-generation RET inhibitor that is selective for wild-type RET and solvent front mutants. Due to a unique binding mode, it has enhanced brain penetrance and overcomes resistance to other RET inhibitors.

## Main

The rearranged during transfection (RET) protein belongs to the transmembrane receptor tyrosine kinase family and becomes an oncogenic driver when constitutively activated as a result of rearrangements and point mutations^1–4^. Fusion of the RET kinase domain with several N-terminal partners such as kinesin family 5B (KIF5B) or coiled-coil domain-containing 6 (CCDC6) occurs in approximately 70% of patients with RET fusion-positive non-small cell lung cancer (NSCLC)^5^. *RET* fusions are now considered as driver oncogenes in NSCLC, in which the prevalence is estimated to be 1–2% of unselected patients^6–9^. Earlier multi-kinase inhibitors (MKIs) such as cabozantinib and vandetanib have been tested in clinical trials for the treatment of *RET* fusion-positive NSCLC or medullary thyroid cancers (MTCs) with *RET* mutations^10–12^. However, clinical efficacy of MKIs has not reached expected outcomes, likely due to poor binding to RET and off-target interactions that may contribute to lower bioavailability in tumors and increased toxicity^13,14^.

The RET-selective inhibitors selpercatinib (LOXO-292) and pralsetinib (BLU-667), have shown durable clinical responses in patients with NSCLC and *RET* fusions, including some previously treated with MKIs or chemotherapy^15–17^, and their efficacy can be attributed to improved selectivity for RET compared to the MKIs used previously^17,18^. Selpercatinib and pralsetinib were approved in 2020 for patients with metastatic *RET* fusion-positive NSCLC, advanced or metastatic *RET*-altered MTC and papillary thyroid carcinoma. Despite early promising clinical benefits, recent reports describe RET solvent front (G810R, G810S, G810C), hinge (Y806C, Y806N) or ‘roof’ (L730) region mutations as mechanisms of acquired resistance to selpercatinib and/or pralsetinib^19–22^. Preclinical analysis of these mutations confirmed that current approved RET-selective inhibitors are less effective at inhibiting them^19,21,23^. Solvent front mutations are the most common type of resistance mutations occurring in approximately 40–50% of NSCLC driven by *ALK*, *NTRK1*, *NTRK2*, *NTRK3* and *ROS1* rearrangements^24,25^.

In addition to acquired secondary-drug-resistant mutations, brain metastases are another major clinical event contributing to disease progression in patients with NSCLC. For example, despite better control of intracranial disease in patients with *ALK*-rearranged NSCLC treated with second-generation anaplastic lymphoma kinase (ALK) inhibitors (for example, ceritinib and alectinib), relapse with central nervous system (CNS) progression during therapy remains common^26,27^. Thus, the high incidence of CNS progression and poor prognosis represents an unmet clinical need for cancers with kinase fusions^28^, as these patients are generally then treated with radiation or chemotherapy with known toxicities that limit quality of life.

Although favorable CNS responses have been reported in patients treated with selpercatinib or pralsetinib^29–31^, not all patients show response in the brain. A recent report has highlighted that over a quarter of patients treated with these drugs had both intracranial and extracranial disease progression^20^. Similarly, a more recent publication demonstrated that one-third of patients with baseline brain metastases suffered from CNS progression while on therapy with selpercatinib. Therefore, next-generation RET inhibitors with significantly improved CNS penetration over selpercatinib and pralsetinib would achieve better control of CNS disease, which may arise more frequently with long-term treatment. In this report, we describe the preclinical activity of vepafestinib (TAS0953/HM06), a next-generation selective RET inhibitor. Vepafestinib was specifically designed to be effective against RET wild-type (WT) kinase and RET solvent front mutants, and we demonstrate efficacy in preclinical models of brain metastasis. Vepafestinib is currently undergoing a phase 1–2 clinical trial to investigate its safety and efficacy in solid tumors with *RET* rearrangements (margaRET, NCT04683250).

## Results

### RET solvent front mutations are vulnerable to vepafestinib

**Fig. 1:**
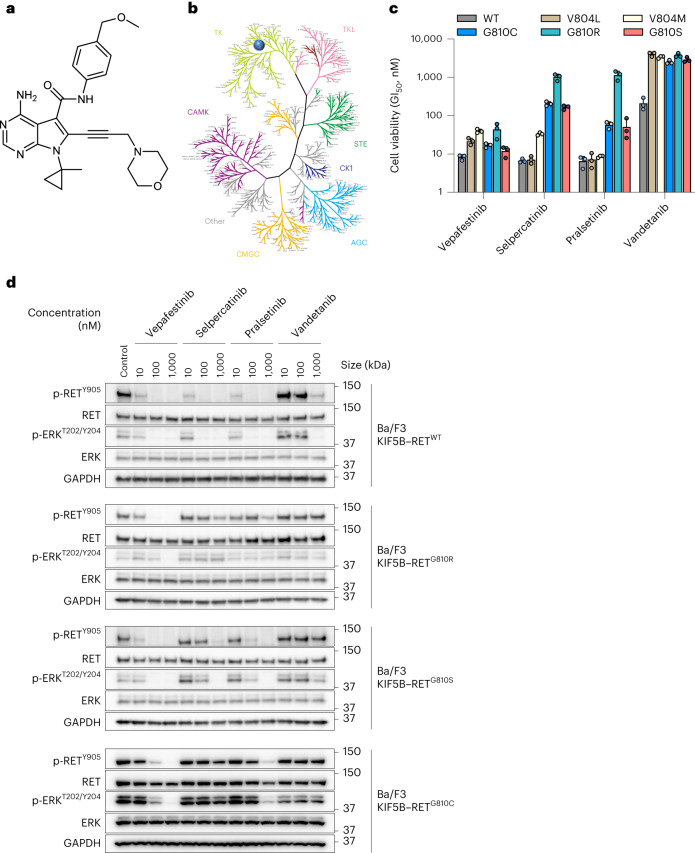
Structure and biochemical characterization of vepafestinib (TAS0953/HM06). **a**, Chemical structure of vepafestinib. **b**, Kinase selectivity profile of vepafestinib across 255 kinases. Enzyme activities were assessed in the presence of 23 nM vepafestinib, which is approximately 70-fold higher than the IC_50_ for inhibition of RET^WT^. Only one kinase (RET) was inhibited by >50% and is shown as a blue circle on the kinome tree. TK, tyrosine kinase; TKL, tyrosine kinase-like; CAMK, calcium/calmodulin-dependent protein kinase; STE, homologs of yeast sterile 7, sterile 11 and sterile 20 kinases; CK1, casein kinase 1; CMGC, cyclin-dependent kinases, mitogen-activated protein kinases, glycogen synthase kinases and cell division control protein-like kinases; AGC, protein kinase A, protein kinase G and protein kinase C families. **c**, GI_50_ (50% growth inhibition) values of vepafestinib, in comparison to other RET inhibitors on proliferation of Ba/F3 cells expressing KIF5B–RET^WT^ or KIF5B–RET harboring mutations in the solvent front of the kinase domain (G810R, G810S or G810C) or the gatekeeper domain (V804L or V804M). Data represent the mean ± s.d. of three independent experiments. **d**, Effect of vepafestinib on phosphorylation of RET and downstream signals in Ba/F3 cells expressing KIF5B–RET^WT^, KIF5B–RET^G810R^, KIF5B–RET^G810^^S^ or KIF5B–RET^G810^^C^. Cells expressing KIF5B–RET^WT^, KIF5B–RET^G810R^, KIF5B–RET^G810^^S^ or KIF5B–RET^G810^^C^ were treated with the indicated concentrations of each drug for 1 h before preparation of cell extracts for western blotting. Representative immunoblots from two independent experiments are shown. Glyceraldehyde-3-phosphate dehydrogenase (GAPDH) was used as a loading control. p, phosphorylated. Source data

**Extended Data Fig. 1:**
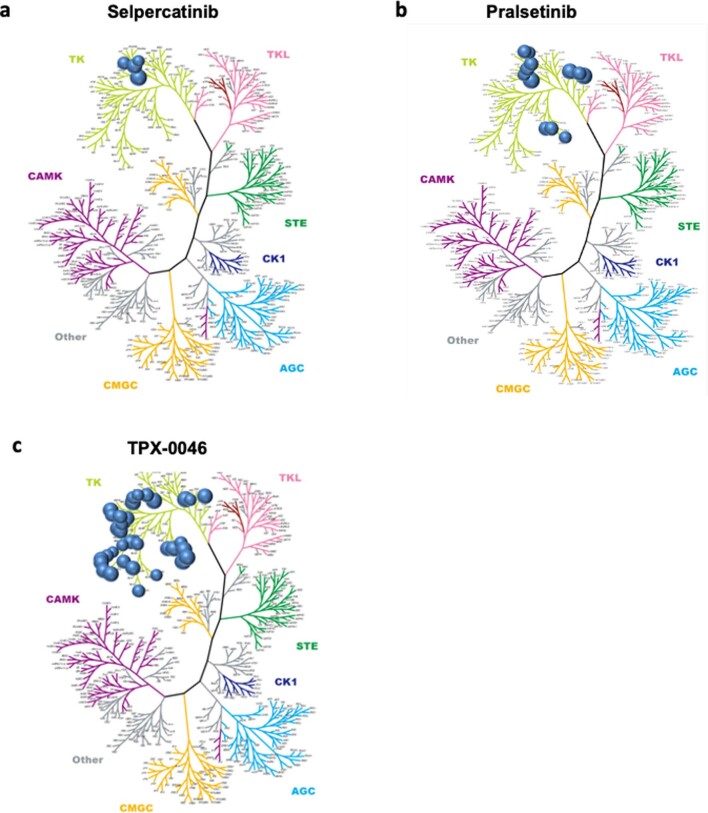
Selectivity profile of RET inhibitors. We performed kinase selectivity profile across 256 kinases in the presence of (**a**) 22 nM selpercatinib (**b**) 17 nM pralsetinib or (**c**) 26 nM TPX-0046/enbezotinib. These concentrations are approximately 100-fold higher than the corresponding IC_50_ value for inhibition of RET^WT^ enzymatic activity. Kinases that were inhibited by ≥50% by each small molecule are plotted as a circle on the kinome tree in the respective panel.

We employed rational chemical design to develop a potent and selective RET inhibitor and identified vepafestinib, a small molecule that is structurally distinct from existing RET inhibitors^18,32^. The alkyne moiety of vepafestinib (4-amino-*N*-[4-(methoxymethyl)phenyl]-7-(1-methylcyclopropyl)-6-[3-(morpholin-4-yl)prop-1-yn-1-yl]-7*H*-pyrrolo[2,3-*d*]pyrimidine-5-carboxamide) located in the 6-position on the 7*H*-pyrrolo[2,3-*d*]pyrimidine-5-carboxamide part of the structural core, resulted in a highly unique derivative in kinase inhibitors (Fig. 1a). Vepafestinib potently inhibited recombinant WT RET kinase at subnanomolar concentrations, similar to half-maximum inhibitory concentration (IC_50_) values obtained with selpercatinib or pralsetinib (IC_50_ values (nM): vepafestinib, 0.33 ± 0.01; pralsetinib, 0.31 ± 0.01; selpercatinib, 0.13 ± 0.03; vandetanib, 6.2 ± 0.8). A single concentration of 23 nM vepafestinib was tested on a panel of 255 recombinant kinases. RET was the only kinase inhibited by >50% (Fig. 1b and Supplementary Table 1a). Selpercatinib (22 nM) and pralsetinib (17 nM) were less specific, inhibiting three (including KDR (kinase insert domain receptor)) and 11 kinases by >50%, respectively (Extended Data Fig. 1a,b). These results were confirmed in dose–response studies of 14 kinases, in which KDR (also known as vascular endothelial growth factor receptor 2) was potently inhibited by selpercatinib (IC_50_ = 14 nM) and pralsetinib (IC_50_ = 35 nM) (Supplementary Table 1b). We also tested the RET and SRC family inhibitor TPX-0046 (enbezotinib, 26 nM) against a similar panel of kinases and found that TPX-0046 is an MKI, inhibiting 39 kinases by >50% (Extended Data Fig. 1c and Supplementary Table 2a). Targets of TPX-0046 included the kinases TRKA-C, FGFR1–FGFR4, most SRC family members, ACK and TXK (Supplementary Table 2a). The IC_50_ for inhibition of RET^WT^ by TPX-0046 was 0.26 ± 0.02 nM.

The cellular potencies of RET inhibitors against RET fusions and mutations, including RET^V804L^, RET^V804M^, RET^G810R^, RET^G810S^ and RET^G810C^ were evaluated using engineered Ba/F3 cells (Fig. 1c). Vepafestinib inhibited growth of Ba/F3 cells expressing KIF5B–RET^WT^ or KIF5B–RET mutants (V804M, V804L, G810R, G810S, G810C) (Fig. 1c). By contrast, growth of Ba/F3 cells expressing KIF5B–RET^G810R^, KIF5B–RET^G810^^S^ or KIF5B–RET^G810^^C^ was less sensitive to selpercatinib and pralsetinib than that of cells expressing RET^WT^, RET^V804M^ or RET^V804^^L^ as previously reported^19,21^. Vandetanib was less potent than the RET-selective inhibitors. Consistent with cell viability data, phosphorylation of RET and ERK were blocked by vepafestinib in Ba/F3 KIF5B–RET^WT^ cells (Fig. 1d). Of note, vepafestinib suppressed phosphorylation of RET^G810R^, RET^G810^^S^ and RET^G810^^C^ with near-complete inhibition at 100 nM (Fig. 1d). TPX-0046 inhibited phosphorylation of the RET^G810R^, RET^G810^^S^ and RET^G810^^C^ mutants, with RET^G810R^ being the least sensitive (IC_50_ values, RET^WT^, 21.9 nM; RET^G810R^, 108 nM) (Supplementary Table 2b). Selpercatinib and pralsetinib did not block phosphorylation of RET^G810R^, RET^G810^^S^ or RET^G810^^C^ (Fig. 1d).

### Crystal structure of RET complexed with selective inhibitors

**Fig. 2:**
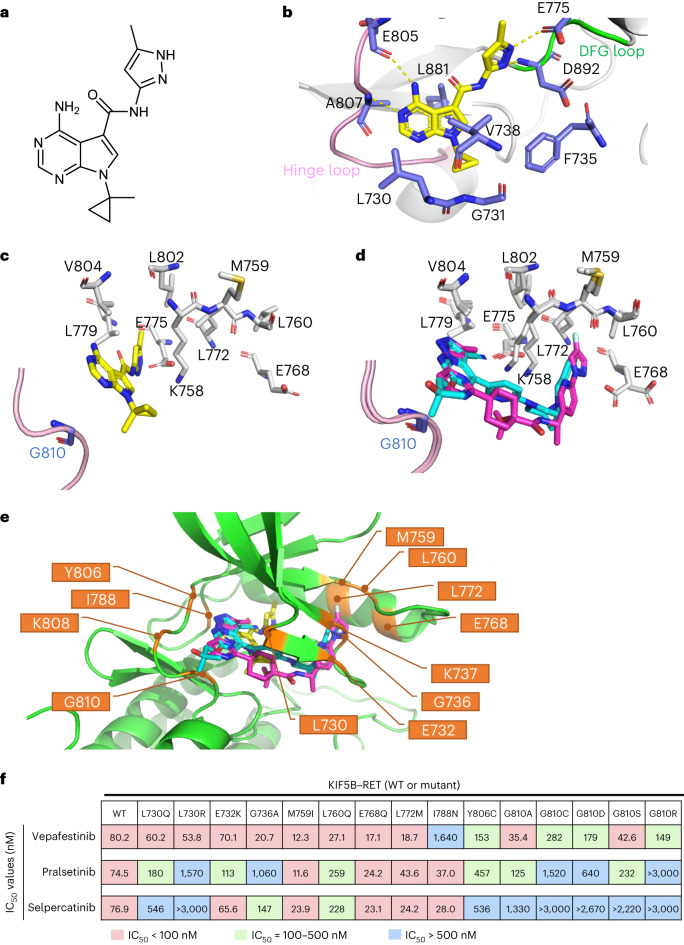
X-ray crystallography of RET complexed with RET-selective inhibitors. **a**, Chemical structure of TAS-C1. **b**, X-ray structure of RET complexed with TAS-C1. **c**, View from the solvent front area in the co-crystal structure of RET with TAS-C1. **d**, Overlay of co-crystal structures of selpercatinib and pralsetinib bound to RET. The viewpoint is the same as in **c**. The binding compounds are shown as stick models, with yellow (TAS-C1), cyan (selpercatinib) and magenta (pralsetinib) representing each RET inhibitor. **e**, Positions of the amino acid residues where mutagenesis was performed for in-cell western assays are shown in the co-crystal structure of RET with TAS-C1, overlaid with selpercatinib and pralsetinib. **f**, IC_50_ values calculated from in-cell western assays of Jump-In GripTite HEK293 cells transiently expressing WT or mutant KIF5B–RET. Cells were treated with the indicated compounds for 1 h. The assay was performed in triplicate, and mean IC_50_ values are represented with the color codes shown at the bottom.

**Extended Data Fig. 2:**
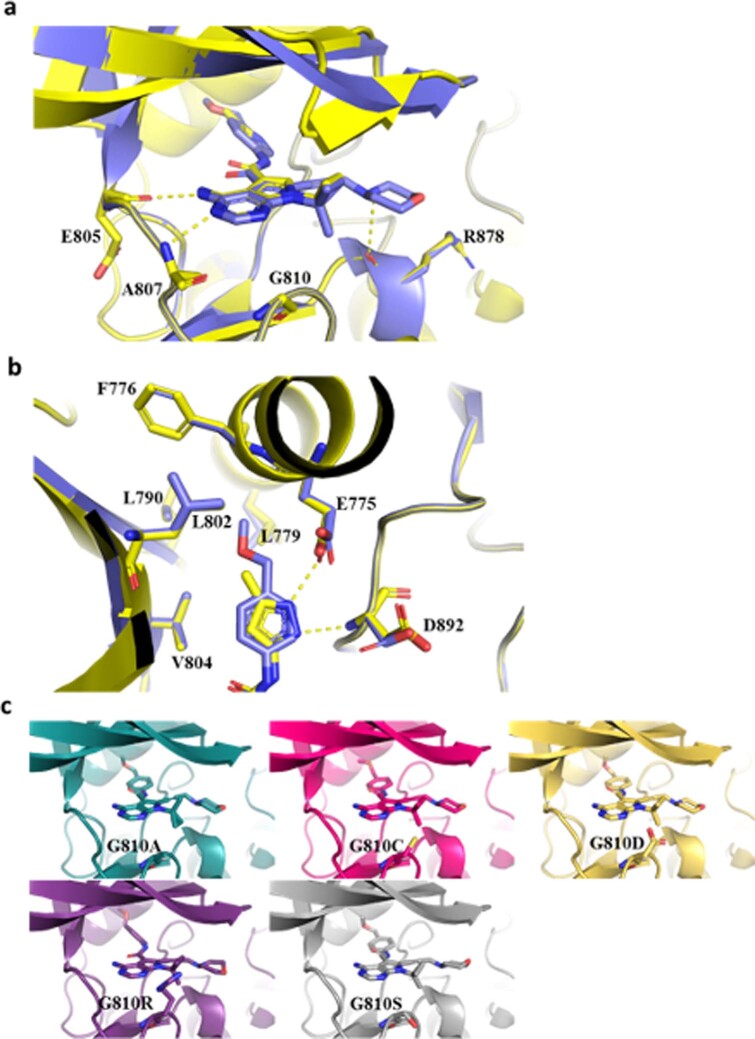
Docking study of vepafestinib. (**a**-**b**) The predicted model of vepafestinib in complex with wild-type RET (RET^WT^) (blue) superposed with the crystal structure of TAS compound 1 in complex with RET^WT^ (yellow). Panel (**b**) is focused on the surroundings of the methoxymethylbenzyl group of vepafestinib. Yellow dotted lines indicate the hydrogen bonds. (**c**) Predicted models of vepafestinib in complex with RET solvent front mutants (G810A, C, D, R, and S) based on molecular docking simulations were drawn from the same view-point as panel (**a**).

**Extended Data Fig. 3:**
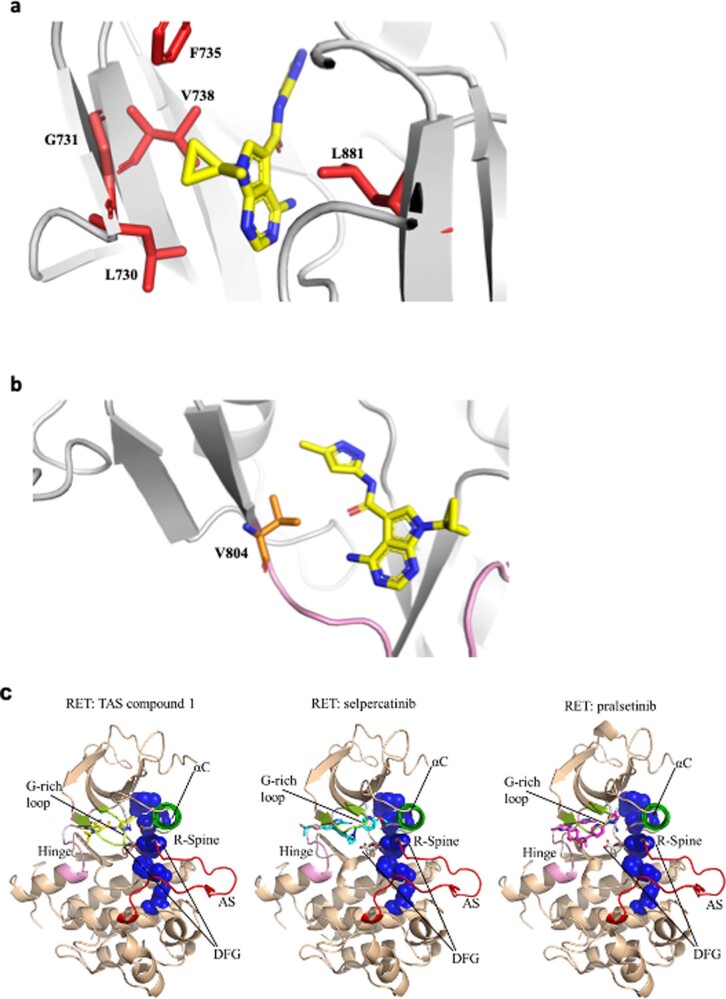
Binding pocket of RET. **(a)** X-ray crystal structure shows that TAS compound 1 fits into a pocket surrounded by L730, G731, F735, V738 and L881. (**b)** View from the gatekeeper residue (V804) in the X-ray structure of the RET-TAS compound 1 complex. TAS Compound 1 is shown as a stick model in yellow. **(c)** Crystal structures of human RET complexed with TAS compound 1, selpercatinib or pralsetinib. In all three structures, RET showed the active conformation; DFG-in, αC helix-in, Activation Segment-out, and R spine-liner. Therefore, the three drugs can potentially be classified as type I inhibitors.

The crystal structure of the RET kinase domain complexed with a vepafestinib analog, TAS compound 1 (TAS-C1) (Fig. 2a), was successfully solved at 1.64 Å. TAS-C1 was used because attempts to crystalize RET with vepafestinib were unsuccessful. Imposition of vepafestinib upon the TAS-C1–RET co-crystal structure showed substantial overlap of the two small molecules, suggesting that the data obtained with TAS-C1 could be extended to vepafestinib (Extended Data Fig. 2a,b). We also solved the crystal structures of RET complexed with selpercatinib and pralsetinib at 2.75 Å and 2.31 Å, respectively, in concordance with recently reported co-crystal structural data^33^. The pyrimidine ring in TAS-C1 forms hydrogen bonds with both E805 and A807 in the hinge region (Fig. 2b). In addition, nitrogen atoms in the pyrazole moiety in TAS-C1 forms hydrogen bonds with E775 and D892. At the opposite side, the cyclopropyl group occupies a hydrophobic environment, surrounded by L730, G731, F735, V738 and L881 (Extended Data Fig. 3a). The flexibility of the amide bond in TAS-C1 seems to be affected less sterically by the bulky substitutions of gatekeeper positions (V804) (Extended Data Fig. 3b). The methylpyrazole moiety of TAS-C1 is positioned in the pocket of the neighboring amino acids E775, L779, L802 and V804 (Fig. 2c). By contrast, the terminal moieties of the structures in selpercatinib and pralsetinib are inserted into another pocket surrounded by M759, L760, E768 and L772 (Fig. 2d). Additionally, TAS-C1 is positioned some distance away from the direction of the glycine side chain of the solvent front position 810, but selpercatinib and pralsetinib are closer (Fig. 2c,d). These findings indicate that substitution of glycine at codon 810 with other bulky residues is likely to establish steric hindrance for selpercatinib and pralsetinib but not for vepafestinib. This likely contributes to maintaining biological potency of vepafestinib toward RET^G810^ mutations.

Further analysis of the X-ray crystal structure revealed that there are roughly two clustering selpercatinib–RET or pralsetinib–RET complexes and the TAS-C1–RET complex in the point of the inserted area by the terminal moiety of these drugs. To assess the biological effects of these structural differences, we established a panel of RET mutations by substituting amino acids at positions close to the interaction site of each drug. We surmised that substitutions of amino acids that are in close proximity to a RET inhibitor when bound to the kinase may induce resistance to the respective drug. We identified nine residues in RET (E732, G736, K737, M759, L760, E768, L772, K808, G810) that have side chains or main chains within 4 Å of both selpercatinib and pralsetinib (Supplementary Table 3) and anticipated that substitution of these amino acids might influence binding of selpercatinib and pralsetinib but not vepafestinib. We also selected one residue (I788) with a side chain within 4 Å of TAS-C1 and hypothesized that substitutions at this site might reduce vepafestinib activity. Although two other residues (L730, Y806) are located within 4 Å of the three drugs, these residues form direct or indirect interactions with selpercatinib or pralsetinib. The positions of the 12 amino acids in the co-crystal structure of RET with the three drugs are shown in Fig. 2e. Subsequently, we established 15 potential mutations in the selected positions. Substituted amino acids were selected to generate previously reported RET mutations^19,33–39^ and/or to be larger or more charged than the original residue, which could affect RET–compound binding. Vepafestinib inhibited phosphorylation of RET^WT^ and most of the RET mutants (non-solvent front) with similar IC_50_ values (Fig. 2f). By contrast, phosphorylation of several RET mutants (L730Q, L730R, G736A, L760Q) was refractory to selpercatinib and pralsetinib compared to RET^WT^ phosphorylation. As predicted, RET^I788N^ conferred resistance to vepafestinib. Importantly, all RET^G810^ mutations remained vulnerable to vepafestinib. Although the RET^G810C^ mutant appeared about threefold less sensitive than RET^WT^, our data from Ba/F3 cells (Fig. 1c,d) imply that overcoming the RET^G810C^ mutation with vepafestinib is likely. All RET^G810^ mutations conferred decreased sensitivity to selpercatinib and pralsetinib (Fig. 2f) but resulted in sensitivity to TPX-0046 (Supplementary Table 2b). Further docking studies indicate that vepafestinib, pralsetinib and selpercatinib are likely to be type 1 inhibitors, based on predicted binding modes (Extended Data Fig. 3c).

### Vepafestinib blocks growth and signal transduction

**Fig. 3:**
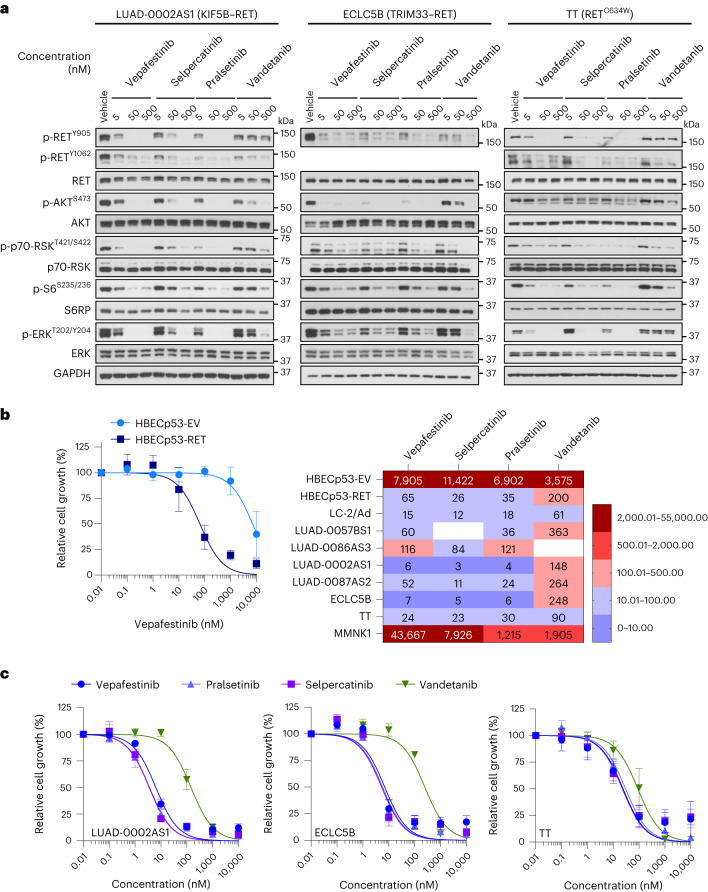
Vepafestinib inhibits transmission of signals and blocks growth of cells with *RET* alterations. **a**, LUAD-0002AS1, ECLC5B and TT cells were serum starved for 24 h and then treated with the indicated concentrations of vepafestinib (TAS0953/HM06), selpercatinib, pralsetinib or vandetanib for 2 h. Following treatment, whole-cell extracts were prepared and subjected to western blotting analysis. Representative immunoblots from two independent experiments are shown. GAPDH was used as a loading control. RSK, ribosomal protein S6 kinase; S6RP, S6 ribosomal protein. **b**,**c**, Cells were plated in 96-well plates and treated for 96 h with the inhibitors shown. The number of viable cells was assessed using alamarBlue. **b**, Viability curves for control HBEC cells (HBECp53-EV) and HBEC cells with the *CCDC6-RET* fusion (HBECp53-RET) are shown at the left. Results are the mean ± s.e.m. of four independent experiments. Data were analyzed by non-linear regression, and IC_50_ values were estimated by curve fitting. A heatmap of the IC_50_ values is shown on the right. Missing values indicate that the experiment was not done. **c**, Viability curves for LUAD-0002AS1 (*n* = 3), ECLC5B (*n* = 3) and TT (*n* = 5) cells. Results are mean ± s.e.m. Each condition was assayed in triplicate for all viability studies. Source data

**Extended Data Fig. 4:**
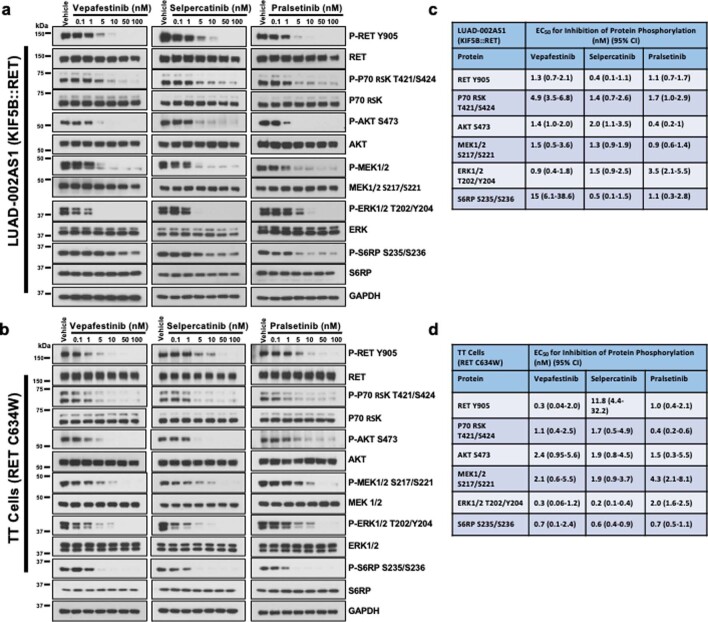
Inhibition of protein phosphorylation by RET-selective inhibitors. Cells were deprived of serum for 24 h before treatment with the indicated concentrations of inhibitors for 2 h. Whole-cell extracts were then prepared, resolved by SDS-PAGE and immunoblotted for the total or phosphorylated (P) protein shown. (**a-b)** Representative immunoblots from two independent Western blotting analysis are displayed. GAPDH was used as a loading control. (**c-d**) Blots were quantitated by densitometry and then the ratio of phosphorylated protein to total protein was analyzed by non-linear regression using Graphpad Prism v9 software to find the EC_50_ for inhibition of phosphorylation. Data represent the mean of two independent measurements with the 95% confidence interval (CI) shown in brackets. On each immunoblot, the vehicle-treated control was considered 100% phosphorylation and all other conditions are repressed relative to this. These values were adjusted for any change in protein expression by dividing by the corresponding total protein relative densitometry reading. Source data

Serum-starved cells were treated with 5, 50 or 500 nM inhibitor for 2 h, and then protein phosphorylation levels were examined (Fig. 3a). Exposure of LUAD-0002AS1 (NSCLC, KIF5B–RET), ECLC5B (NSCLC, tripartite motif-containing 33 (TRIM33)–RET) and TT cells (medullary thyroid carcinoma, RET^C634W^) to vepafestinib resulted in efficient downregulation of RET phosphorylation at Y905 and Y1062 and downstream effectors. Near-complete inhibition of phosphorylation was achieved with 50 nM vepafestinib, similar to results with selpercatinib and pralsetinib. Vandetanib was less effective. We performed additional dose–response western blotting studies using lower inhibitor concentrations. Immunoblots were quantitated by densitometry, and the EC_50_ for phosphorylation inhibition was estimated (Extended Data Fig. 4). We confirmed that vepafestinib was as effective as selpercatinib and pralsetinib at inactivating RET signaling in LUAD-0002AS1 (Extended Data Fig. 4a) and TT (Extended Data Fig. 4b) cells. Quantitation of immunoblots is shown in Extended Data Fig. 4c,d.

**Extended Data Fig. 5:**
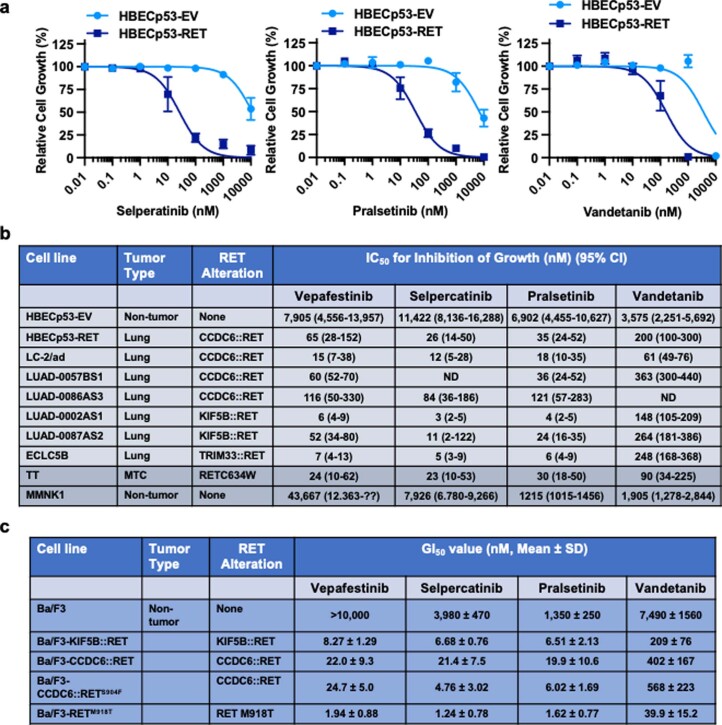
Sensitivity of tumor and non-tumor cells to RET inhibitors. Human cells were plated at a density of 7,500 cells per well in 96-well plates and treated with inhibitors for 96 h. Growth was assessed using alamarBlue. Ba/F3 cell lines were plated at a density of 1,000 cells per well in 96-well plates, treated with inhibitors for 72 h and growth was then determined using CellTiter-Glo assay. **(a)** Selected growth curves. Data represent the mean ± SEM of 3 (selpercatinib) or 4 (pralsetinib and vandetanib) independent experiments in which there were three replicates of each condition. **(b)** The mean growth inhibition data was analyzed by non-linear regression and curve fitted to obtain estimated IC_50_ values and the 95% confidence interval (CI). Five (TT), 4 (HBECp53-EV, HBECp53-RET, LUAD-0057BS1, LUAD-0087AS1), 3 (LUAD-0002AS1, ECLC5B) or 2 (LC-2/ad, MMNK1) independent experiments in which each condition was assayed in triplicates were conducted. Data for LUAD-0086AS3 represent one experiment in which there were 3 replicates of each condition **(c)** GI_50_ values are represented by the mean ± SD of 3 independent experiments. The S904F substitution in the activation loop of RET was previously shown to confer resistance to vandetanib. Source data

Next, we examined the efficacy of vepafestinib in blocking growth of 12 tumor cell lines (patient -derived and isogenic) that are models of *RET* fusions or *RET* mutations found in NSCLC and thyroid cancers and three nontumor cell lines. Vepafestinib effectively inhibited growth of HBECp53-RET (*CCDC6-RET* fusion; IC_50_ = 60 nM) but had little effect on the isogenic control HBECp53-EV cells at concentrations <1,000 nM (IC_50_ = 7,905 nM) (Fig. 3b). This result was comparable to those obtained with pralsetinib and selpercatinib (Extended Data Fig. 5a,b). Similarly, vepafestinib inhibited growth of LUAD-0002AS1 cells (Fig. 3c and Extended Data Fig. 5b) and Ba/F3 cells expressing RET fusions (KIF5B–RET, CCDC6–RET, CCDC6–RET^S904F^)^40^ or the RET^M918T^ mutation (Extended Data Fig. 5c). Vepafestinib was more effective at inhibiting growth of all tumor cell lines than vandetanib and as effective as selpercatinib and pralsetinib (Fig. 3b, right, Fig. 3c and Extended Data Fig. 5). No RET inhibitor showed preference toward any of the three RET fusions in our study. The nontumor cholangiocyte cell line MMNK1 was more sensitive to selpercatinib, pralsetinib and vandetanib than to vepafestinib (Extended Data Fig. 5b).

### Vepafestinib modulates growth and survival pathways

**Fig. 4:**
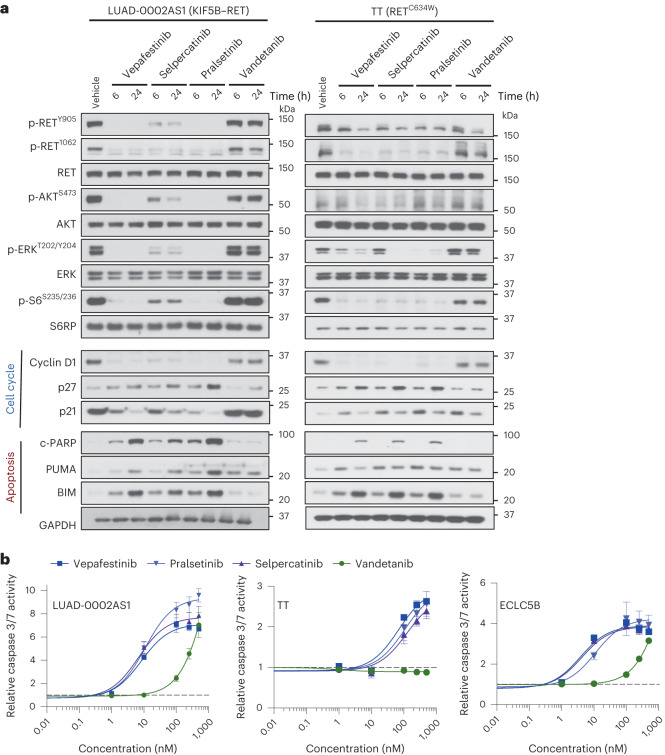
Vepafestinib modulates expression of cell cycle and apoptosis markers. **a**, LUAD-0002AS1 and TT cells were serum-staved for 24 h and then treated with 100 nM vepafestinib (TAS0953/HM06), selpercatinib, pralsetinib or vandetanib for 24 h. Following treatment, whole-cell extracts were prepared and subjected to western blotting analysis. Representative immunoblots from two independent experiments are shown. GAPDH was used as a sample-processing control. **b**, Cells were treated with the indicated RET inhibitors for 48 h before measuring caspase 3 and 7 enzymatic activity in cell homogenates. Results represent the mean ± s.d. of two independent experiments in which each condition was assayed in triplicate. Source data

**Extended Data Fig. 6:**
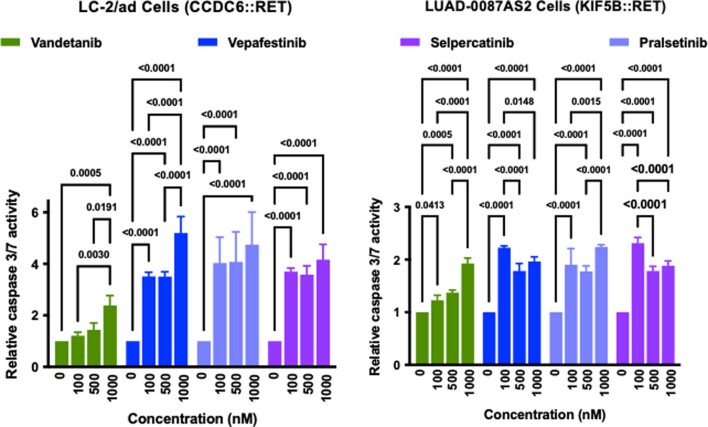
Caspase 3/7 activity in LC-2/ad and LUAD-0087AS2 NSCLC cells. Cells were plated at a density of 20,000 cells per well in 96-well plates and with the indicated concentrations of RET inhibitors for 48 h. Results represent the mean ± SD of three replicates in one experiment. Data were compared using Two-way ANOVA with Tukey’s multiple comparison tests. All tests were two sided. Source data

To gain further insight into the mechanism by which vepafestinib inhibited growth, we assessed expression of markers of cell cycle progression and apoptosis in cells treated with inhibitors. In LUAD-0002AS1 cells, vepafestinib caused almost complete inhibition of RET, AKT, S6, ERK1 and ERK2 phosphorylation after 6 h of treatment, and this was maintained for up to 24 h (Fig. 4a). Similar results were obtained with TT cells. Sustained treatment of LUAD-0002AS1 and TT cells with vepafestinib and other RET-selective inhibitors resulted in downregulation of the cell cycle regulator cyclin D1 and increased expression of the cell cycle inhibitor p27. Treatment of LUAD-0002AS1 cells (p53 mutant) with vepafestinib resulted in downregulation of the cell cycle inhibitor p21; however, the opposite was observed in TT cells (p53 WT). Expression of apoptosis markers such as cleaved PARP (c-PARP), BIM and PUMA was induced in all cell lines by 6 h. The results obtained with vepafestinib were similar to those obtained with selpercatinib and pralsetinib. Vandetanib was less effective at blocking expression of cyclin D1 and increasing expression of cell cycle inhibitors and pro-apoptotic proteins (Fig. 4a). Exposure to vepafestinib resulted in dose-dependent increases in caspase 3 and 7 activity in the five lung cancer cells tested (Fig. 4b, LUAD-0002AS1, TT, ECLC5B; Extended Data Fig. 6, LC-2/ad, LUAD-0087AS2). The degree of caspase 3 and 7 stimulation by vepafestinib was similar to that observed with selpercatinib and pralsetinib treatment.

### Vepafestinib blocks growth of *RET* fusion models in vivo

**Fig. 5:**
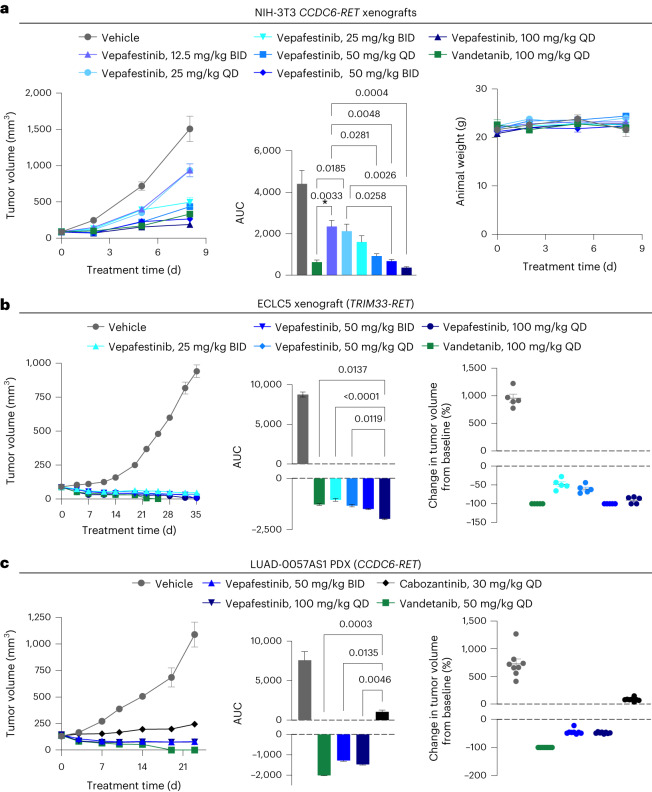
Efficacy of vepafestinib in RET fusion-dependent disease models in vivo. Cell lines (NIH-3T3 expressing CCDC6–RET, ECLC5) or PDX tumors were implanted into subcutaneous flanks of female mice and treated as indicated. **a**, NIH-3T3-RET xenograft (athymic nude mice). **b**, ECLC5 xenograft (NOD–SCID gamma (NSG) mice). **c**, LUAD-0057AS1 PDX. **a**–**c**, Left, time course of treatment. Data represent mean ± s.e.m. There were five (NIH-3T3-RET and ECLC5 xenografts) or eight (LUAD-0057AS1) animals per group. **a**–**c**, Middle, AUC analysis of tumor growth. Data represent mean ± s.e.m. of *n* = 12 (NIH-3T3-RET), *n* = 32–44 (ECLC5) or *n* = 46–49 (LUAD-0057AS1) values per group. **a**, Right, animal weight. **b**,**c**, Right, percent change in the volume of individual tumors at the end of the study. Mean ± s.e.m. are shown. The volume of tumors in all treatment groups in each model was significantly lower than that of the respective vehicle-treated groups (*P* < 0.0001). *P* values for statistical significance are shown for other comparisons (ANOVA with Dunnett’s multiple-comparison test). All tests were two sided. Source data

**Extended Data Fig. 7:**
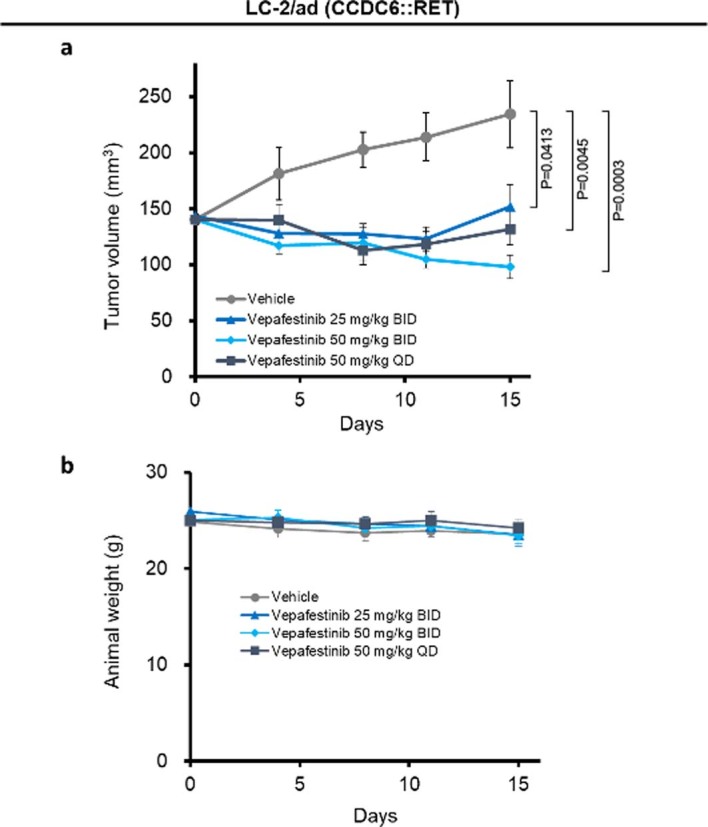
Anti-tumor activity of vepafestinib against LC-2/ad NSCLC xenograft model. **(a)** Tumor volume. Data are shown as mean ± SEM (n = 6 or n = 4 animals per group). Dunnett test was used for comparison. All statistical tests were two-sided. Vepafestinib or vehicle were administered orally at the indicated doses, twice daily (BID) or once daily (QD) for 14 days (Day 1-14) after grouping. (b) Animal weight was measured twice weekly. Source data

**Extended Data Fig. 8:**
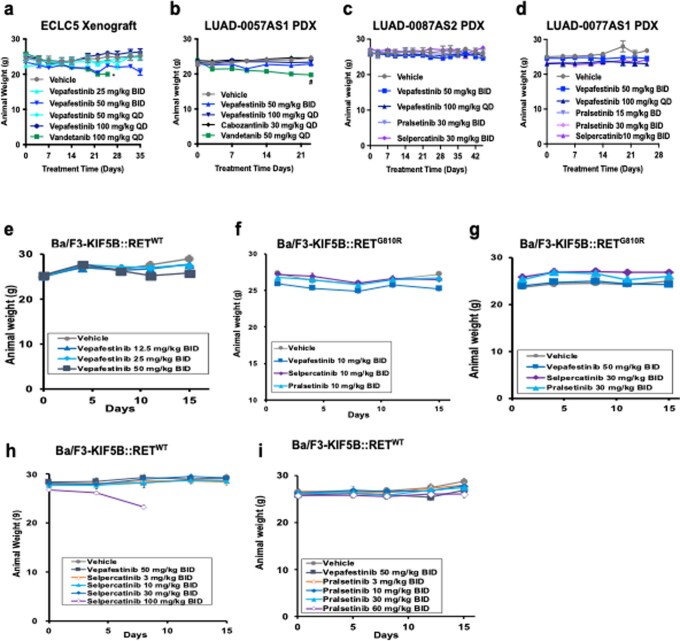
Effect of RET inhibitors on weight of tumor-bearing animals. Mice bearing (**a**) ECLC5 xenograft (n = 5), (**b**) LUAD-0057AS1 PDX (n = 8), (**c**) LUAD-0087AS2 PDX (n = 5) or (**d**) LUAD-0077AS1 (n = 5) were treated with RET multi-kinase or selective inhibitors. Mice bearing allograft tumors derived from Ba/F3 cells expressing KIF5B::RET^WT^ (**e**, n = 6) or KIF5B::RET^G810R^ (**f** and **g**, n = 5) fusions were administrated vepafestinib (TAS0953/HM06), selpercatinib or pralsetinib. Mice bearing Ba/F3-KIF5B::RET^WT^ allograft tumors were treated with vehicle, selpercatinib or pralsetinib (**h** and **i**, n = 5). Drugs or vehicle were administered orally at the indicated doses, once daily (QD) or twice daily (BID). Data is presented as mean ± SEM in each group. Tumor-bearing animals were weighed twice weekly *P = 0.0046, compared to the weight on day 0. ^#^P = 0.0015, compared to weight on day 0. Data were compared by ANOVA with Dunnet’s multiple comparison tests. All tests were two sided. Source data

We next examined vepafestinib efficacy in vivo. Mice implanted with NIH-3T3-RET (NIH-3T3 cells expressing *CCDC6-RET* fusion complementary DNA), ECLC5B or LC-2/ad (*CCDC6-RET*) cells, or LUAD-0057AS1 (*CCDC6-RET*) patient-derived xenograft (PDX) tumors were treated with various dosages of vepafestinib, or vandetanib or cabozantinib (Fig. 5). Cabozantinib was used as a control drug for LUAD-0057AS1 cells, as the PDX model was derived from tumor tissue of a patient with poor response to cabozantinib. Tumor growth is shown on the left; area under the curve (AUC) analysis is shown in the middle; the percent change in individual tumor volume from baseline is shown on the right (Fig. 5). Administration of vepafestinib resulted in a dose-dependent decrease in growth of NIH-3T3-RET xenograft tumors (Fig. 5a, left), with all dosages of vepafestinib tested resulting in a significant reduction in tumor volume (Fig. 5a, middle). There was no statistically significant reduction in animal weight for any of the treatments (Fig. 5a, right). Similarly, vepafestinib treatment resulted in a significant reduction in LC-2/ad tumor growth, with substantial tumor regression observed with the 50 mg per kg twice daily (BID) dosage (Extended Data Fig. 7a). There was no statistically significant reduction in animal weight with any vepafestinib dosage (Extended Data Fig. 7b). Vepafestinib treatment caused significant reductions in ECLC5B xenograft tumor growth (Fig. 5b, left), with 50 mg per kg BID and 100 mg per kg once daily (QD) dosing resulting in 100% ± 0% and 90.3% ± 4% tumor regression, respectively. Vandetanib treatment inhibited tumor growth significantly (*P* < 0.0001), with all tumors shrinking (Fig. 5a, left). However, vandetanib-treated animals showed significant weight loss (*P* = 0.01) and were killed early. No dosage of vepafestinib had any adverse effect on animal health or animal weight (*P* > 0.05) (Extended Data Fig. 8a). Treatment of mice bearing LUAD-0057AS1 PDX tumors with vepafestinib also resulted in significant reductions in tumor volume (Fig. 5c, left). Tumors shrank by 44% ± 3% and 48% ± 1% when treated with 50 mg per kg BID or 100 mg per kg QD vepafestinib, respectively. As expected in this model, cabozantinib slowed growth but did not lead to any tumor shrinkage at a dosage that has been shown to completely inhibit growth of RET fusion-driven xenograft tumors (30 mg per kg QD)^41^, while vandetanib and vepafestinib treatment caused substantial tumor regression (Fig. 5c, middle and right). Vandetanib (50 mg per kg QD) caused a significant reduction in animal weight (*P* = 0.0015) (Extended Data Fig. 8b). No dosage of vepafestinib or the other RET-selective inhibitors had any adverse effect on animal health or animal weight (*P* > 0.05) (Extended Data Fig. 8). These results suggest that vepafestinib is effective at reducing tumor growth, including in a model that was refractory to cabozantinib.

**Fig. 6:**
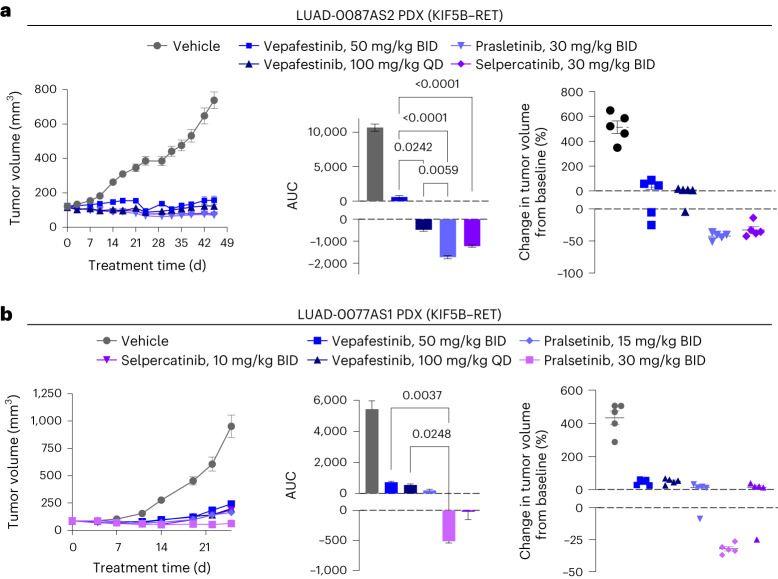
Efficacy of vepafestinib compared to other RET-selective inhibitors in PDX models. **a**, LUAD-0087AS2 PDX. **b**, LUAD-0077AS1 PDX. **a**,**b**, Left, time course of treatment. Data represent mean ± s.e.m. There were five mice in each group in both models. **a**,**b**, Middle, AUC analysis of tumor growth. Data represent mean ± s.e.m. of *n* = 56 (LUAD-0087AS2) or *n* = 32 (LUAD-0077AS1) values per group. **a**,**b**, Right, percent change in the volume of individual tumors at the end of the study. Mean ± s.e.m. are shown. Each group consisted of five animals. The volume of tumors in all treatment groups in each model was significantly lower than that of the respective vehicle-treated groups (*P* < 0.0001). *P* values for significance are shown for other comparisons (ANOVA with Dunnett’s multiple-comparison test). All tests were two sided. Source data

**Extended Data Fig. 9:**
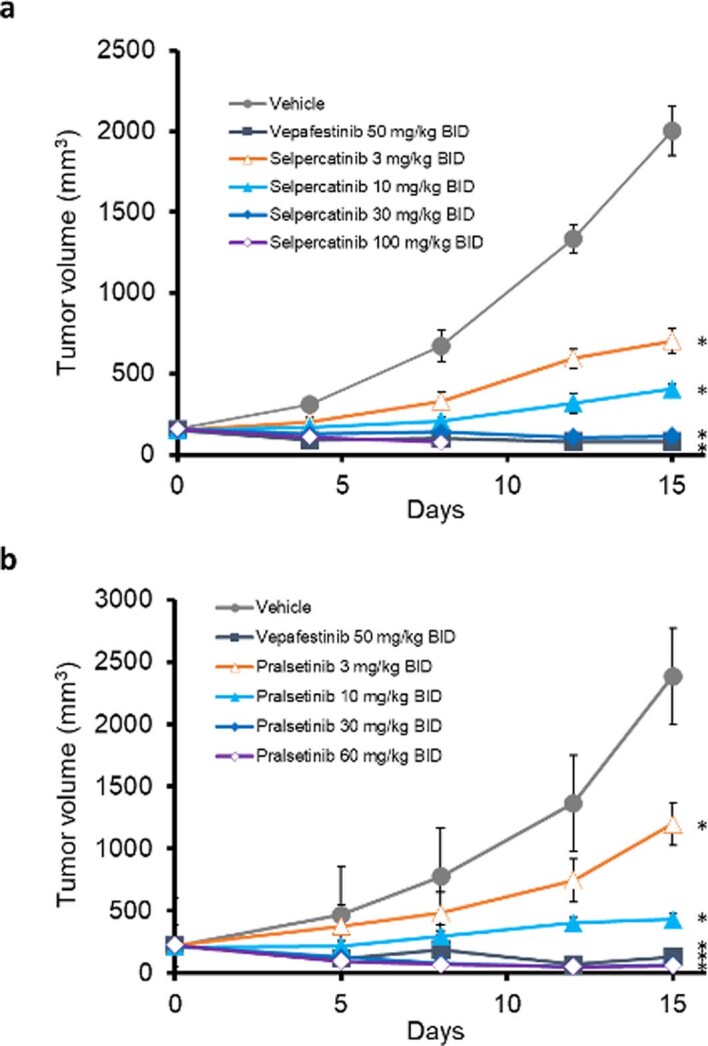
Anti-tumor activity of vepafestinib in comparison with selpercatinib or pralsetinib. Animals bearing Ba/F3-KIF5B::RET^WT^ allograft tumors were treated with (**a**) vehicle, vepafestinib or selpercatinib or with (**b**) vehicle, vepafestinib or pralsetinib. Data shown is the mean ± SEM (n = 5 per group).* P < 0.05 compared with the control group by Dunnett test. Tests were two-sided. Drugs or vehicle were administrated orally at the indicated doses, twice daily (BID) for 14 days (Day 1-14) after grouping. Source data

We expanded our efficacy studies to include two additional NSCLC PDX models with RET fusions. We compared vepafestinib to selpercatinib and pralsetinib, both of which have been shown to inhibit growth of RET fusion-driven tumors in vivo at dosages of 10 mg per kg BID or less^17,18^. Vepafestinib treatment also caused significant reductions in tumor growth in LUAD-0087AS2 PDX (Fig. 6a) and LUAD-0077AS1 PDX (Fig. 6b) models. None of the RET-selective inhibitors caused any change in animal health or weight (*P* > 0.05) (Extended Data Fig. 8c–i). In a Ba/F3 KIF5B–RET allograft tumor model, 50 mg per kg BID vepafestinib was as efficacious as 30 mg per kg selpercatinib and 60 mg per kg pralsetinib in reducing tumor burden (Extended Data Fig. 9).

### RET^G810R^ in vivo models remain susceptible to vepafestinib

**Fig. 7:**
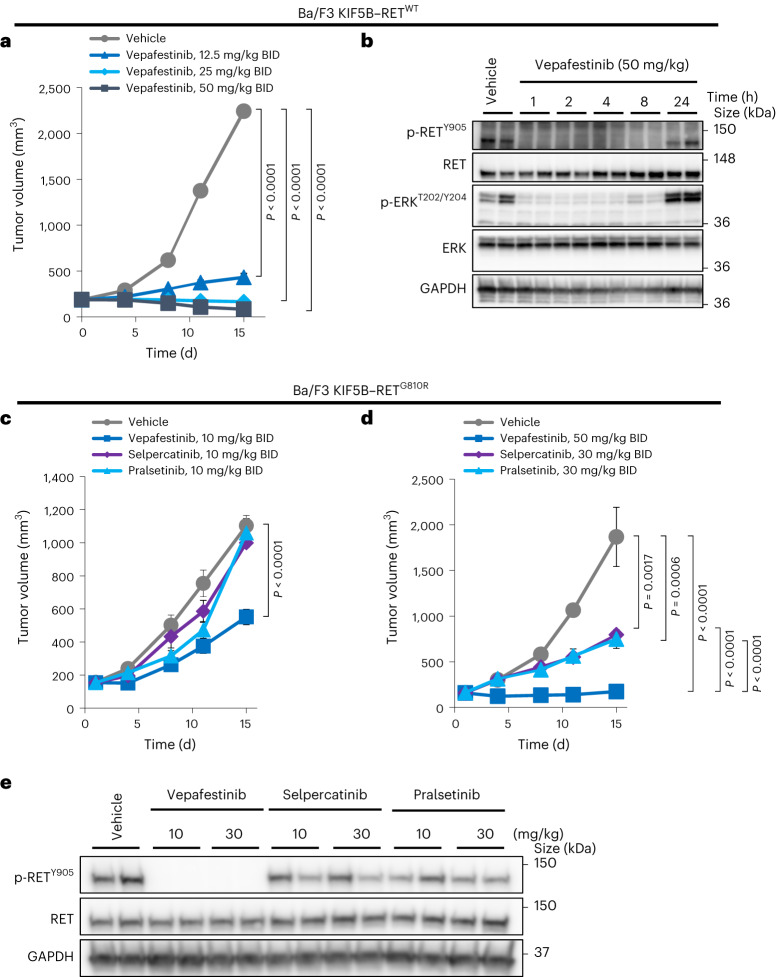
Anti-tumor activity of vepafestinib against KIF5B–RET^G810R^-driven allograft tumors. **a**, Animals bearing Ba/F3 KIF5B–RET^WT^ allograft tumors were treated with vehicle (*n* = 6) or the indicated dosages of vepafestinib (*n* = 6). **b**, Animals bearing Ba/F3 KIF5B–RET^WT^ tumors were treated with a single dose of 50 mg per kg vepafestinib, and then tumors were collected at the indicated time points after inhibitor administration for western blotting analysis. Representative immunoblots on which two tumors from each condition were examined are shown. **c**,**d**, Mice bearing Ba/F3 KIF5B–RET^G810R^ xenograft tumors were administered vepafestinib (*n* = 5), selpercatinib (*n* = 5), pralsetinib (*n* = 5) or vehicle (*n* = 5) orally at the indicated dosages BID for 14 d (days 1–14) after grouping. **e**, Mice bearing Ba/F3 KIF5B–RET^G810R^ allograft tumors were administered 10 or 30 mg per kg vepafestinib, selpercatinib or pralsetinib, and then tumors were collected 1 h later for western blot analysis. Representative immunoblots on which two tumors from each condition were examined are shown. Tumor volume for each dosing group was measured and shown as mean ± s.e.m. Statistical analysis was performed using Dunnett’s test (vehicle versus vepafestinib, selpercatinib or pralsetinib) or Tukey’s test (vepafestinib versus selpercatinib or pralsetinib), and *P* values are shown. All tests were two sided. GAPDH was used as a loading control in **b**,**e**. Source data

To address vepafestinib potency against RET^G810R^ in vivo, we examined the ability of the drug to block growth of Ba/F3 KIF5B–RET^WT^ or Ba/F3 KIF5B–RET^G810R^ allograft tumors. Treatment of Ba/F3 KIF5B–RET^WT^ allograft tumors with vepafestinib (12.5, 25, 50 mg per kg BID) resulted in dose-dependent inhibition of tumor growth (Fig. 7a) without any body weight changes (Extended Data Fig. 8e). To assess target engagement in vivo, tumor-bearing animals were given a single dose of vepafestinib (50 mg per kg), and then tumors were extracted at various time points. Western blot analysis showed that vepafestinib completely inhibited phospho-RET and phospho-ERK for at least 8 h after drug administration (Fig. 7b). At an equivalent dosage (10 mg per kg BID), vepafestinib was more effective than selpercatinib and pralsetinib at slowing growth of Ba/F3 KIF5B–RET^G810R^ allograft tumors (Fig. 7c). The identical dosage of selpercatinib and pralsetinib, however, caused substantial reduction in growth of Ba/F3 KIF5B–RET^WT^ tumors (Extended Data Fig. 9a,b). Administration of 50 mg per kg BID vepafestinib had a significant anti-tumor effect on Ba/F3 KIF5B–RET^G810R^ tumors without any animal body weight changes (Fig. 7d and Extended Data Fig. 8f,g). Consistent with the anti-tumor activity, vepafestinib completely inhibited RET^G810R^ phosphorylation in tumors treated with doses of 10 mg per kg and 30 mg per kg (Fig. 7e). Although the highest dosage of selpercatinib and pralsetinib (30 mg per kg BID) showed moderate anti-tumor effect against Ba/F3 KIF5B–RET^G810R^ allograft tumors (Fig. 7d), there was not a commensurate decrease in phosphorylation of the RET^G810R^ mutant (Fig. 7e), suggesting that these effects may be due to off-target effects.

### Vepafestinib exhibits high CNS availability

**Fig. 8:**
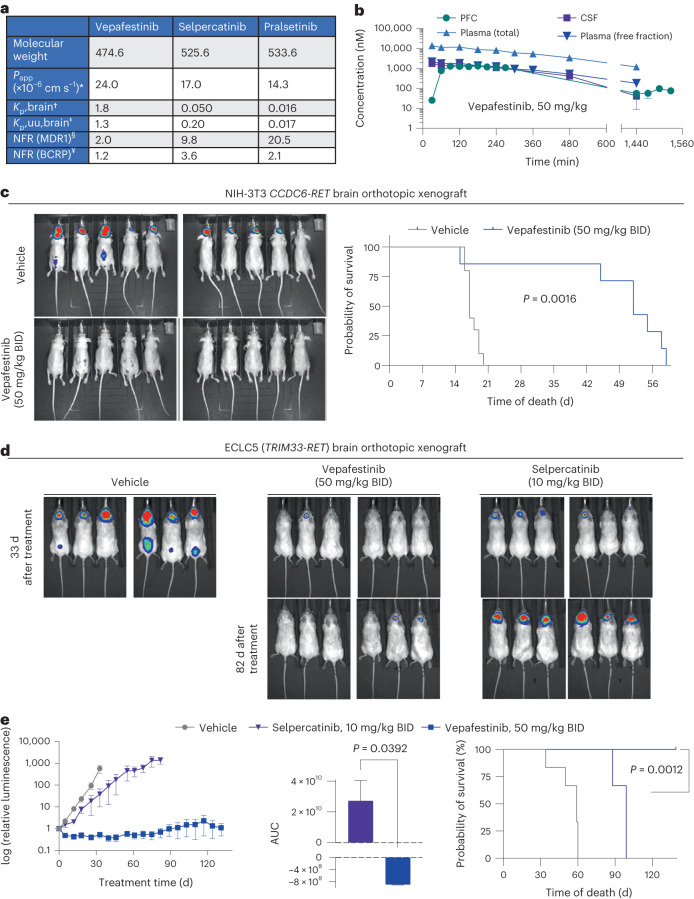
Vepafestinib is more effective than selpercatinib at penetrating the brain and blocking intracranial tumor growth. **a**,**b**, Pharmacokinetic properties. **a**, *Apparent permeability coefficient (*P*_app_) values were calculated as the mean of *P*_app_ values in the apical-to-basal direction in mock-transfected LLC-PK1 cells. ^†,‡^Total (*K*_p,brain_) and unbound (*K*_p,uu,brain_) brain/plasma concentration ratios were calculated based on total and unbound concentrations in plasma and brain at 0.5 h or 1 h after oral administration of each agent to male BALB/c mice dosed with 50 mg per kg drug. Unbound fractions in plasma (fu,plasma) and brain (fu,brain) were obtained by the equilibrium dialysis method with plasma and brain homogenate. ^§,¥^Net flux ratio (NFR) values for MDR1 (P-gp) and BCRP were obtained from transcellular transport assays using control or MDR1-expressing LLC-PK1 cells and control and BCRP-expressing MDCK II cells. **b**, Single-dose vepafestinib (3 mg per kg, 10 mg per kg or 50 mg per kg) was administered orally to male Han Wistar rats at time = 0 min (*n* = 12 per dosing group). Following equilibration, samples were collected at the indicated time points, and vepafestinib concentrations were then determined. Data for all dosages are shown in Extended Data Fig. 10. Data represent mean ± s.e.m. (*n* = 4 independent measurements in four animals). **c**, NIH-3T3 *CCDC6-RET* cells harboring a luciferase reporter were implanted intracranially into nude mice and treated with vehicle or 50 mg per kg vepafestinib BID. Treatment started 5 d after implantation. Bioluminescence images of animals 13 d after implantation are shown (left). Survival curves of each group are shown after implantation (*n* = 10, vehicle group; *n* = 7, vepafestinib group) (right). There was a significant difference in survival between the vehicle group and the vepafestinib group (*P* = 0.0016, log-rank test). **d**, ECLC5 cells labeled with a luciferase reporter were implanted intracranially into NSG mice and treated with vehicle, selpercatinib (10 mg per kg) or vepafestinib (50 mg per kg) BID. Treatment started 10 d after implantation. There were six animals in each group. **d**, Bioluminescence images of animals are shown for the last day when all animals were alive in the three groups (43 d after implantation) and at 92 d after implantation for the two treatment arms. **e**, Luciferase signals were quantified and are shown (left). Data represent mean ± s.e.m. (*n* = 6 per group). AUC analysis was performed for the selpercatinib and vepafestinib groups (middle, Brown–Forsythe and Welch ANOVA tests). For AUC, data represent mean ± s.e.m. of *n* = 100 (vepafestinib) or *n* = 65 (selpercatinib) values. Survival curves are shown for animals after treatment began (right). Treatment with selpercatinib (*P* = 0.0008, log-rank test) and vepafestinib (*P* = 0.0008, log-rank test) increased survival relative to the vehicle. However, animals treated with vepafestinib had longer survival (*P* = 0.001, log-rank test). All statistical tests were two sided. Source data

We designed vepafestinib to have enhanced blood–brain barrier (BBB) penetration and retention. Here, we assessed pharmacological and pharmacokinetic properties of vepafestinib, including membrane permeability, transport by efflux transporters and brain penetrance. The key pharmacological characteristics of vepafestinib, selpercatinib and pralsetinib are illustrated in Fig. 8a. The three RET inhibitors showed excellent membrane permeability but different susceptibility to efflux transporters. MDR1 (P-glycoprotein; P-gp) and breast cancer resistance protein (BCRP) are two major efflux transporters expressed at the BBB, where they prevent entry of many endogenous substances and chemicals into the CNS^42^. Vepafestinib showed low net flux ratio for P-gp and BCRP (Fig. 8a). By contrast, selpercatinib and pralsetinib were higher affinity substrates for P-gp; selpercatinib also showed slight substrate susceptibility for BCRP. Substances with *K*_p,uu,brain_ value > 0.3 in mice are regarded as favorable brain-penetrating agents^43,44^. Vepafestinib showed relatively high *K*_p,brain_ and *K*_p,uu,brain_ values in mice (1.8 and 1.3, respectively), while the values for selpercatinib and pralsetinib were <0.3 in mice. We also examined the same parameters for TPX-0046 and found that this compound was a substrate for P-gp and is expected to have poor BBB permeability based on its *K*_p,uu,brain_ of 0.077 (Supplementary Table 2c). These results indicate that vepafestinib concentrations in the brain would be better maintained than those of selpercatinib, pralsetinib and TPX-0046.

**Extended Data Fig. 10:**
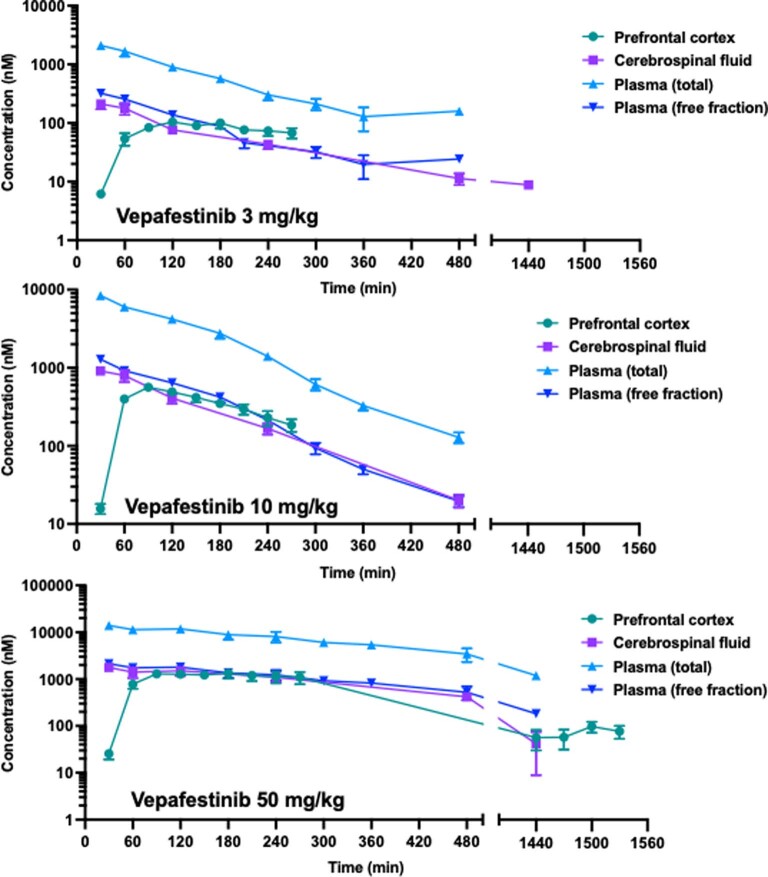
Pharmacokinetics of vepafestinib in rats. A Single-dose of 3 mg/kg (**top panel**), 10 mg/kg (**middle panel**) or 50 mg/kg (**bottom panel**) vepafestinib was administered orally to adult male Han Wistar rats at time = 0 min. Following equilibration, samples were collected at the indicated time points and vepafestinib concentrations were then determined by HPLC with tandem mass spectrometry (MS/MS) using D_8_-vepafestinib as the internal standard as described in Supplementary Methods. Data from panel **c** is also displayed in Fig. 8b and is included here for comparison with the other dosages used. There were 12 animals in each dosage group, and prefrontal cortex, CSF and plasma samples were obtained from 4 animals within each dosage group. The x-axis is broken to accommodate all the time points in the space. No data was eliminated between the breaks. Source data

We characterized the pharmacokinetics of vepafestinib in the prefrontal cortex (PFC), cerebrospinal fluid (CSF) and plasma of freely moving adult male Han Wistar rats following single-dose oral administration at 3, 10 and 50 mg per kg (Fig. 8b and Extended Data Fig. 10a,b). Once equilibrium was achieved between the compartments, the ratio of the observed concentrations of vepafestinib in microdialysates from the PFC, CSF and plasma-free fraction was close to 1:1:1. The concentrations were maintained from 2 h to 6.5 h after vepafestinib administration (up to 8 h for CSF) (Fig. 8b and Extended Data Fig. 10). The 1:1 concentration ratio of free plasma to free brain concentration indicates that vepafestinib readily crosses the BBB and that the free plasma concentration of vepafestinib is a good approximation of the free concentration in the PFC and CSF.

### Vepafestinib is highly effective in controlling CNS disease

We examined vepafestinib efficacy in an orthotopic allograft model of brain metastasis. NIH-3T3-RET cells were labeled with a luciferase construct to enable bioluminescence imaging and implanted into the brains of mice, and then treatment commenced 5 d later. As seen in Fig. 8c (left), vepafestinib-treated animals had no detectable tumors and showed significantly longer survival (median, 52 d) than vehicle-treated animals (median, 17 d; *P* = 0.0016) (Fig. 8c, right).

Given the high brain penetrance and CNS efficacy seen with vepafestinib in Fig. 8a–c, we decided to perform a comparison with selpercatinib in an orthotopic NSCLC model of CNS disease. ECLC5B cells expressing a luciferase construct were implanted into the brains of mice, and treatment commenced 10 d later. Tumor growth was suppressed significantly by vepafestinib with a long period of tumor regression. By contrast, ECLC5B tumors continued to grow in the CNS of animals treated with selpercatinib, although less than that observed with vehicle treatment (Fig. 8d and Fig. 8e, left). Tumor burden at the end of selpercatinib treatment was significantly higher than that in vepafestinib-treated animals (Fig. 8e, middle). Animals treated with vepafestinib had a significantly longer survival time (all animals were alive after 139 d of treatment) than animals treated with selpercatinib (median, 99 d of treatment) (Fig. 8e, right).

## Discussion

While tyrosine kinase inhibitors (TKIs) have proven to be effective targeted therapy for cancers arising from kinase gene rearrangements, relapse due to acquired on-target resistance represents a substantial therapeutic limitation. More than half of acquired resistance in ALK fusion-targeted therapy is caused by on-target mutations, of which the solvent front mutation ALK^G1202R^ is predominant^45^. In RET-targeted therapies, the emergence of solvent front substitutions (RET^G810R^, RET^G810S^, RET^G810C^) has been reported in patients who relapsed after selpercatinib or pralsetinib therapy^19,20,33^. The reported incidence of RET^G810^ mutations in clinical samples is 10% (ref. ^20^). In this report, we describe vepafestinib, which was rationally designed to be effective against RET^WT^ and gatekeeper (RET^V804^) and solvent front (RET^G810^) mutants and has properties that will enhance BBB penetration. We show that vepafestinib exhibited greater inhibitory activity against RET^WT^ and RET^V804^ and RET^G810^ mutants in vitro (RET^G810C^, RET^G810^^R^ and RET^G810^^D^ were 2–3-fold less sensitive than RET^WT^) and showed less off-target activity than selpercatinib, pralsetinib and TPX-0046 in our kinase profiling. Consistent with these findings, vepafestinib suppressed growth of allograft tumors harboring the RET^G810R^ mutation (Ba/F3 KIF5B–RET^G810R^) and displayed substantial efficacy against Ba/F3 cells expressing *CCDC6-RET* fusions (RET^WT^ and RET^S904F^) or the RET^M918T^ mutation found in MTC. Vepafestinib also inhibited the growth of multiple lung cancer patient-derived cell lines harboring RET fusions with different N-terminal partners (CCDC6, KIF5B, TRIM33) and a RET^C634W^-mutation-positive MTC cell line. Furthermore, vepafestinib was effective at inhibiting growth of five NSCLC xenograft models. Our data suggest that vepafestinib would have broad activity against RET solvent front mutations as well as across various RET mutations and fusions, regardless of fusion partners, in a tumor-agnostic fashion.

We solved the crystal structure of TAS-C1 (a vepafestinib derivative), selpercatinib and pralsetinib bound to RET. It is generally known that kinase inhibitors can be classified into type I–VI based on the structures of their drug–enzyme complexes^46^. From our crystal structures, TAS-C1, selpercatinib and pralsetinib were bound to RET in the active conformation (DFG (Asp-Phe-Gly) residues-in/αC-helix-in conformer) similar to the previously reported vandetanib-bound RET^47^. Therefore, TAS-C1, selpercatinib and pralsetinib are likely type I inhibitors. The co-crystallographic data on RET–TAS-C1 reveal that TAS-C1 does not fill the space close to the solvent front position, suggesting that substitution of the glycine with other large bulky amino acids is unlikely to institute steric hindrance between TAS-C1 and RET. Indeed, vepafestinib retained biological activity against various solvent front substitutions such as RET^G810R^, RET^G810S^, RET^G810A^, RET^G810C^ and RET^G810D^, with the RET^G810C^ mutant being about threefold less sensitive than RET^WT^. Structural modeling studies predict that TAS-C1 and vepafestinib bind to RET^WT^ with similar binding modes, with the phenyl group of vepafestinib inserting into the deep hydrophobic pocket flanked by residues E775, F776, L779, L790, L802 and V804. As the structure of the ATP-binding pocket of RET^G810A^ is reported to be highly similar in shape and position to that of RET^WT^ (ref. ^48^), we next performed docking simulations of vepafestinib on RET solvent front mutations. Our data suggest that there is a space between the cyclopropyl moiety of vepafestinib and the substituted amino acids, and this results in escape from the substitution effects. From the co-crystallographic data analysis, we found that RET-selective drugs could be classified into two groups: (1) selpercatinib and pralsetinib with a similar binding mode in the RET pocket, where the terminal parts of the drugs are positioned in the pocket surrounding M759, L760, E768 and L772; and (2) TAS-C1 with a completely unique binding mode, where the neighboring amino acids are E775, L779, L802 and V804. Screening of RET mutants indicated overlapping resistance profiles between selpercatinib and pralsetinib, with RET^L730Q^, RET^L730R^, RET^G736A^, RET^L760Q^, RET^G810R^, RET^G810S^, RET^G810A^, RET^G810C^ and RET^G810D^ conferring resistance. Contrastingly, vepafestinib inhibited these selpercatinib- and pralsetinib-resistant mutants. These findings suggest cross-resistance between selpercatinib and pralsetinib but not between vepafestinib and these two agents, indicating that vepafestinib may offer advantages over Food and Drug Administration-approved RET inhibitors currently in clinical use.

The CNS is a common site of relapse for patients with NSCLC treated with TKIs. However, designing kinase inhibitors with considerable BBB penetration remains challenging. In general, compounds with good brain penetration in animal models are more likely to exhibit good CNS penetration in humans^49^. Additionally, avoiding efflux transport is key to achieving good CNS penetration due to overexpression of drug efflux transporters in the BBB^44,49,50^. Vepafestinib, which was designed for CNS penetration, showed high preclinical brain exposure and low propensity for P-gp and BCRP transport. By contrast, brain penetration of selpercatinib and pralsetinib is limited in mice, and both drugs are P-gp and/or BCRP substrates, consistent with data in recent reports^51,52^. Importantly, we showed in this study that vepafestinib was superior to selpercatinib in controlling CNS disease in an orthotopic model of NSCLC brain metastasis. The limited BBB penetration and brain exposure may account for CNS metastasis reported in selpercatinib- and pralsetinib-refractory patients^20^. Moreover, it was recently shown that brain metastasis was the only form of disease progression in a patient with RET fusion-driven sarcoma treated with selpercatinib^53^. The increased CNS availability of vepafestinib has the potential to provide substantial benefits for patients with RET fusion-driven disease who eventually relapse due to brain metastases.

Although many TKIs have been developed as therapies, achieving highly selective kinase inhibition is key to success^54^. Kinase fusion-positive cancers have appreciably fewer mutations than other cancers, including in known cancer-related genes, suggesting that the growth of these tumors is strongly dependent on oncogenic fusion^55,56^. Therefore, more selective drugs could be ideal for kinase fusion-targeted therapy. We show that vepafestinib is a highly selective RET inhibitor with no detectable off-target activity. Selpercatinib and pralsetinib, on the other hand, inhibited several kinases such as KDR or JAKs with IC_50_ values in the subnanomolar range. KDR inhibition may contribute to the moderate anti-tumor efficacy of selpercatinib and pralsetinib in animals bearing Ba/F3 RET^G810R^ allograft tumors, given the lack of target engagement observed. Consistent with the excellent selectivity of vepafestinib, growth of three untransformed cell lines remained unaffected when exposed to the inhibitor. TPX-0046 is a recently disclosed RET inhibitor with activity against SRC and RET solvent front mutations but not RET gatekeeper mutations^57^. We confirmed that TPX-0046 inhibited RET^WT^ at subnanomolar concentrations (IC_50_ = 0.26 ± 0.02 nM) and was highly effective against various RET mutations including G810 substitutions. However, TPX-0046 showed limited brain penetrability (*K*_p,uu,brain_ = 0.077). Importantly, TPX-0046 inhibited a broad range of kinases including the three TRK isoforms, the four FGFR isoforms, many SRC family members, ACK, TXK, etc. and therefore should be considered an MKI along the lines of vandetanib, cabozantinib and RXDX-105. We believe that the superior selectivity of vepafestinib would contribute to a clinically wider therapeutic index than that of TPX-0046.

There are several limitations to this study. First, the crystal structures of RET complexes were performed with a vepafestinib analog (TAS-C1), as crystallization of RET with vepafestinib was not successful. However molecular docking simulation revealed that vepafestinib and TAS-C1 bind to RET in an almost identical manner. Second, we relied on molecular docking simulation to model binding to vepafestinib. Although we believe that we modeled the interaction of vepafestinib with RET^G810A^, RET^G810^^C^, RET^G810^^D^, RET^G810^^R^ and RET^G810^^S^ with high confidence, this does not replace the accuracy that would be obtained with crystallographic studies with mutant kinases. Third, we examined vepafestinib efficacy mainly in subcutaneous xenograft models where tumors are contained and may not faithfully represent patient tumor burdens where disease is present at multiple sites with different degrees of blood flow. A similar limitation exists for the studies examining CNS efficacy in which we used an orthotopic xenograft model in which a bolus of tumor cells was implanted directly into the brain. This model may not fully recapitulate the clinical situation in which tumor cells likely arrive in the brain as single cells and interact distinctly with the microenvironment. Nevertheless, any limitation of our tumor models applies equally to the data obtained with vepafestinib and selpercatinib.

RET-independent resistance mechanisms would render selpercatinib- or pralsetinib-refractory patients unamenable to vepafestinib treatment. Despite these exceptions, we believe that vepafestinib has the potential to offer a valuable therapeutic option to patients with RET fusions, including those with resistance to first-generation RET-selective inhibitors, given its potency and superior brain-penetration kinetics. Future studies will assess the combination of vepafestinib with inhibitors of bypass pathways to address the clinical need arising from these resistance mechanisms.

In summary, vepafestinib is a pharmacologically advanced next-generation RET inhibitor exhibiting a distinct binding mode to RET. In this report, we show that vepafestinib had potent inhibitory activity against WT RET and RET gatekeeper (V804) and solvent front (G810) mutations in vitro, with less off-target activity than selpercatinib, pralsetinib and TPX-0046 (enbezotinib). Consistent with in vitro data, vepafestinib showed superior efficacy in tumor allografts derived from Ba/F3 cells expressing RET^WT^ or RET^G810R^ fusion proteins. The increased CNS availability of vepafestinib, the superior efficacy in preclinical CNS disease models and the broad activity against RET solvent front mutations, as well as across various RET fusions regardless of N-terminal partners in NSCLC and in MTC models represent a possible effective strategy to overcome the emergence of acquired resistance to first-generation RET-selective inhibitors. Vepafestinib is currently in a phase 1–2 trial for patients with solid tumors driven by *RET* alterations (NCT04683250).

## Methods

All research presented in this study complies with all ethical regulations and was approved by the Institutional Review Board of the Memorial Sloan Kettering (MSK) Cancer Center (MSKCC) (for biospecimen collection), the MSK Institutional Animal Care and Use Committee and Research Animal Resource Center (for animal studies) and the Institutional Animal Care and Use Committee of Taiho Pharmaceutical (for Ba/F3 subcutaneous allograft and NIH-3T3 intracranial allograft studies). The maximum allowed tumor burden was 2 cm^3^. This limit was not exceeded in any study described in this paper. Animals used in this study were cared for in accordance with the Guide for the Care and Use of Laboratory Animals. One to five mice per cage were kept in individually ventilated caging systems where the temperature was 21.1–22.2 °C, humidity was 30–70%, and a 12-h light cycle was maintained.

### Reagents and cell lines

Vepafestinib (TAS0953/HM06), TAS-C1 and pralsetinib (BLU-667) were synthesized by Taiho Pharmaceutical following the synthetic scheme in the patent applications WO2017043550, WO2017146116 and WO2017079140. Vandetanib used in Ba/F3 studies was purchased from LC Laboratories. Vandetanib (used for all other studies) and cabozantinib were obtained from Selleckchem. Selpercatinib (LOXO-292) was purchased from Sundia MediTech. TPX-0046 was purchased from DC Chemicals. Each compound was dissolved in dimethyl sulfoxide (DMSO) for cell culture experiments. Ba/F3 cells were purchased from the RIKEN BioResource Center (RCB4476). Ba/F3 cells stably expressing WT or mutant *KIF5B-RET* were generated by transfection of the appropriate expression vectors (see the Supplementary Methods for additional details) and were grown in RPMI-1640 medium containing l-glutamine, phenol red, HEPES and sodium pyruvate, supplemented with 10% FBS. Jump-In GripTite HEK293 cells were purchased from Thermo Fisher Scientific (A14150) and grown in high-glucose DMEM medium containing GlutaMAX and pyruvate, supplemented with 25 mM HEPES, 0.1 mM MEM non-essential amino acids, 100 U ml^−1^ penicillin, 100 µg ml^−1^ streptomycin and 10% dialyzed FBS. Human LLC-PK1 cells (mock transfected, 450216) or ones transfected with MDR1 (MDR1-LLC-PK1, 450211) were obtained from Discovery Labware and were grown in Medium 199 supplemented with 0.05 mg ml^−1^ gentamicin, 100 μg ml^−1^ hygromycin B, 2 mM l-glutamine and 7% FBS. Parental MDCK II cells or cells expressing BCRP (BCRP-MDCK II) were obtained from the Netherlands Cancer Institute and were grown in DMEM supplemented with 100 U ml^−1^ penicillin, 100 µg ml^−1^ streptomycin, 2 mM l-glutamine and 10% FBS. The ECLC5B, LUAD-0002AS1 and NIH-3T3-RET cell lines were generated as described previously^41^ and were grown in DMEM/F12 (high-glucose) medium supplemented with 10% FBS and 100 µg ml^−1^ Primocin (InvivoGen). The LC-2/ad cell line was obtained from the RIKEN BioResource Center (RCB0440) and grown in RPMI-1640 medium supplemented with 10% FBS and 100 µg ml^−1^ Primocin. MMNK1 cholangiocytes were purchased from the JCRB Cell Bank (JCRB1554). Cell lines were frequently tested for mycoplasma (3–4 months), and no cell line used in this study tested positive. Cell lines purchased from cell banks were STR verified by the provider before purchase, and multiple vials were cryopreserved by investigator laboratories. While conducting studies, a new vial of the respective cells was thawed and used up within 2 months, and known genetic markers (for example, *RET* fusion) were verified by PCR at least once during the use of that stock. Cell lines generated at the MSKCC were genomically characterized by MSK-IMPACT, and fusion oncogenes were verified by PCR each time a new cryopreserved vial was thawed.

### Generation of patient-derived xenograft models and cell lines and efficacy studies

Tissue samples were collected under an MSKCC IRB-approved biospecimen-collection protocol (protocols 06-107 and 12-245), and informed consent was obtained. All animals were monitored daily and cared for in accordance with guidelines approved by the MSK Institutional Animal Care and Use Committee and Research Animal Resource Center (protocol 04-03-009) or the Institutional Animal Care and Use Committee of Taiho Pharmaceutical (protocols 18TB17, AE18-414, AE18-611, AE19-168, AE19-460, AE19-603 and AE19-613). Pleura effusion fluid samples (LUAD-0057BS1 and LUAD-0087AS2) were obtained from patients undergoing therapeutic thoracentesis. Heparin was added (10 USP units per ml fluid) immediately after collection. Cells were isolated by centrifugation and injected subcutaneously into the flank of 6-week-old female NSG mice (Jackson Laboratory) to generate xenografts as described previously^58^. To generate the LUAD-0057BS1 and LUAD-0087AS2 cell lines, 50 million cells were plated in 150-cm^2^ tissue culture flasks in DMEM/F12 medium supplemented with 10% FBS and 100 µg ml^−1^ Primocin. Cell lines were considered established after being passaged 20 times. Tumor samples (LUAD-0077AS1) were obtained from biopsy procedures, cut into small pieces, mixed with Matrigel and implanted subcutaneously into the flank of female NSG mice. The presence of the respective *RET* fusions was verified by PCR.

For in vivo efficacy studies, all tumors or cell lines were implanted subcutaneously into the flanks. Fresh PDX tumor or ECLC5B xenograft tumor samples were implanted into flanks of female NSG mice. To generate NIH-3T3-RET allografts, 5 million cells were injected into flanks of 6-week-old female athymic nude mice (Envigo) subcutaneously. The flanks of 6-week-old male BALB/c nude mice (CLEA Japan) were implanted subcutaneously with Ba/F3 cells engineered to stably express KIF5B–RET^WT^ or KIF5B–RET^G810R^ (5 × 10^6^ cells per mouse). Mice were randomized by tumor size into groups of four to eight when tumor volume reached approximately 100–150 mm^3^, and treatment was initiated on a 5-d on, 2-d off schedule or on a daily dosing. For intracranial studies, 100,000 ECLC5 or 25,000 NIH-3T3-RET cells (both labeled with a luciferase construct) were injected into the brain of animals. For the NIH-3T3-RET intracranial study, three mice in the vepafestinib group were excluded from the survival analysis due to accidental death. No statistical methods were used to predetermine sample size, but our sample sizes are similar to those reported in previous publications^53,58^. See the Supplementary Methods for more details. Cabozantinib was resuspended in 30% propylene glycol, 5% Tween-80 and 65% dextrose solution. Vandetanib suspension was made in 1% sodium carboxymethyl cellulose. Vepafestinib (TAS0953/HM06), pralsetinib (BLU-667) and selpercatinib (LOXO-292) were resuspended in 0.1 M HCl and 0.5% hypromellose. All compounds were administered by oral gavage. Tumor size and body weight were measured two times each week, and tumor volume was calculated with the following formula: (length × (width)^2^) × 2^−1^. For western blotting analysis of allografts, tumors were resected from mice after drug treatment, and extracts were immunoblotted as described below.

### Growth inhibition and the caspase 3 and 7 activity assay

Ba/F3 cells expressing KIF5B–RET or CCDC6–RET (WT or S904F) or RET^M918T^ were plated in 96-well plates (1,000 cells per well) and treated with inhibitors for 72 h. Cell viability was assessed by luminescence using the CellTiter-Glo 2.0 Assay (Promega). GI_50_ values (the concentration that exerted 50% growth inhibition compared with that of the untreated controls) were calculated using a sigmoidal dose–response model in the XLfit 5 add-in for Microsoft Excel (ID Business Solutions). Data are presented as mean ± s.d. of three independent experiments. Patient-derived cells were seeded in 96-well plates (7,500 cells per well) and treated with inhibitors for 96 h. alamarBlue viability dye was used to estimate growth as described previously^59^. IC_50_ values were determined by curve fitting using GraphPad Prism. For caspase 3 and 7 activity, cells were plated at a density of 20,000 or 30,000 (TT cells) cells per well in 96-well plates, grown for 48 h (NSCLC cells) or 72 h (TT cells), and then caspase 3 and 7 enzymatic activity was determined using the Apo-One Homogeneous Caspase-3/7 activity assay kit (Promega). All viability data are expressed relative to control values and are an average of three to five independent experiments, where each condition was assayed in triplicate determinations. For caspase assays, data are expressed relative to control values and are an average of two (LUAD-0002AS1, ECLC5B, TT cells) independent experiments, where each condition was assayed in triplicate determinations. For LUAD-0087AS2 and LC-2/ad cells, data represent the mean ± s.d. of three replicates in one experiment.

### Immunoblotting

See Supplementary Table 4 for a complete list of antibodies and dilutions used. Ba/F3 cells were lysed in Cell Extraction Buffer (Sample Diluent Concentrate 2, Bio-Techne), and patient-derived cells were lysed in radioimmunoprecipitation buffer; lysis buffers were supplemented with phosphatase (PhosSTOP) and protease inhibitors (cOmplete Mini Protease Inhibitor Cocktail), both obtained from Merck. Total cellular proteins (10 µg for Ba/F3 cells, 20 µg for Ba/F3 xenografts or 25 µg for other cells) were subjected to SDS–PAGE. After electrophoresis, the separated proteins were transferred to PVDF membranes (Bio-Rad Laboratories), and then membranes were blocked in Blocking One-P (Nacalai Tesque), before incubation overnight with primary antibodies on a shaker in a cold room. The next day, membranes were washed and then soaked with HRP-linked anti-rabbit IgG (Cell Signaling Technology). The bands of the target proteins were detected with SuperSignal West Dura Extended Duration Substrate (Thermo Fisher Scientific) by the Amersham Imager 600 QC (Cytiva) or exposed to X-ray film and visualized using a Kodak X-ray developer.

### RET^WT^ kinase-inhibition assay

Enzymatic kinase-inhibitory activities of vepafestinib (TAS0953/HM06), pralsetinib and selpercatinib were detected using purified recombinant human RET. See the Supplementary Methods for additional details.

### Kinase selectivity profiling

Kinase activity of 255 recombinant kinases (vepafestinib) or 256 kinases (all other inhibitors) was assessed in the presence of inhibitors and was carried out by Carna Biosciences, according to their product instructions. See the Supplementary Methods for additional details.

### Transcellular transport study

MDR1-LLC-PK1, LLC-PK1, BCRP-MDCK II and MDCK II cells were plated in the inserts of a BD Falcon 96-Multiwell Insert System (1-μm pore, PET membrane, Corning) and cultured in an incubator at 37 °C with 5% CO_2_ for 4 d. After washing the cell monolayer on each insert with transport buffer (Hank’s Balanced Salt Solution supplemented with 10 mM HEPES), the donor solution (containing 1 μM of each compound, 1 μM Lucifer yellow and 0.2% (vol/vol) DMSO in the transport buffer) or the receiver solution (containing 0.2% (vol/vol) DMSO in the transport buffer) was added to each insert or each well of the newly prepared receiver plate. The reaction was initiated by putting the insert plate on the receiver plate and incubating in an incubator at 37 °C with 5% CO_2_ for 3 h. After the incubation, an aliquot of solution in each insert and well was withdrawn and mixed with 70% (vol/vol) acetonitrile including an internal standard (50 nM propranolol). The concentration in each compartment was quantified by means of LC–MS/MS. Paracellular flux was monitored by the appearance of Lucifer yellow in the opposite compartment.

### Brain-penetrability study in mice

Dosing solutions were prepared in 0.5% (wt/vol) hypromellose containing 0.1 M HCl. Compounds were administered orally to male BALB/c mice (CLEA Japan) at a dose of 50 mg per kg using a syringe with an oral catheter, and blood and brain were sampled 0.5 h or 1 h after the dose. Unbound fractions in plasma and brain (fu,plasma and fu,brain, respectively) were obtained by the equilibrium dialysis method with mouse plasma and mouse brain homogenate at 10 μM for each compound. Plasma was isolated from blood by centrifugation. The whole brain was immediately removed, rinsed with saline and promptly frozen in liquid nitrogen in polypropylene tubes and then stored in an ultra-low-temperature freezer until processing. Each brain sample was weighed and homogenized with three volumes of water. The concentration of compounds in each sample was quantified by LC–MS/MS.

### Protein-binding study

The in vitro unbound fraction of each compound in the plasma and brain homogenate of BALB/c mice was determined using a 96-well micro-equilibrium dialysis device (HTD 96b, Dialysis Membrane Strip, MWCO 12–14 kDa, HT Dialysis). Blank brain samples were homogenized in three volumes of PBS. Plasma or brain homogenate was spiked with each compound to achieve a final concentration of 10 μM. An aliquot of plasma or brain homogenate containing each compound was added in the donor side of a dialysis device. An aliquot of PBS was added in the reservoir side of the same device. The plate containing plasma or brain homogenate and buffer was equilibrated at 37 °C for 6 h in an incubator with 10% CO_2_ and constant shaking. After the incubation, samples were collected from the respective sides and mixed with PBS or blank plasma and ethanol including internal standard (100 nM labetalol). All samples were filtered, and the resultant filtrates were analyzed by LC–MS/MS to calculate the peak area ratio in the donor and reservoir sides.

### In-cell western assay

RET autophosphorylation was examined with Jump-In GripTite HEK293 cells transiently expressing WT or mutant KIF5B–RET. Cells were then treated with various concentrations of each test drug for 1 h, fixed in formalin and permeabilized with a mixture of 10% Triton X-100 (Nacalai Tesque). Fixed samples were blocked in Intercept Blocking Buffer (LI-COR) and incubated with anti-phospho-RET (Y905) (3221, Cell Signaling Technology) and anti-RET (sc-101422, Santa Cruz Biotechnology) antibodies (in blocking buffer) overnight in a cold room, and then IRDye 800CW goat anti-rabbit IgG and IRDye 680RD goat anti-mouse IgG (LI-COR) were added. Fluorescence signals of RET expression (700 nm) and phospho-RET expression (800 nm) were acquired by the total fluorescence intensity obtained by measuring the wavelengths of 700 nm and 800 nm with the Odyssey CLx imager (LI-COR). The total fluorescence intensity ratio of phospho-RET/RET in each well was calculated by dividing the total fluorescence intensity of phospho-RET (800 nm) in each well by the total fluorescence intensity of RET (700 nm). IC_50_ values (the concentration that exerted 50% autophosphorylation-inhibitory activity compared with that of the untreated controls) were calculated as a sigmoidal dose–response model in XLfit software (ID Business Solutions). Data are presented as mean ± s.d. of three independent experiments.

### Crystallography

Protein crystallography was performed by Proteros Biostructures. The kinase domain of human RET (residues S705 to R1012) was expressed in SF9 cells and purified by affinity chromatography and gel filtration, yielding >95% purity based on Coomassie-stained SDS–PAGE. The purified protein was concentrated to 6 mg ml^−1^ and used for crystallization studies. RET crystals with the ligands TAS-C1, selpercatinib and pralsetinib were obtained at 20 °C by sitting-drop vapor diffusion against 0.2 M lithium chloride, 2.5–3 M sodium formate, 5 mM magnesium chloride and 0.1 M sodium acetate buffer (pH 4.5–5.0). X-ray diffraction data were collected at the Swiss Light Source under cryogenic conditions at final resolutions of 1.64 Å, 2.75 Å and 2.31 Å respectively. The crystals belong to space group *P*2_1_. Data were processed using the programs XDS and XSCALE (TAS-C1 (PDB 7DUA), selpercatinib (PDB 7DU8), pralsetinib (PDB 7DU9)). Crystallographic data and refinement statistics are described in Supplementary Tables 5 and 6.

### Statistics and reproducibility

For animal studies, AUC analysis was used to compare the average tumor volume between groups. AUC and standard error were computed using the trapezoid method. The degrees of freedom (*n* value plotted) were defined as the number of data points for that group minus the number of separate time point measurements^60^. Negative AUC values indicate tumor regression. One-way ANOVA with Tukey’s multiple-comparison tests was employed to compare groups. When end-point tumor volumes were compared, statistical significance was calculated using Dunnett’s test. Two-way ANOVA with Tukey’s multiple-comparison test was used to compare treatment groups in caspase 3 and 7 studies. IC_50_ values were compared using 95% confidence interval values. GraphPad Prism 9 software, Microsoft Excel with the EXSUS System, XDS, XSCALE and XLfit 5 add-ins, ChemDraw version 19 and MOE202 were used to analyze and graph data. *P* < 0.05 was considered statistically significant, and all tests were two tailed. No statistical method was used to predetermine sample size. In survival analysis of NIH-3T3 intracranial xenograft data, three mice were excluded from survival analysis in the vepafestinib group due to accidental death. Animals were randomized to treatment groups in efficacy studies based on initial tumor volume and weight. No other randomization was used. The investigators were not blinded to allocation during experiments and outcome assessment. Data distribution was assumed to be normal, but this was not formally tested. Data collection and analysis were not performed blind to the conditions of the experiments. Measurements were taken from distinct samples, except for efficacy studies, in which tumors were measured repeatedly at different times.

### Reporting summary

Further information on research design is available in the Nature Portfolio Reporting Summary linked to this article.

### Supplementary information

Supplementary Information Supplementary Methods.

Reporting Summary

Supplementary Table 1 This file contains supplementary data referenced in the paper.

Supplementary Data 1 Full X-ray structure validation report for RET in complex with TAS-C1.

Supplementary Data 2 Full X-ray structure validation report for RET in complex with selpercatinib.

Supplementary Data 3 Full X-ray structure validation report for RET in complex with pralsetinib.

### Source data

Source Data Figs. 3, 4 and 7 and Extended Data Fig. 4 This file contains unprocessed images of immunoblots used in Figs. 3, 4 and 7 and Extended Data Fig. 4.

Source Data Fig. 1 This file contains statistical source data used to generate Fig. 1c.

Source Data Fig. 3 This file contains statistical source data used to generate Fig. 3b,c.

Source Data Fig. 4 This file contains statistical source data used to generate Fig. 4b.

Source Data Fig. 5 This file contains statistical source data used to generate Fig. 5.

Source Data Fig. 6 This file contains statistical source data used to generate Fig. 6.

Source Data Fig. 7 This file contains statistical source data used to generate Fig. 7a–d.

Source Data Fig. 8 This file contains statistical source data used to generate Fig. 8b–e.

Source Data Extended Data Fig. 5 This file contains statistical source data used to generate Extended Data Fig. 5c.

Source Data Extended Data Fig. 6 This file contains statistical source data used to generate Extended Data Fig. 6.

Source Data Extended Data Fig. 7 This file contains statistical source data used to generate Extended Data Fig. 7.

Source Data Extended Data Fig. 8 This file contains statistical source data used to generate Extended Data Fig. 8.

Source Data Extended Data Fig. 9 This file contains statistical source data used to generate Extended Data Fig 9.

Source Data Extended Data Fig. 10 This file contains statistical source data used to generate Extended Data Fig 10.

## Data Availability

X-ray crystal structures are available at the RCSB Protein Data Bank (https://www.rcsb.org) as PDB 7DUA, PDB 7DU8 and PDB 7DU9. All other data supporting the findings of this study are available from the corresponding author on reasonable request. Source data are provided with this paper.
