# Author Correction: Vepafestinib is a pharmacologically advanced RET-selective inhibitor with high CNS penetration and inhibitory activity against RET solvent front mutations

**DOI:** 10.1038/s43018-023-00663-3

**Published:** 2023-10-09

**Authors:** Isao Miyazaki, Igor Odintsov, Keiji Ishida, Allan J. W. Lui, Masanori Kato, Tatsuya Suzuki, Tom Zhang, Kentaro Wakayama, Renate I. Kurth, Ryan Cheng, Hidenori Fujita, Lukas Delasos, Morana Vojnic, Inna Khodos, Yukari Yamada, Kota Ishizawa, Marissa S. Mattar, Kaoru Funabashi, Qing Chang, Shuichi Ohkubo, Wakako Yano, Ryuichiro Terada, Claudio Giuliano, Yue Christine Lu, Annalisa Bonifacio, Siddharth Kunte, Monika A. Davare, Emily H. Cheng, Elisa de Stanchina, Emanuela Lovati, Yoshikazu Iwasawa, Marc Ladanyi, Romel Somwar

**Affiliations:** 1 Taiho Pharmaceutical Co. Ltd., Tsukuba, Japan; 2 Department of Pathology and Laboratory Medicine, Memorial Sloan Kettering Cancer Center, New York, NY USA; 3 Human Oncology and Pathogenesis Program, Memorial Sloan Kettering Cancer Center, New York, NY USA; 4 Antitumor Assessment Core Facility, Molecular Pharmacology Program, Memorial Sloan Kettering Cancer Center, New York, NY USA; 5 Helsinn Healthcare SA, Lugano, Switzerland; 6 Department of Pediatrics, Oregon Health Sciences University, Portland, OR USA; 7 Present Address: Department of Pathology, Brigham and Women’s Hospital, Harvard Medical School, Boston, MA USA; 8 Present Address: Cancer Research UK Cambridge Institute, University of Cambridge, Cambridge, UK; 9 Present Address: Department of Hematology and Medical Oncology, Cleveland Clinic Taussig Cancer Institute, Cleveland, OH USA; 10 Present Address: Northwell Health Cancer Institute, Lenox Hill Hospital, New York, NY USA; 11 Present Address: Department of Education and Support for Regional Medicine, Tohoku University Hospital, Sendai, Japan; 12 Present Address: Dana Cancer Center, Toledo, OH USA

**Keywords:** Targeted therapies, Structure-based drug design, Cancer, Kinases, Mechanisms of disease

Correction to: *Nature Cancer* 10.1038/s43018-023-00630-y, published online 21 September 2023.

In the version of the article initially published, the affiliation of Ryan Cheng was incorrect and has now been updated to the Department of Pathology and Laboratory Medicine, Memorial Sloan Kettering Cancer Center, New York, NY, USA, in the HTML and PDF versions of the article.

