# Supplementary material for: Vepafestinib is a pharmacologically advanced RET-selective inhibitor with high CNS penetration and inhibitory activity against RET solvent front mutations
Source: Nat Cancer. 2023 Sep 21;4(9):1345–61. doi: 10.1038/s43018-023-00630-y (PMC10518257; doi:10.1038/s43018-023-00630-y)
Supplement: Supplementary file 1 — Supplementary Methods. [file 43018_2023_630_MOESM1_ESM.pdf]

nature cancer

Article

https://doi.org/10.1038/s43018-023-00630-y

Vepafestinib is a pharmacologically advanced RET-selective inhibitor with high CNS penetration and inhibitory activity against RET solvent front mutations

In the format provided by the authors and unedited

Supplementary Data Materials and Method

Vector construction. Expression vectors containing *KIF5B::RET* variants were constructed by site-specific mutagenesis utilizing In-Fusion cloning (Takara Bio Inc., Tokyo, Japan). Briefly, PCR primers were designed with overlapping 15-mer bases which incorporated the mutations of interest. PCR reactions were conducted with PrimeStar Max reagent (Takara Bio Inc., Japan) using forward and reverse primer sets targeting the front or the rear of the fusion, and mutation-containing primers. The appropriate pair of each PCR product was fused with In-Fusion master mix at 50°C for 15 min. After cloning and purification, mutated *KIF5B::RET* were transferred into pJTI™ Fast-based expression vector using Gateway recombination system (ThermoFisher Scientific Inc., Waltham, MA).

Growth inhibition assay in Ba/F3 cells. BaF3 cells were cultured in 96-well plates (1,000 per well) and exposed to inhibitors t for 72 hours at 37°C. Cell viability was assessed by luminescence using CellTiter-Glo 2.0 Assay (Promega, Madison, WI)). GI_{50} values (the concentration that exerted 50% growth inhibition compared with vehicle-treated controls) were calculated using a sigmoidal dose response model in XLfit software (ID Business Solutions, Woking, UK) or using inverse estimation of regression and linear regression analysis using SAS ver. 9.2 via EXSUS ver.8.0.0. (CAC Exicare Corporation (Tokyo, Japan). Data represent the mean ± SD of three independent experiments.

RET WT kinase inhibition assay. Recombinant human RET was purchased from Carna Biosciences, Inc (Kobe, Japan). IC_{50} determination was performed using LANCE® time-resolved fluorescence resonance energy transfer (TR-FRET) assay technology. Briefly, 0.003 µg/mL RET and 250 nM peptide substrate (biotin-EEPLYWSFPAKKK-NH_2) were mixed, then incubated for 100 min at 25°C in a reaction mixture containing 10 µM ATP, 10 mM $MgCl_2$, 2 mM DTT, 15 mM Tris (pH 7.5), 0.01% Tween 20 or 13.5 mM Tris (pH 7.5), 0.009% Tween 20, and ten concentrations of test compounds. The reaction was stopped by addition of EDTA, and then a mixture containing LANCE® Eu-W-1024 labeled anti-phosphotyrosine PT66 antibody (PerkinElmer, Waltman, MA) and Streptavidin conjugated to SureLight®-Allophycocyanin for kinase assays (PerkinElmer, Waltman, MA) were added to facilitate detection. After incubation for 120 min at room temperature, fluorescence emission at 620 nm and 665 nm was measured after excitation at 337 nm with PHERAstarFS (BMG LABTECH GmbH, Offenburg, Germany). The TR-FRET signal of each sample was calculated from the emission ratio of 665 nm:620 nm × 10,000of. IC_{50} values were calculated using a sigmoidal dose response model with the TR-FRET signals of positive (no inhibitor) and negative (EDTA was added before the reaction start) control wells.

Kinase selectivity assay. The inhibitory effects of vepafestinib (TAS0953/HM06, 23 nM), selpercatinib (22 nM), pralsetinib (17 nM) and TPX-0046 (26 nM) on the enzymatic activity of a panel of 255 (vepafestinib) or 256 (all other compounds) recombinant kinases were performed by Carna Biosciences, Inc. (Kobe, Japan), according to their procedure. Reaction conditions for each kinase were optimized for time, substrate, ATP and metal concentrations. The readout value of reaction control (complete reaction mixture) was set as 0% inhibition, and the readout value of background (reaction mixture without enzyme) was set as 100% inhibition and percent inhibition of each test solution was calculated accordingly. For determination of inhibitory capacity of each agent on the 14 "off-target" kinases, protein kinase assays were performed in the presence of increasing concentrations of RET inhibitor. IC_{50} values were calculated from concentration vs. % inhibition curves by fitting to a four parameter logistic equation.

Pharmacokinetic analysis of vepafestinib in rats. Twelve adult male Han® Wistar rats (283-345 g; Charles River Laboratories, Sulzfeld, Germany) were used for the experiments**.** Each animal was placed in a stereotaxic frame (Kopf Instruments, Tujunga, CA, USA). Guides for MetaQuant microdialysis probes with a 4-mm exposed regenerated cellulose membrane (MQ-RC 3/4; Charles River Laboratories, Den Bosch, The Netherlands) were implanted bilaterally into the prefrontal cortex one day prior to microdialysis sampling. In the same surgical procedure, an indwelling cannula was inserted into the cisterna magna to accommodate CSF sampling and an indwelling cannula was inserted into the jugular vein. Vepafestinib was formulated in 5% (w/v) glucose in ultra-purified water at a concentration of 0.6, 2 or 10 mg/mL for oral dosing at 3, 10 or 50 mg/kg free base (5 mL/kg), respectively. *In vivo* experiments were initiated one day after surgery. The MetaQuant microdialysis probes were connected with flexible PEEK tubing (Western Analytical Products Inc., Lake Elsinore, CA,USA; PK005-020) to a microperfusion pump (Harvard Apparatus, Holliston, MA) and perfused with a slow flow of a CSF + 0.2% (w/v) BSA (perfusate) at a flow rate of 0.12 µL/min and a carrier flow of ultra-purified water at a flow rate of 0.8 µL/min. After a minimum of two hours of prestabilization, microdialysis samples were collected in 30-minute intervals. Following collection of one baseline sample, vepafestinib was administered orally at t = 0 minutes. Microdialysate samples were collected for an additional 270 minutes after compound administration on day 1, and from t = 23.5-25.5h on day 2. Samples were collected into polystyrene microvials (Microbiotech/se AB, Stockholm, Sweden; 4001029) using an automated fraction collector (UV 8301501, TSE, Univentor, Malta). All samples were stored at -80 °C until off-line analysis. CSF samples were taken from the cisterna magna through the cannula and collected into polystyrene microvials and stored at -80 °C until analysis. Blood samples were drawn via the jugular vein cannula, and collected into vials containing K_2EDTA, for plasma generation. The resultant plasma samples were also stored in polystyrene

microvials at -80 °C until off-line analysis. At the end of the experiment, animals were sacrificed and terminal brain tissue was collected for histological verification of the probe position. Concentrations of vepafestinib in MetaQuant microdialysate, CSF and plasma samples were determined by HPLC with tandem mass spectrometry (MS/MS) detection using D_8-vepafestinib as the internal standard.

***In vivo* efficacy Studies.** All animal experiments (Ba/F3 allografts and LC-2/ad xenografts) were performed with the approval of the institutional animal care and use committee of Taiho Pharmaceutical Co., Ltd. and carried out according to the guidelines for animal use and care of Taiho Pharmaceutical Co., Ltd. The cells were implanted into male BALB/c nude mice (CLEA Japan, Inc.) and allowed to grow to approximately 100 mm^3. Five or six animals were assigned to each group per experiment. Vepafestinib, selpercatinib and pralsetinib were suspended in 0.5% hypromellose (HPMC) with 0.1 M HCl solution, respectively. Inhibitor suspension was administered orally twice daily for 14 days. Tumor volume was calculated with the following formula: $[\text{length} \times (\text{width})^2]/2$. Statistical significance was calculated by using Dunnett's test to assess the difference in tumor volumes between the control (vehicle-treated) and treatment groups. $P < 0.05$ was considered statistically significant. In addition, animal weight was monitored during the dosing period. The body weight of each animal was measured twice weekly (**Supplementary Figs. 8 and 9**).For pharmacodynamic analysis, tumors were harvested after administration of compounds. For evaluation of the phosphorylation status of proteins, the excised tumors were homogenized in cellular protein extraction buffer.

Intracranial efficacy study. NIH-3T3-CCDC6::RET-Luc cells (2.5×10^4 cells/mouse) were injected into the brain of athymic nude mice (CLEA Japan, Inc.) at a depth of 3.5 mm at a position 0.5-mm anterior and 2-mm right lateral to the bregma. Four days after implantation, mice were intraperitoneally injected with luciferin (FUJIFILM Wako Pure Chemical Corporation) and luminescence of the mice was measured with an In Vivo Imaging System (Lumina II, PerkinElmer). The total flux (photons/s) in the region of interest of the head of the mouse was quantified using Living Image software (PerkinElmer, Waltham, MA). Mice were randomly allocated into two groups of ten to equalize the mean total photons in each group, and then orally administered vehicle or 50 mg/kg vepafestinib (TAS0953/HM06) twice daily (BID) from day 5. Luminescence signals were measured at day 13. Effect of vepafestinib on the survival time was evaluated by examining the difference in survival curves between the vehicle group and the vepafestinib-treated group of animals by using the two-sided log-rank test. $P<0.05$ was considered statistically significant. Three mice in the vepafestinib group were excluded from the survival curve due to accidental death.

Transcellular transport and brain penetrability study in mice. Transcellular transport, brain penetrability and protein binding study for TPX-0046 were conducted by using the same materials and experimental procedure as those for other compounds with minor modification as follows. In transcellular transport study, Dextran Texas Red (3000 MW, Neutral; Thermo Fisher Scientific) was used as an impermeable marker. In protein binding study, a dialysis device (HTDialysis) containing plasma or brain homogenate spiked with TPX-0046 (10 μmol/L as final concentration) and PBS was equilibrated at 37°C for 6 h in 5% CO_2 incubator with constant shaking.

Model building and system setup for the docking simulations. The co-crystal structure of wild-type RET with TAS compound 1 was used for the structural modeling of the five solvent-front mutants of RET (G810A, G810C, G810D, G810R, G810S). The conformations of the mutated residues were explored by the rotamer method implemented in Molecular Operating Environment 2020 (MOE2020) software package[1]. In order to investigate the binding modes of vepafestinib to wild-type and mutated RET the 2D and 3D structures of vepafestinib were generated using ChemDraw and Molecular Operating Enviroment (MOE) suites, respectively. To the ligand and six receptors, the hydrogen atoms were added by the protonate 3D module with the default parameter set, and thereafter minimized in MOE. For the energy minimization and following docking simulations, AMBER14;EHT force field was applied to all atoms with GB/VI solvation model[2].

Docking Simulation. MOE-ASEDock[3] was employed to perform the docking simulations. The parameter for the generation of ligand conformations was set to 25,000. The stochastic search algorithm was used. Ligand binding sites were explored by using alpha site finder in MOE. In all six receptors, the most plausible ligand binding site was found in the ATP binding pocket, though the solvent-front residues differ (i.e., G810, A810, C810, D810, R810, and S810).

Supplementary Data References

1. Molecular Operative Environment (MOE), 2020.09 Chemical Computing Group ULC, 1010 Sherbrooke St. West, Suite #910, Montreal, QC, Canada, H3A 2R7, 2020.
2. Labute, P. The generalized Born/volume integral implicit solvent model: estimation of the free energy of hydration using London dispersion instead of atomic surface area, *J Comput Chem.* **29**, 1693-8 (2008).
3. Goto, J., *et al*. ASEDock-docking based on alpha spheres and excluded volumes, *J Chem Inf Model.* **48**, 583-90 (2008).
