# Supplementary material for: Vepafestinib is a pharmacologically advanced RET-selective inhibitor with high CNS penetration and inhibitory activity against RET solvent front mutations
Source: Nat Cancer. 2023 Sep 21;4(9):1345–61. doi: 10.1038/s43018-023-00630-y (PMC10518257; doi:10.1038/s43018-023-00630-y)
Supplement: Supplementary file 2 — Reporting Summary [file 43018_2023_630_MOESM2_ESM.pdf]

Corresponding author(s): Romel Somwar, PhD

Last updated by author(s): 2023.08.06

Reporting Summary

Nature Portfolio wishes to improve the reproducibility of the work that we publish. This form provides structure for consistency and transparency in reporting. For further information on Nature Portfolio policies, see our Editorial Policies and the Editorial Policy Checklist.

Statistics

For all statistical analyses, confirm that the following items are present in the figure legend, table legend, main text, or Methods section.

n/a	Confirmed	
☐	☒	The exact sample size (*n*) for each experimental group/condition, given as a discrete number and unit of measurement
☐	☒	A statement on whether measurements were taken from distinct samples or whether the same sample was measured repeatedly
☐	☒	The statistical test(s) used AND whether they are one- or two-sided<br>*Only common tests should be described solely by name; describe more complex techniques in the Methods section.*
☒	☐	A description of all covariates tested
☐	☒	A description of any assumptions or corrections, such as tests of normality and adjustment for multiple comparisons
☐	☒	A full description of the statistical parameters including central tendency (e.g. means) or other basic estimates (e.g. regression coefficient) AND variation (e.g. standard deviation) or associated estimates of uncertainty (e.g. confidence intervals)
☒	☐	For null hypothesis testing, the test statistic (e.g. *F*, *t*, *r*) with confidence intervals, effect sizes, degrees of freedom and *P* value noted<br>*Give P values as exact values whenever suitable.*
☒	☐	For Bayesian analysis, information on the choice of priors and Markov chain Monte Carlo settings
☒	☐	For hierarchical and complex designs, identification of the appropriate level for tests and full reporting of outcomes
☒	☐	Estimates of effect sizes (e.g. Cohen's *d*, Pearson's *r*), indicating how they were calculated

Our web collection on statistics for biologists contains articles on many of the points above.

Software and code

Policy information about availability of computer code

Data collection	Microsoft Excel 2018, Molecular Operating Environment 2020 (MOE2020),
Data analysis	Microsoft Excel with EXUS System, SDS and XSCALE, XLfit 5 add-in for Excel, Graphpad Prism v9, Chemdraw v19, MOE2020,

For manuscripts utilizing custom algorithms or software that are central to the research but not yet described in published literature, software must be made available to editors and reviewers. We strongly encourage code deposition in a community repository (e.g. GitHub). See the Nature Portfolio guidelines for submitting code & software for further information.

Data

Policy information about availability of data

All manuscripts must include a data availability statement. This statement should provide the following information, where applicable:

- Accession codes, unique identifiers, or web links for publicly available datasets
- A description of any restrictions on data availability
- For clinical datasets or third party data, please ensure that the statement adheres to our policy

All data used to generate graphs and immunoblot images are available as Source Data files. X-ray crystal structures are available on the protein data bank website (rcsb.org) as PDB files 7DUA, 7DU8 and 7DU9

Research involving human participants, their data, or biological material

Policy information about studies with human participants or human data. See also policy information about sex, gender (identity/presentation), and sexual orientation and race, ethnicity and racism.

Reporting on sex and gender	*Use the terms sex (biological attribute) and gender (shaped by social and cultural circumstances) carefully in order to avoid confusing both terms. Indicate if findings apply to only one sex or gender; describe whether sex and gender were considered in study design; whether sex and/or gender was determined based on self-reporting or assigned and methods used. Provide in the source data disaggregated sex and gender data, where this information has been collected, and if consent has been obtained for sharing of individual-level data; provide overall numbers in this Reporting Summary. Please state if this information has not been collected. Report sex- and gender-based analyses where performed, justify reasons for lack of sex- and gender-based analysis.*
Reporting on race, ethnicity, or other socially relevant groupings	*Please specify the socially constructed or socially relevant categorization variable(s) used in your manuscript and explain why they were used. Please note that such variables should not be used as proxies for other socially constructed/relevant variables (for example, race or ethnicity should not be used as a proxy for socioeconomic status). Provide clear definitions of the relevant terms used, how they were provided (by the participants/respondents, the researchers, or third parties), and the method(s) used to classify people into the different categories (e.g. self-report, census or administrative data, social media data, etc.) Please provide details about how you controlled for confounding variables in your analyses.*
Population characteristics	*Describe the covariate-relevant population characteristics of the human research participants (e.g. age, genotypic information, past and current diagnosis and treatment categories). If you filled out the behavioural & social sciences study design questions and have nothing to add here, write "See above."*
Recruitment	*Describe how participants were recruited. Outline any potential self-selection bias or other biases that may be present and how these are likely to impact results.*
Ethics oversight	*Identify the organization(s) that approved the study protocol.*

Note that full information on the approval of the study protocol must also be provided in the manuscript.

Field-specific reporting

Please select the one below that is the best fit for your research. If you are not sure, read the appropriate sections before making your selection.

☒ Life sciences ☐ Behavioural & social sciences ☐ Ecological, evolutionary & environmental sciences

For a reference copy of the document with all sections, see nature.com/documents/nr-reporting-summary-flat.pdf

Life sciences study design

All studies must disclose on these points even when the disclosure is negative.

Sample size	No sample-size calculation was performed. Sample size was based on historical research experience and published work (references 45, 54).
Data exclusions	In survival analysis of NIH-3T3 intracranial xenograft data three mice were excluded from survival analysis in the vepafestinib group due to accidental death.
Replication	Each experiment had three replicates of each condition and the results were always consistent between replicates of a given condition. The majority of experiments were conducted more than two to five times, using different passages of cells and different drug stocks, and by different personnel in some instances. Results were consistent from experiment to experiment as evidence by error bars and confidence intervals.
Randomization	For animal studies, tumor bearing mice were assigned to cages when tumor volumes were approximately 100 mm3 so that the average tumor volume of all cages were the similar. Cages were then randomly assigned to treatment groups. No randomization was used for other studies.
Blinding	No systematic blinding was used. However, the personnel conducting animal studies were not aware of the nature of any inhibitors nor expected outcomes, and were not involved in data analysis. Replicate experiments were also conducted by different authors on the manuscript where possible.

Reporting for specific materials, systems and methods

We require information from authors about some types of materials, experimental systems and methods used in many studies. Here, indicate whether each material, system or method listed is relevant to your study. If you are not sure if a list item applies to your research, read the appropriate section before selecting a response.

Materials & experimental systems

n/a	Involved in the study	
☐	☒	Antibodies
☐	☒	Eukaryotic cell lines
☐	☐	Palaeontology and archaeology
☐	☒	Animals and other organisms
☒	☐	Clinical data
☒	☐	Dual use research of concern
☒	☐	Plants

Methods

n/a	Involved in the study	
☒	☐	ChIP-seq
☒	☐	Flow cytometry
☒	☐	MRI-based neuroimaging

Antibodies

Antibodies used

Antibodies used in this study is listed below (with supplier, catalog number, clone and dilution used) and and also included in Supplementary Table 4.
P-RET Y1062 R&D Systems, polyclonal (Cat. number AF5009) 1:500
P-RET Y905 Cell Signaling Technology, polyclonal (Cat. number 3221) "1:500
1:1000 (Ba/F3)"
RET Cell Signaling Technology, C31B4 1:1000
P-ERK1/2 T202/204 Cell Signaling Technology, D13.14.4E "1:2000
1:1000 (Ba/F3)"
ERK1/2 Cell Signaling Technology, 137F5 1:2000 for human cells and 1:1000 for Ba/F3 cells
GAPDH Cell Signaling Technology, D16H11 1:10000 for human cells and 1:1000 for Ba/F3
P-AKT1/2/3 S473 Cell Signaling Technology, D39 1:1000
AKT1/2/3 Cell Signaling Technology, 11E7 1:1000
P-P70S6K T421/S424 Cell Signaling Technology, polyclonal (Cat. number 9204) 1:1000
P70S6K Cell Signaling Technology, 49D7 1:2000
P-S6RP S235/236 Cell Signaling Technology, D57.2.2E 1:1000
S6RP Cell Signaling Technology, 5G10 1:2000
Cyclin D1 Cell Signaling Technology, 92G2 1:1000
P21 Cell Signaling Technology, 12D1 1:500
P27 Cell Signaling Technology, D69C12 1:500
Cleaved PARP Asp214 Cell Signaling Technology, D64E10 1:1000
BIM Cell Signaling Technology, C34C5 1:500
PUMA Cell Signaling Technology, polyclonal (catalog # 4976) 1:500
Vinculin Cell Signaling Technology, E1E9V 1:2000

Anti-Rabbit IgG, R and D Systems ((Cat. number HAF008) 1:5000
Anti-Mouse IgG, R and D Systems ((Cat. number HAF018) 1:2500

Validation

All antibodies used in this study were obtained from commercial sources. The validation information provided below are from the Websites of the vendors.

P-RET Y1062 validated for Western blotting against TT cell extracts. Recognizes human.

P-RET Y905 validated for Western blotting against GST-RET and TT cell extracts. Recognize human and Drosophila melanogaster

RET validated for Western blotting against TT cell extracts. Recognize human and mouse. Immunofluorescence analysis validated for MCF7 and HeLa cells.

P-ERK1/2 (T202/Y204) validated for Western blotting against COS, 293, NIH/3T3, and C6 cell extracts. Recognizes human, mouse, rat, hamster, monkey, mink, D. melanogaster, zebrafish, bovine, dog, pig, and S. cerevisiae. IHC analysis validated for human breast carcinoma, human lung carcinoma, and NIH/3T3 samples. Immunofluorescence analysis validated for drosophila egg chambers, and HT1080 cells.

ERK1/2 validated for Western blotting against HeLa, NIH/3T3, C6, HEK 293, and Jurkat cell extracts. Recognizes human, mouse, rat, hamster, monkey, mink, D. melanogaster, zebrafish, bovine, dog, pig, and S. elegans. IHC analysis validated for human breast carcinoma, and human colon carcinoma samples. Immunofluorescenceanalysis validated for NIH/3T3 cells.

GAPDH validated for Western blotting against HeLa, NIH/3T3, C6, and COS-7 cell extracts. Recognizes human, mouse, rat, and monkey. IHC analysis validated for human breast carcinoma and mouse colon samples. Immunofluorescence analysis validated for C2C12 cells.

P-AKT1/2/3 S473 validated for Western blotting against PC-3, NIH/3T3, and Jurkat cell extracts. Recognizes human, mouse, rat, hamster, monkey, D. melanogaster, zebrafish, and bovine. Predicted to recognize chicken, xenopus, dog and pig. Also validated for IHC analysis of MDA-MB-468 xenograft, human breast carcinoma, human lung carcinoma, mouse endometrial, U-87MG xenograft, and LNCaP samples. validated for immunodluorescence analysis of C2C12 cells.

AKT1/2/3 validated for Western blotting against HeLa, NIH/3T3, C6, and COS cell extracts. Validated for IHC analysis of human melanoma, human breast carcinoma and LNCaP samples. Validated for immunofluorescence analysis of C2C12 cells. Recognizes human, mouse, rat, monkey, and D. melanogaster. Predicted to recognize pig.

P-P70S6K T421/S424 validated for Western blotting against 293 cell extract. Recognizes human, mouse, rat, and monkey.

P70S6K validated for Western blotting against MCF7, HeLa, HT-29, and K-562 cell extracts. Recognizes human.

P-S6RP S235/236 validated for Western blotting against MCF7 cell extract. IHC analysis validated for LNCaP, human breast carcinoma, human colon carcinoma, human lung carcinoma, mouse spleen, and A549 xenograft samples. Immunofluorescence analysis validated in HeLa cell samples. Recognizes human, mouse, rat, monkey, mink, and S. cerevisiae.

SS6RP validated for Western blotting against HeLa, H4IIE, 3T3, and COS cell extract. Validated for IHC analysis of human breast carcinoma, human colon carcinoma, LNCaP, and human lung carcinoma samples. Validated for immunofluorescence analysis of HeLa cells. Recognizes human, mouse, rat, monkey, and D. melanogaster.

Vinculin validated for Western blotting against Hs578T, HeLa, Jurkat, PC-12, and COS-7 cell extracts. IHC analysis validated for human colon carcinoma, human endometroid adenocarcinoma, human breast ductal carcinoma in situ, human non-small cell lung carcinoma, mouse spleen, Hs578T and Jurkat samples. Recognizes human, mouse, rat, and monkey.
PUMA validated for Western blotting against L-540, GRANTA 519, K-562, KMS-11, LoVo, PC-3, BA/F3, A20, and HCT 116 cell extract. Immunofluorescent analysis validated for HCT 116 cell samples. Recognizes human and mouse.

BIM validated for Western blotting against Raji, A20, RL, and HeLa cell extracts. IHC analysis validated for human colon adenocarcinoma, human non-small cell lung carcinoma, human lung carcinoma, human lymphoma, human breast carcinoma, and 4T1 syneneic tumor samples. Immunofluorescence analysis validated for MCF-7 cells. Recognizes human, mouse, rand rat.
Cleaved PARP Asp214 validated for Western blotting against HeLa and Jurkat cell extracts. IHC analysis validated for human tonsil samples. Immunofluorescent analysis validated for HeLa cells. Recognizes human and monkey.
p27 validated for Western blotting against HeLa, 293, COS, C6, and MCF-7 cell extracts. Validated for immunoflorescence analysis of MCF-7 cells. Recognizes human, rat and monkey.

p21 validated for Western blotting against HeLa, HUVEC, COS, SH-SY5Y, and MCF-7 cell extracts. Validated for IHC analysis of human breast carcinoma, and HeLa cell samples. Validated for mmunofluorescence analysis of MCF7 cells. Recognizes human and monkey. Predicted to recognize dog.

Cyclin D1 validated for Western blotting against MCF7, L929, and C6 cell extracts. Validated for IHC analysis of human colon carcinoma, Apc mouse intestine, human breast carcinoma, and H1975 xenograft samples. Recognizes human, mouse, and rat.

Detects rabbit IgG heavy and light chains in direct ELISAs and Western blots. In direct ELISAs, less than 5% cross-reactivity with human IgG, mouse IgG, and chicken IgY is observed. Validated as secondary antibody against rabbit HSP60 polyclonal antibody with Jurkat, MCF-7, NIH-3T3, and Nb2-11 cell extracts.

Detects mouse IgG1, IgG2A, IgG2B, and IgG3 heavy and light chains in direct ELISAs and Western blots. In direct ELISAs, less than 2% cross-reactivity with human IgG and rabbit IgG is observed. Validated as secondary antibody against mouse STAT3 monoclonal antibody with HepG2, HeLa, NIH-3T3, and PC-12 cell extracts.

Eukaryotic cell lines

Policy information about cell lines and Sex and Gender in Research

Cell line source(s)	NIH-3T3 (CRL-1658) and TT (CRL-1803) cells: purchased from ATCC and catalog numbers are provided in brackets. Ba/F3 (RCB4476) and LC-2/ad (RCB0440) cells were obtained from RIKEN BioResource Center (Japan) and catalog numbers are provided in brackets. MMNK1 cells were purchased from JCRB Cell Bank (Catalog # JCRB1554, Japan) Jump-In GripTite HEK293 cells were purchased from ThermoFisher Scientific (catalog # A14150). MDR1-LLC-PK1 (catalog # 450211) and LLC-PK1 (catalog # 450216) cells were obtained from Discovery Labware. ECLC5B, LUAD-0002AS1: created by the authors at MSKCC. BCRP-MDCK II and MDCK II cells were obtained from the Netherlands Cancer Institute. HBEC cells were obtained from Dr. John Minna, UT Southwestern, Texas. Dr. Minna created the HBEC cells in his laboratory. All isogenic cell lines derived from the parental lines listed above were made by the authors.
Authentication	Human Cell lines were authenticated by the MSK-IMPACT platform , STR or by commercial suppliers. During the course of the study, the authors routinely checked cells by PCR for unique genomic markers such as fusion genes. The LLC-PK1 cells (porcine) and MDCK II cells (canine) were used within two months from verified stocks.
Mycoplasma contamination	Cells were tested for mycoplasma every 3-4 months. No cell line used in this study were ever contaminated with mycoplasma.
Commonly misidentified lines (See ICLAC register)	No commonly misidentified cells were used in this study.

Palaeontology and Archaeology

Specimen provenance	*Provide provenance information for specimens and describe permits that were obtained for the work (including the name of the issuing authority, the date of issue, and any identifying information). Permits should encompass collection and, where applicable,*

export.

Specimen deposition — *Indicate where the specimens have been deposited to permit free access by other researchers.*

Dating methods — *If new dates are provided, describe how they were obtained (e.g. collection, storage, sample pretreatment and measurement), where they were obtained (i.e. lab name), the calibration program and the protocol for quality assurance OR state that no new dates are provided.*

☐ Tick this box to confirm that the raw and calibrated dates are available in the paper or in Supplementary Information.

Ethics oversight — *Identify the organization(s) that approved or provided guidance on the study protocol, OR state that no ethical approval or guidance was required and explain why not.*

Note that full information on the approval of the study protocol must also be provided in the manuscript.

Animals and other research organisms

Policy information about studies involving animals; ARRIVE guidelines recommended for reporting animal research, and Sex and Gender in Research

Laboratory animals	Mouse strains used in this study: Balb/c, NOD scid gamma and athymic nude. Adult Han Wistar rats were also used in this study. Animals were approximately 6-8 weeks of age at the start of the respective experiment.
Wild animals	This study did not involve wild animals
Reporting on sex	There is no indication that RET fusions show any sex-specific distribution and there is no indication that response to RET inhibitors is altered by sex of the patient. Therefore, our study was not designed with sex as a variable.
Field-collected samples	This study does not involve samples collected in the field.
Ethics oversight	All animals were monitored daily and cared for in accordance with guidelines approved by the MSK Institutional Animal Care and Use Committee and Research Animal Resource Center or the Institutional Animal Care and Use Committee of Taiho Pharmaceutical Co., Ltd. The maximum allowed tumor size was 2 cm3. This size was not exceeded in any of our experiments.

Note that full information on the approval of the study protocol must also be provided in the manuscript.
