# Supplementary material for: Vepafestinib is a pharmacologically advanced RET-selective inhibitor with high CNS penetration and inhibitory activity against RET solvent front mutations
Source: Nat Cancer. 2023 Sep 21;4(9):1345–61. doi: 10.1038/s43018-023-00630-y (PMC10518257; doi:10.1038/s43018-023-00630-y)
Supplement: Supplementary file 4 — Full X-ray structure validation report for RET in complex with TAS-C1. [file 43018_2023_630_MOESM4_ESM.pdf]

Full wwPDB X-ray Structure Validation Report (i)

Jan 4, 2022 – 06:09 PM JST

PDB ID	:	7DUA
Title	:	Crystal structure of human Proto-oncogene tyrosine-protein kinase receptor Ret in complex with 4-amino-7-(1-methylcyclopropyl)-N-(5-methyl-1H-pyrazol-3-yl)pyrrolo[2,3-d]pyrimidine-5-carboxamide
Authors	:	Miyazaki, I.; Ishida, K.; Suzuki, T.
Deposited on	:	2021-01-08
Resolution	:	1.64 Å(reported)

This is a Full wwPDB X-ray Structure Validation Report for a publicly released PDB entry.

We welcome your comments at *validation@mail.wwpdb.org*
A user guide is available at
https://www.wwpdb.org/validation/2017/XrayValidationReportHelp
with specific help available everywhere you see the (i) symbol.

The following versions of software and data (see references (i)) were used in the production of this report:

MolProbity	:	4.02b-467
Mogul	:	1.8.5 (274361), CSD as541be (2020)
Xtriage (Phenix)	:	1.13
EDS	:	2.25
buster-report	:	1.1.7 (2018)
Percentile statistics	:	20191225.v01 (using entries in the PDB archive December 25th 2019)
Refmac	:	5.8.0158
CCP4	:	7.0.044 (Gargrove)
Ideal geometry (proteins)	:	Engh & Huber (2001)
Ideal geometry (DNA, RNA)	:	Parkinson et al. (1996)
Validation Pipeline (wwPDB-VP)	:	2.25

1 Overall quality at a glance (i)

The following experimental techniques were used to determine the structure:
X-RAY DIFFRACTION

The reported resolution of this entry is 1.64 Å.

Percentile scores (ranging between 0-100) for global validation metrics of the entry are shown in the following graphic. The table shows the number of entries on which the scores are based.

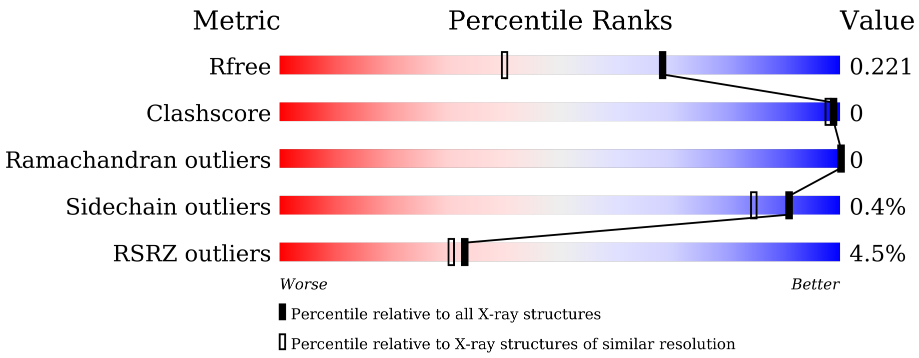

Metric	Whole archive (#Entries)	Similar resolution (#Entries, resolution range(Å))
R_{free}	130704	3122 (1.66-1.62)
Clashscore	141614	3268 (1.66-1.62)
Ramachandran outliers	138981	3215 (1.66-1.62)
Sidechain outliers	138945	3215 (1.66-1.62)
RSRZ outliers	127900	3079 (1.66-1.62)

The table below summarises the geometric issues observed across the polymeric chains and their fit to the electron density. The red, orange, yellow and green segments of the lower bar indicate the fraction of residues that contain outliers for >=3, 2, 1 and 0 types of geometric quality criteria respectively. A grey segment represents the fraction of residues that are not modelled. The numeric value for each fraction is indicated below the corresponding segment, with a dot representing fractions <=5% The upper red bar (where present) indicates the fraction of residues that have poor fit to the electron density. The numeric value is given above the bar.

Mol	Chain	Length	Quality of chain
1	A	314	2% / 88% • 9%
1	B	314	6% / 85% • 13%

2 Entry composition (i)

There are 3 unique types of molecules in this entry. The entry contains 4988 atoms, of which 0 are hydrogens and 0 are deuteriums.

In the tables below, the ZeroOcc column contains the number of atoms modelled with zero occupancy, the AltConf column contains the number of residues with at least one atom in alternate conformation and the Trace column contains the number of residues modelled with at most 2 atoms.

- Molecule 1 is a protein called Proto-oncogene tyrosine-protein kinase receptor Ret.

Mol	Chain	Residues	Atoms					ZeroOcc	AltConf	Trace
1	A	287	Total<br>2350	C<br>1506	N<br>408	O<br>422	S<br>14	49	5	0
1	B	274	Total<br>2223	C<br>1430	N<br>383	O<br>395	S<br>15	24	3	0

There are 10 discrepancies between the modelled and reference sequences:

Chain	Residue	Modelled	Actual	Comment	Reference
A	700	GLY	-	expression tag	UNP P07949
A	701	PRO	-	expression tag	UNP P07949
A	702	LEU	-	expression tag	UNP P07949
A	703	SER	-	expression tag	UNP P07949
A	704	LEU	-	expression tag	UNP P07949
B	700	GLY	-	expression tag	UNP P07949
B	701	PRO	-	expression tag	UNP P07949
B	702	LEU	-	expression tag	UNP P07949
B	703	SER	-	expression tag	UNP P07949
B	704	LEU	-	expression tag	UNP P07949

- Molecule 2 is 4-azanyl-7-(1-methylcyclopropyl)- {N}-(5-methyl-1 {H}-pyrazol-3-yl)pyrrolo[2,3-d]pyrimidine-5-carboxamide (three-letter code: HJ0) (formula: $C_{15}H_{17}N_7O$) (labeled as "Ligand of Interest" by depositor).

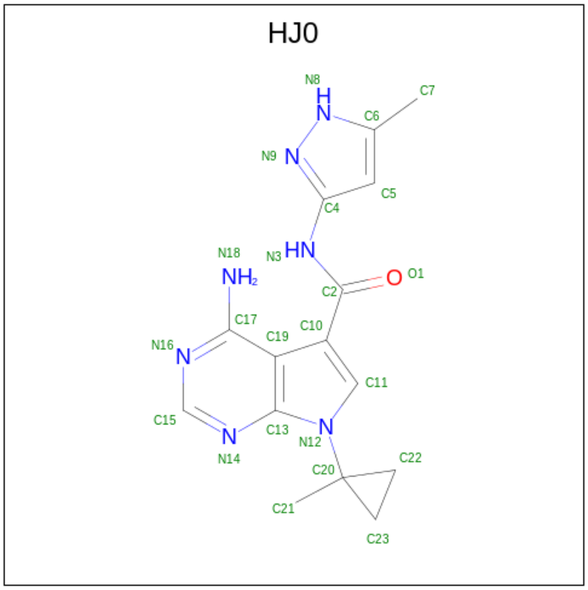

Mol	Chain	Residues	Atoms	ZeroOcc	AltConf
2	A	1	Total C N O<br>23 15 7 1	0	0
2	B	1	Total C N O<br>23 15 7 1	0	0

- Molecule 3 is water.

Mol	Chain	Residues	Atoms	ZeroOcc	AltConf
3	A	212	Total O<br>212 212	0	0
3	B	157	Total O<br>157 157	0	0

3 Residue-property plots ⓘ

These plots are drawn for all protein, RNA, DNA and oligosaccharide chains in the entry. The first graphic for a chain summarises the proportions of the various outlier classes displayed in the second graphic. The second graphic shows the sequence view annotated by issues in geometry and electron density. Residues are color-coded according to the number of geometric quality criteria for which they contain at least one outlier: green = 0, yellow = 1, orange = 2 and red = 3 or more. A red dot above a residue indicates a poor fit to the electron density (RSRZ > 2). Stretches of 2 or more consecutive residues without any outlier are shown as a green connector. Residues present in the sample, but not in the model, are shown in grey.

- Molecule 1: Proto-oncogene tyrosine-protein kinase receptor Ret

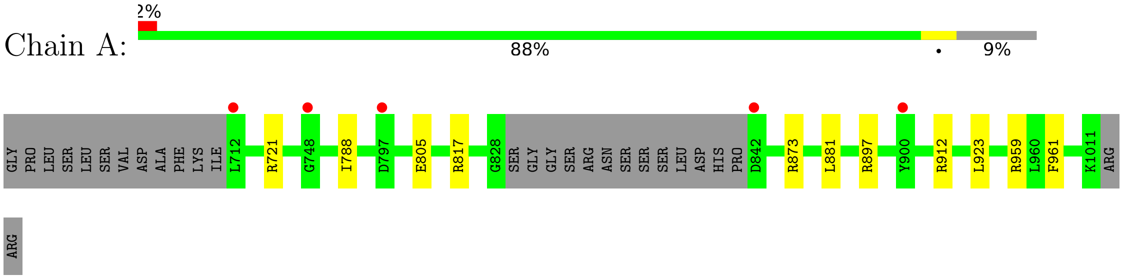

- Molecule 1: Proto-oncogene tyrosine-protein kinase receptor Ret

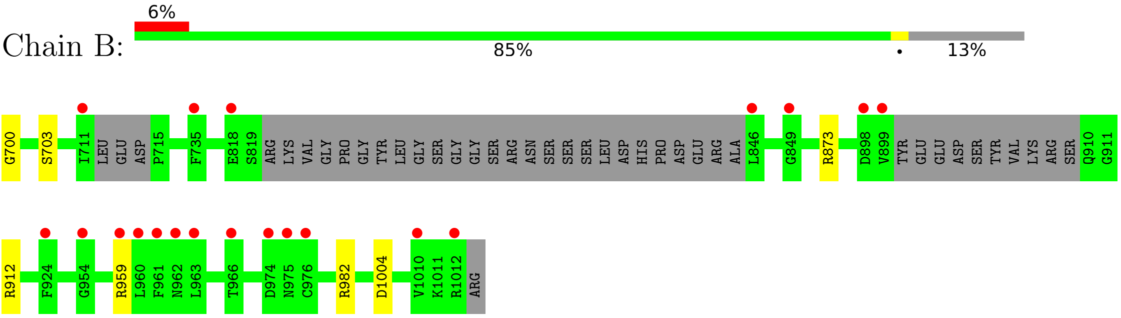

4 Data and refinement statistics (i)

Property	Value	Source
Space group	P 1 21 1	Depositor
Cell constants<br>a, b, c, α, β, γ	50.71Å 80.22Å 79.68Å<br>90.00° 99.40° 90.00°	Depositor
Resolution (Å)	78.63 – 1.64<br>45.73 – 1.64	Depositor<br>EDS
% Data completeness<br>(in resolution range)	97.5 (78.63-1.64)<br>97.5 (45.73-1.64)	Depositor<br>EDS
R_{merge}	(Not available)	Depositor
R_{sym}	0.05	Depositor
$< I/\sigma(I) >$ [1]	2.30 (at 1.64Å)	Xtriage
Refinement program	REFMAC 5.8.0155	Depositor
R, R_{free}	0.191 , 0.218<br>0.198 , 0.221	Depositor<br>DCC
R_{free} test set	3698 reflections (4.92%)	wwPDB-VP
Wilson B-factor (Å^2)	20.6	Xtriage
Anisotropy	0.283	Xtriage
Bulk solvent k_{sol}(e/Å^3), B_{sol}(Å^2)	0.37 , 42.8	EDS
L-test for twinning[2]	$< \|L\| > = 0.49$, $< L^2 > = 0.32$	Xtriage
Estimated twinning fraction	No twinning to report.	Xtriage
F_o,F_c correlation	0.96	EDS
Total number of atoms	4988	wwPDB-VP
Average B, all atoms (Å^2)	26.0	wwPDB-VP

Xtriage's analysis on translational NCS is as follows: *The largest off-origin peak in the Patterson function is 4.97% of the height of the origin peak. No significant pseudotranslation is detected.*

[1]Intensities estimated from amplitudes.

[2]Theoretical values of $< |L| >$, $< L^2 >$ for acentric reflections are 0.5, 0.333 respectively for untwinned datasets, and 0.375, 0.2 for perfectly twinned datasets.

5 Model quality (i)

5.1 Standard geometry (i)

Bond lengths and bond angles in the following residue types are not validated in this section: HJ0

The Z score for a bond length (or angle) is the number of standard deviations the observed value is removed from the expected value. A bond length (or angle) with $|Z| > 5$ is considered an outlier worth inspection. RMSZ is the root-mean-square of all Z scores of the bond lengths (or angles).

Mol	Chain	Bond lengths		Bond angles	
		RMSZ	#|Z| >5	RMSZ	#|Z| >5
1	A	0.54	0/2402	0.80	5/3235 (0.2%)
1	B	0.53	0/2271	0.75	4/3056 (0.1%)
All	All	0.53	0/4673	0.78	9/6291 (0.1%)

There are no bond length outliers.

All (9) bond angle outliers are listed below:

Mol	Chain	Res	Type	Atoms	Z	Observed(°)	Ideal(°)
1	B	873	ARG	NE-CZ-NH1	6.19	123.39	120.30
1	B	912	ARG	NE-CZ-NH1	5.77	123.18	120.30
1	A	897	ARG	NE-CZ-NH1	5.65	123.12	120.30
1	A	912	ARG	NE-CZ-NH1	5.56	123.08	120.30
1	A	817	ARG	NE-CZ-NH1	5.43	123.02	120.30
1	B	959	ARG	NE-CZ-NH1	5.21	122.91	120.30
1	A	873	ARG	NE-CZ-NH1	5.18	122.89	120.30
1	B	982	ARG	NE-CZ-NH1	5.09	122.84	120.30
1	A	959	ARG	NE-CZ-NH1	5.02	122.81	120.30

There are no chirality outliers.

There are no planarity outliers.

5.2 Too-close contacts (i)

In the following table, the Non-H and H(model) columns list the number of non-hydrogen atoms and hydrogen atoms in the chain respectively. The H(added) column lists the number of hydrogen atoms added and optimized by MolProbity. The Clashes column lists the number of clashes within the asymmetric unit, whereas Symm-Clashes lists symmetry-related clashes.

Mol	Chain	Non-H	H(model)	H(added)	Clashes	Symm-Clashes
1	A	2350	0	2374	3	0
1	B	2223	0	2262	1	0
2	A	23	0	0	0	0
2	B	23	0	0	0	0
3	A	212	0	0	0	0
3	B	157	0	0	0	0
All	All	4988	0	4636	4	0

The all-atom clashscore is defined as the number of clashes found per 1000 atoms (including hydrogen atoms). The all-atom clashscore for this structure is 0.

All (4) close contacts within the same asymmetric unit are listed below, sorted by their clash magnitude.

Atom-1	Atom-2	Interatomic distance (Å)	Clash overlap (Å)
1:B:700:GLY:N	1:B:703:SER:HG	2.14	0.45
1:A:788[A]:ILE:HD13	1:A:881:LEU:HD12	1.99	0.43
1:A:923:LEU:HG	1:A:961:PHE:CE1	2.56	0.41
1:A:721[B]:ARG:HA	1:A:721[B]:ARG:HD2	1.84	0.40

There are no symmetry-related clashes.

5.3 Torsion angles (i)

5.3.1 Protein backbone (i)

In the following table, the Percentiles column shows the percent Ramachandran outliers of the chain as a percentile score with respect to all X-ray entries followed by that with respect to entries of similar resolution.

The Analysed column shows the number of residues for which the backbone conformation was analysed, and the total number of residues.

Mol	Chain	Analysed	Favoured	Allowed	Outliers	Percentiles	
1	A	288/314 (92%)	285 (99%)	3 (1%)	0	100	100
1	B	270/314 (86%)	268 (99%)	2 (1%)	0	100	100
All	All	558/628 (89%)	553 (99%)	5 (1%)	0	100	100

There are no Ramachandran outliers to report.

5.3.2 Protein sidechains (i)

In the following table, the Percentiles column shows the percent sidechain outliers of the chain as a percentile score with respect to all X-ray entries followed by that with respect to entries of similar resolution.

The Analysed column shows the number of residues for which the sidechain conformation was analysed, and the total number of residues.

Mol	Chain	Analysed	Rotameric	Outliers	Percentiles	
1	A	254/272 (93%)	253 (100%)	1 (0%)	91	84
1	B	242/272 (89%)	241 (100%)	1 (0%)	91	84
All	All	496/544 (91%)	494 (100%)	2 (0%)	91	84

All (2) residues with a non-rotameric sidechain are listed below:

Mol	Chain	Res	Type
1	A	805	GLU
1	B	1004	ASP

Sometimes sidechains can be flipped to improve hydrogen bonding and reduce clashes. There are no such sidechains identified.

5.3.3 RNA (i)

There are no RNA molecules in this entry.

5.4 Non-standard residues in protein, DNA, RNA chains (i)

There are no non-standard protein/DNA/RNA residues in this entry.

5.5 Carbohydrates (i)

There are no monosaccharides in this entry.

5.6 Ligand geometry (i)

2 ligands are modelled in this entry.

In the following table, the Counts columns list the number of bonds (or angles) for which Mogul statistics could be retrieved, the number of bonds (or angles) that are observed in the model and the number of bonds (or angles) that are defined in the Chemical Component Dictionary. The

Link column lists molecule types, if any, to which the group is linked. The Z score for a bond length (or angle) is the number of standard deviations the observed value is removed from the expected value. A bond length (or angle) with $|Z| > 2$ is considered an outlier worth inspection. RMSZ is the root-mean-square of all Z scores of the bond lengths (or angles).

Mol	Type	Chain	Res	Link	Bond lengths			Bond angles		
					Counts	RMSZ	$\#\|Z\| > 2$	Counts	RMSZ	$\#\|Z\| > 2$
2	HJ0	A	1101	-	24,26,26	1.58	4 (16%)	17,40,40	2.04	5 (29%)
2	HJ0	B	1101	-	24,26,26	1.61	5 (20%)	17,40,40	2.12	6 (35%)

In the following table, the Chirals column lists the number of chiral outliers, the number of chiral centers analysed, the number of these observed in the model and the number defined in the Chemical Component Dictionary. Similar counts are reported in the Torsion and Rings columns. '-' means no outliers of that kind were identified.

Mol	Type	Chain	Res	Link	Chirals	Torsions	Rings
2	HJ0	A	1101	-	-	0/4/18/18	0/4/4/4
2	HJ0	B	1101	-	-	0/4/18/18	0/4/4/4

All (9) bond length outliers are listed below:

Mol	Chain	Res	Type	Atoms	Z	Observed(Å)	Ideal(Å)
2	A	1101	HJ0	C10-C19	4.11	1.47	1.42
2	B	1101	HJ0	C10-C19	4.10	1.47	1.42
2	A	1101	HJ0	C20-N12	-3.99	1.46	1.51
2	B	1101	HJ0	C20-N12	-3.94	1.46	1.51
2	B	1101	HJ0	C11-N12	-2.72	1.34	1.38
2	A	1101	HJ0	C6-N8	-2.43	1.30	1.34
2	A	1101	HJ0	C10-C2	-2.38	1.46	1.50
2	B	1101	HJ0	C6-N8	-2.33	1.30	1.34
2	B	1101	HJ0	C10-C2	-2.07	1.47	1.50

All (11) bond angle outliers are listed below:

Mol	Chain	Res	Type	Atoms	Z	Observed(^{o})	Ideal(^{o})
2	A	1101	HJ0	C10-C2-N3	4.17	121.02	114.79
2	B	1101	HJ0	N14-C15-N16	-3.92	122.55	128.68
2	B	1101	HJ0	C10-C2-N3	3.89	120.61	114.79
2	A	1101	HJ0	O1-C2-C10	-3.49	116.90	121.72
2	B	1101	HJ0	O1-C2-C10	-3.33	117.12	121.72
2	A	1101	HJ0	N14-C15-N16	-3.31	123.51	128.68
2	B	1101	HJ0	C10-C19-C13	-3.21	104.98	107.54
2	A	1101	HJ0	C10-C19-C13	-2.83	105.28	107.54
2	B	1101	HJ0	C7-C6-N8	2.74	125.54	120.07

Continued on next page...

Continued from previous page...

Mol	Chain	Res	Type	Atoms	Z	Observed(^{o})	Ideal(^{o})
2	A	1101	HJ0	C7-C6-N8	2.66	125.37	120.07
2	B	1101	HJ0	C11-N12-C20	2.23	125.88	124.18

There are no chirality outliers.

There are no torsion outliers.

There are no ring outliers.

No monomer is involved in short contacts.

The following is a two-dimensional graphical depiction of Mogul quality analysis of bond lengths, bond angles, torsion angles, and ring geometry for all instances of the Ligand of Interest. In addition, ligands with molecular weight > 250 and outliers as shown on the validation Tables will also be included. For torsion angles, if less then 5% of the Mogul distribution of torsion angles is within 10 degrees of the torsion angle in question, then that torsion angle is considered an outlier. Any bond that is central to one or more torsion angles identified as an outlier by Mogul will be highlighted in the graph. For rings, the root-mean-square deviation (RMSD) between the ring in question and similar rings identified by Mogul is calculated over all ring torsion angles. If the average RMSD is greater than 60 degrees and the minimal RMSD between the ring in question and any Mogul-identified rings is also greater than 60 degrees, then that ring is considered an outlier. The outliers are highlighted in purple. The color gray indicates Mogul did not find sufficient equivalents in the CSD to analyse the geometry.

Ligand HJ0 A 1101

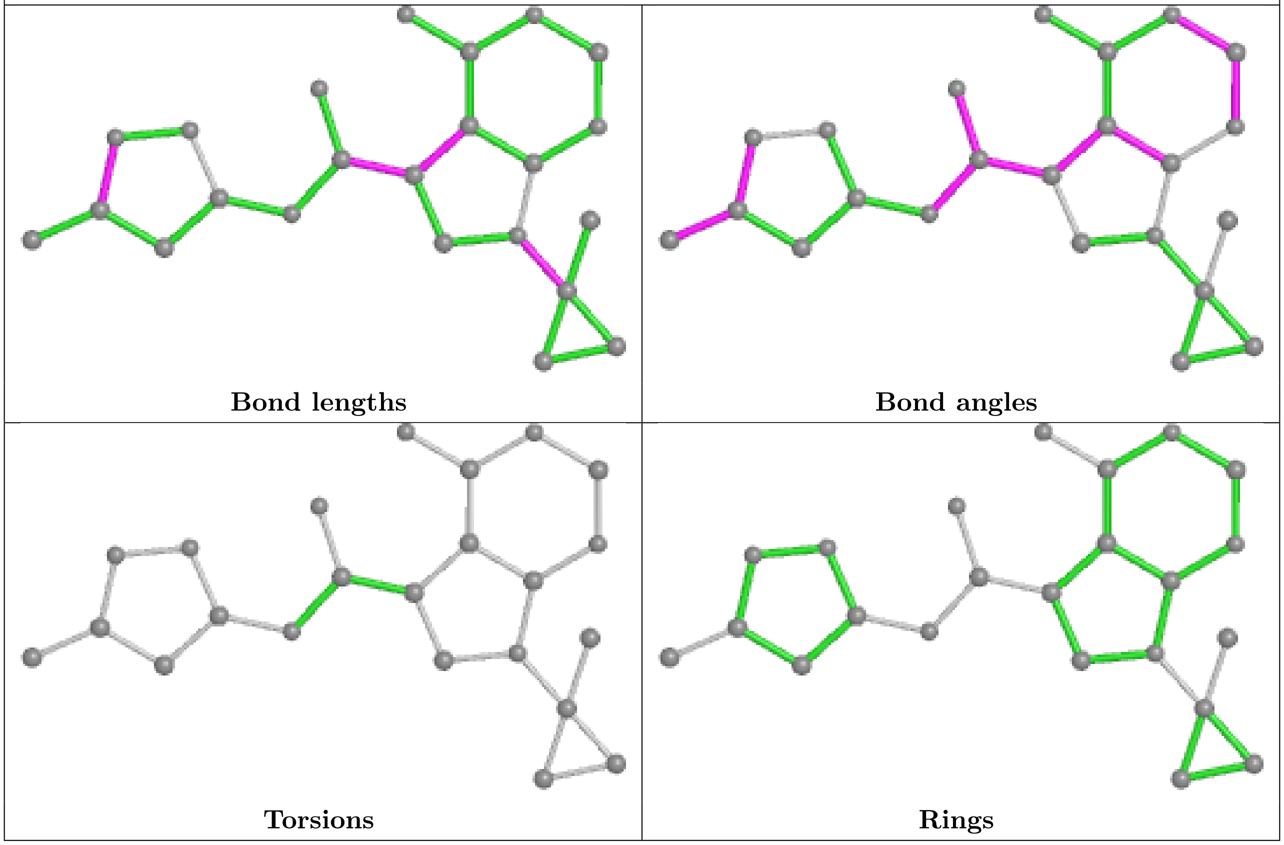

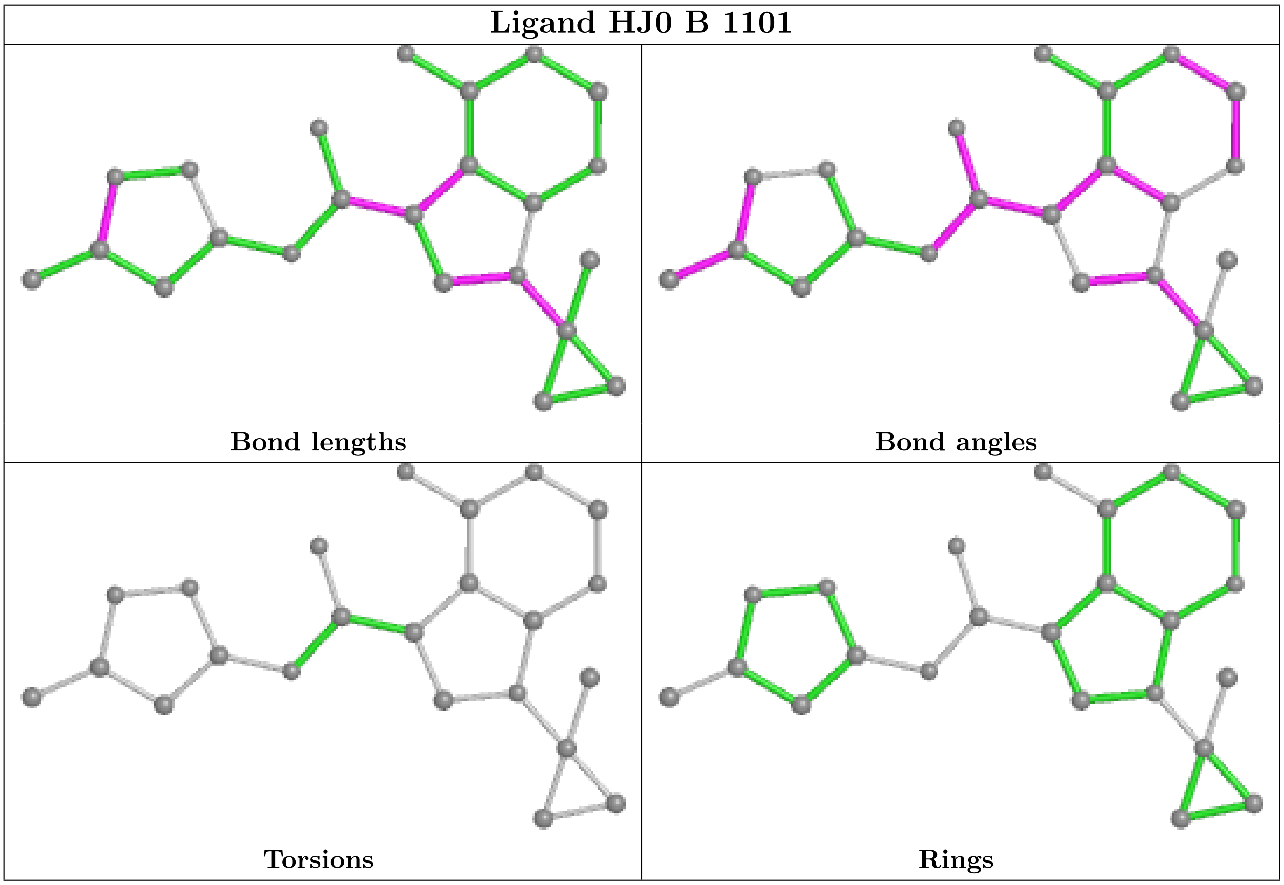

5.7 Other polymers (i)

There are no such residues in this entry.

5.8 Polymer linkage issues (i)

There are no chain breaks in this entry.

6 Fit of model and data (i)

6.1 Protein, DNA and RNA chains (i)

In the following table, the column labelled '#RSRZ> 2' contains the number (and percentage) of RSRZ outliers, followed by percent RSRZ outliers for the chain as percentile scores relative to all X-ray entries and entries of similar resolution. The OWAB column contains the minimum, median, 95th percentile and maximum values of the occupancy-weighted average B-factor per residue. The column labelled 'Q< 0.9' lists the number of (and percentage) of residues with an average occupancy less than 0.9.

Mol	Chain	Analysed	<RSRZ>	#RSRZ>2			OWAB(Å^2)	Q<0.9
1	A	287/314 (91%)	-0.02	5 (1%)	70	71	13, 20, 42, 62	15 (5%)
1	B	274/314 (87%)	0.26	20 (7%)	15	13	15, 26, 47, 67	9 (3%)
All	All	561/628 (89%)	0.12	25 (4%)	33	30	13, 23, 45, 67	24 (4%)

All (25) RSRZ outliers are listed below:

Mol	Chain	Res	Type	RSRZ
1	B	975	ASN	3.5
1	A	797	ASP	2.9
1	A	900	TYR	2.9
1	B	818	GLU	2.8
1	B	846	LEU	2.8
1	B	961	PHE	2.7
1	A	842	ASP	2.6
1	A	712	LEU	2.6
1	B	899	VAL	2.5
1	B	735	PHE	2.4
1	B	960	LEU	2.4
1	B	959	ARG	2.4
1	B	954	GLY	2.4
1	A	748	GLY	2.3
1	B	974	ASP	2.3
1	B	849	GLY	2.3
1	B	711	ILE	2.3
1	B	898	ASP	2.2
1	B	976	CYS	2.1
1	B	1010	VAL	2.1
1	B	924	PHE	2.1
1	B	963	LEU	2.1
1	B	1012	ARG	2.0
1	B	966	THR	2.0

Continued on next page...

Continued from previous page...

Mol	Chain	Res	Type	RSRZ
1	B	962	ASN	2.0

6.2 Non-standard residues in protein, DNA, RNA chains (i)

There are no non-standard protein/DNA/RNA residues in this entry.

6.3 Carbohydrates (i)

There are no monosaccharides in this entry.

6.4 Ligands (i)

In the following table, the Atoms column lists the number of modelled atoms in the group and the number defined in the chemical component dictionary. The B-factors column lists the minimum, median, 95th percentile and maximum values of B factors of atoms in the group. The column labelled 'Q< 0.9' lists the number of atoms with occupancy less than 0.9.

Mol	Type	Chain	Res	Atoms	RSCC	RSR	B-factors($Å^2$)	Q<0.9
2	HJ0	B	1101	23/23	0.95	0.08	16,18,21,22	0
2	HJ0	A	1101	23/23	0.96	0.08	14,16,18,21	0

The following is a graphical depiction of the model fit to experimental electron density of all instances of the Ligand of Interest. In addition, ligands with molecular weight > 250 and outliers as shown on the geometry validation Tables will also be included. Each fit is shown from different orientation to approximate a three-dimensional view.

Electron density around HJ0 B 1101:

2mF$_o$-DF$_c$ (at 0.7 rmsd) in gray
mF$_o$-DF$_c$ (at 3 rmsd) in purple (negative)
and green (positive)

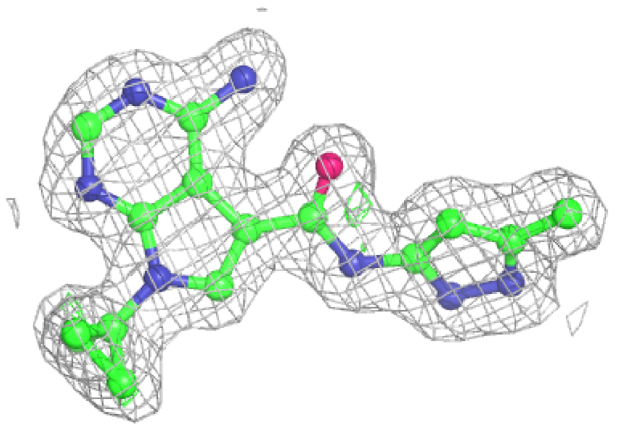

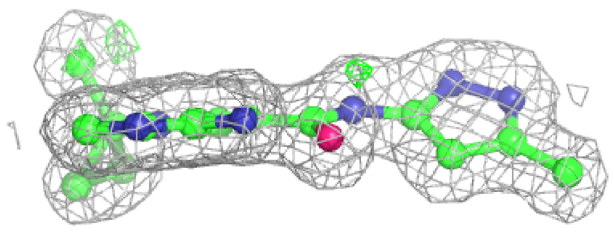

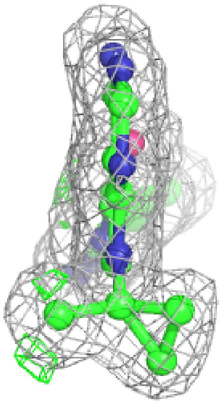

Electron density around HJ0 A 1101:

2mF$_o$-DF$_c$ (at 0.7 rmsd) in gray
mF$_o$-DF$_c$ (at 3 rmsd) in purple (negative)
and green (positive)

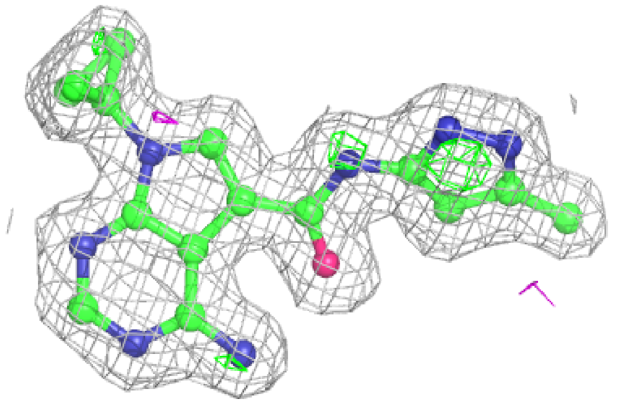

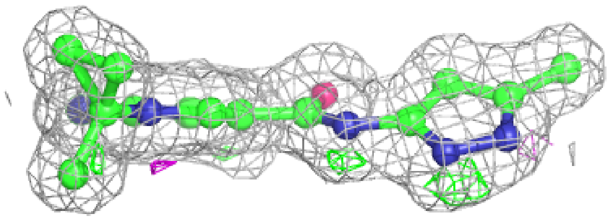

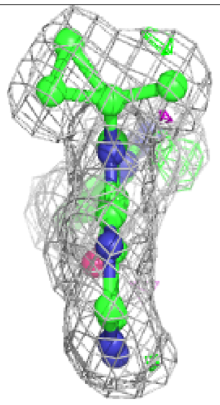

6.5 Other polymers

There are no such residues in this entry.
