# Supplementary material for: Vepafestinib is a pharmacologically advanced RET-selective inhibitor with high CNS penetration and inhibitory activity against RET solvent front mutations
Source: Nat Cancer. 2023 Sep 21;4(9):1345–61. doi: 10.1038/s43018-023-00630-y (PMC10518257; doi:10.1038/s43018-023-00630-y)
Supplement: Supplementary file 5 — Full X-ray structure validation report for RET in complex with selpercatinib. [file 43018_2023_630_MOESM5_ESM.pdf]

Full wwPDB X-ray Structure Validation Report (i)

Jan 24, 2022 – 06:09 PM JST

PDB ID	:	7DU8
Title	:	Crystal structure of human Proto-oncogene tyrosine-protein kinase receptor Ret in complex with Selpercatinib
Authors	:	Miyazaki, I.; Ishida, K.; Suzuki, T.
Deposited on	:	2021-01-08
Resolution	:	2.75 Å(reported)

This is a Full wwPDB X-ray Structure Validation Report for a publicly released PDB entry.

We welcome your comments at *validation@mail.wwpdb.org*
A user guide is available at
https://www.wwpdb.org/validation/2017/XrayValidationReportHelp
with specific help available everywhere you see the (i) symbol.

The following versions of software and data (see references (i)) were used in the production of this report:

MolProbity	:	4.02b-467
Mogul	:	1.8.5 (274361), CSD as541be (2020)
Xtriage (Phenix)	:	1.13
EDS	:	2.26
buster-report	:	1.1.7 (2018)
Percentile statistics	:	20191225.v01 (using entries in the PDB archive December 25th 2019)
Refmac	:	5.8.0158
CCP4	:	7.0.044 (Gargrove)
Ideal geometry (proteins)	:	Engh & Huber (2001)
Ideal geometry (DNA, RNA)	:	Parkinson et al. (1996)
Validation Pipeline (wwPDB-VP)	:	2.26

1 Overall quality at a glance (i)

The following experimental techniques were used to determine the structure:
X-RAY DIFFRACTION

The reported resolution of this entry is 2.75 Å.

Percentile scores (ranging between 0-100) for global validation metrics of the entry are shown in the following graphic. The table shows the number of entries on which the scores are based.

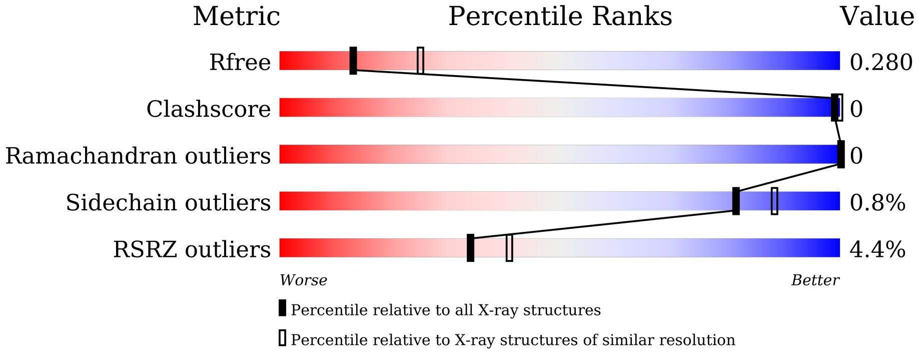

Metric	Whole archive (#Entries)	Similar resolution (#Entries, resolution range(Å))
R_{free}	130704	1235 (2.78-2.74)
Clashscore	141614	1277 (2.78-2.74)
Ramachandran outliers	138981	1257 (2.78-2.74)
Sidechain outliers	138945	1257 (2.78-2.74)
RSRZ outliers	127900	1207 (2.78-2.74)

The table below summarises the geometric issues observed across the polymeric chains and their fit to the electron density. The red, orange, yellow and green segments of the lower bar indicate the fraction of residues that contain outliers for >=3, 2, 1 and 0 types of geometric quality criteria respectively. A grey segment represents the fraction of residues that are not modelled. The numeric value for each fraction is indicated below the corresponding segment, with a dot representing fractions <=5% The upper red bar (where present) indicates the fraction of residues that have poor fit to the electron density. The numeric value is given above the bar.

Mol	Chain	Length	Quality of chain
1	A	314	2% / 89% • 9%
1	B	314	6% / 87% • 11%

2 Entry composition (i)

There are 3 unique types of molecules in this entry. The entry contains 4715 atoms, of which 0 are hydrogens and 0 are deuteriums.

In the tables below, the ZeroOcc column contains the number of atoms modelled with zero occupancy, the AltConf column contains the number of residues with at least one atom in alternate conformation and the Trace column contains the number of residues modelled with at most 2 atoms.

- Molecule 1 is a protein called Proto-oncogene tyrosine-protein kinase receptor Ret.

Mol	Chain	Residues	Atoms					ZeroOcc	AltConf	Trace
1	A	286	Total 2331	C 1492	N 408	O 417	S 14	56	3	0
1	B	279	Total 2248	C 1446	N 395	O 393	S 14	67	1	0

There are 10 discrepancies between the modelled and reference sequences:

Chain	Residue	Modelled	Actual	Comment	Reference
A	700	GLY	-	expression tag	UNP P07949
A	701	PRO	-	expression tag	UNP P07949
A	702	LEU	-	expression tag	UNP P07949
A	703	SER	-	expression tag	UNP P07949
A	704	LEU	-	expression tag	UNP P07949
B	700	GLY	-	expression tag	UNP P07949
B	701	PRO	-	expression tag	UNP P07949
B	702	LEU	-	expression tag	UNP P07949
B	703	SER	-	expression tag	UNP P07949
B	704	LEU	-	expression tag	UNP P07949

- Molecule 2 is Selpercatinib (three-letter code: Q6G) (formula: $C_{29}H_{31}N_7O_3$) (labeled as "Ligand of Interest" by depositor).

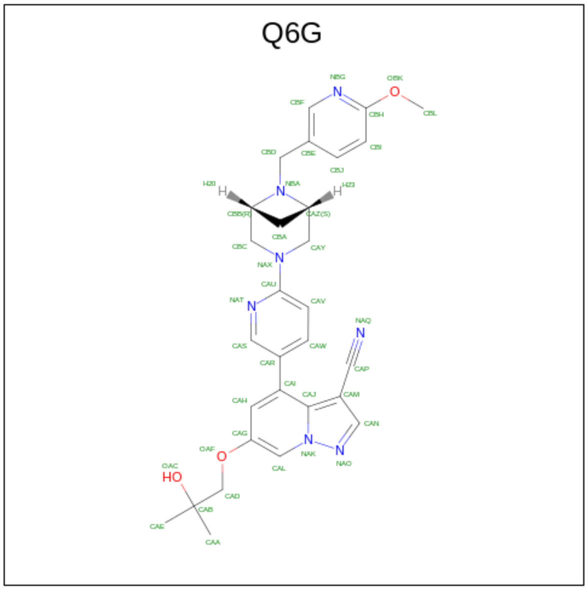

Mol	Chain	Residues	Atoms	ZeroOcc	AltConf
2	A	1	Total C N O<br>39 29 7 3	0	0
2	B	1	Total C N O<br>39 29 7 3	0	0

- Molecule 3 is water.

Mol	Chain	Residues	Atoms	ZeroOcc	AltConf
3	A	36	Total O<br>36 36	0	0
3	B	22	Total O<br>22 22	0	0

3 Residue-property plots (i)

These plots are drawn for all protein, RNA, DNA and oligosaccharide chains in the entry. The first graphic for a chain summarises the proportions of the various outlier classes displayed in the second graphic. The second graphic shows the sequence view annotated by issues in geometry and electron density. Residues are color-coded according to the number of geometric quality criteria for which they contain at least one outlier: green = 0, yellow = 1, orange = 2 and red = 3 or more. A red dot above a residue indicates a poor fit to the electron density (RSRZ > 2). Stretches of 2 or more consecutive residues without any outlier are shown as a green connector. Residues present in the sample, but not in the model, are shown in grey.

- Molecule 1: Proto-oncogene tyrosine-protein kinase receptor Ret

Chain A:
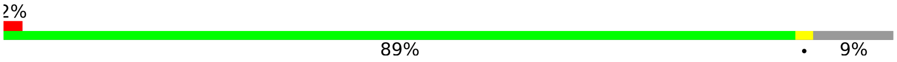

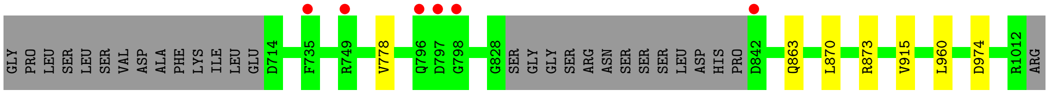

- Molecule 1: Proto-oncogene tyrosine-protein kinase receptor Ret

Chain B:
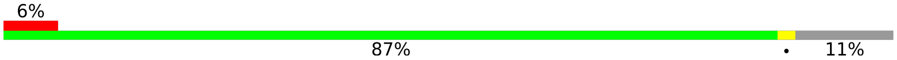

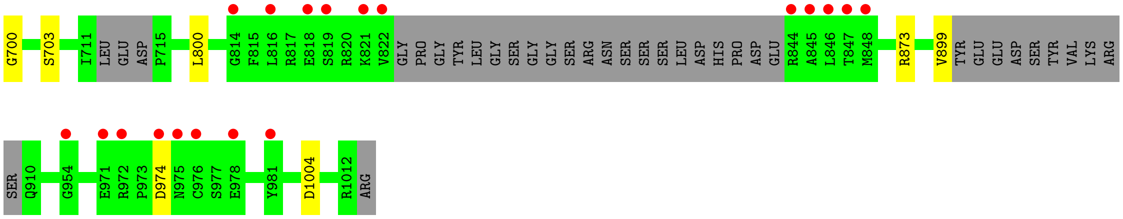

4 Data and refinement statistics (i)

Property	Value	Source
Space group	P 1 21 1	Depositor
Cell constants<br>a, b, c, α, β, γ	50.77Å 80.27Å 79.91Å<br>90.00° 99.61° 90.00°	Depositor
Resolution (Å)	78.79 – 2.75<br>42.47 – 2.75	Depositor<br>EDS
% Data completeness<br>(in resolution range)	94.1 (78.79-2.75)<br>94.2 (42.47-2.75)	Depositor<br>EDS
R_{merge}	(Not available)	Depositor
R_{sym}	0.11	Depositor
$< I/\sigma(I) >$ [1]	2.35 (at 2.77Å)	Xtriage
Refinement program	REFMAC 5.8.0155	Depositor
R, R_{free}	0.214 , 0.277<br>0.214 , 0.280	Depositor<br>DCC
R_{free} test set	680 reflections (4.36%)	wwPDB-VP
Wilson B-factor (Å^2)	40.6	Xtriage
Anisotropy	0.329	Xtriage
Bulk solvent k_{sol}(e/Å^3), B_{sol}(Å^2)	0.36 , 46.9	EDS
L-test for twinning[2]	$< \|L\| > = 0.50$, $< L^2 > = 0.33$	Xtriage
Estimated twinning fraction	No twinning to report.	Xtriage
F_o,F_c correlation	0.92	EDS
Total number of atoms	4715	wwPDB-VP
Average B, all atoms (Å^2)	46.0	wwPDB-VP

Xtriage's analysis on translational NCS is as follows: *The largest off-origin peak in the Patterson function is 4.89% of the height of the origin peak. No significant pseudotranslation is detected.*

[1] Intensities estimated from amplitudes.
[2] Theoretical values of $< |L| >$, $< L^2 >$ for acentric reflections are 0.5, 0.333 respectively for untwinned datasets, and 0.375, 0.2 for perfectly twinned datasets.

5 Model quality (i)

5.1 Standard geometry (i)

Bond lengths and bond angles in the following residue types are not validated in this section: Q6G

The Z score for a bond length (or angle) is the number of standard deviations the observed value is removed from the expected value. A bond length (or angle) with $|Z| > 5$ is considered an outlier worth inspection. RMSZ is the root-mean-square of all Z scores of the bond lengths (or angles).

Mol	Chain	Bond lengths		Bond angles	
		RMSZ	$\#\|Z\| >5$	RMSZ	$\#\|Z\| >5$
1	A	0.57	0/2383	0.74	1/3207 (0.0%)
1	B	0.59	1/2296 (0.0%)	0.75	2/3087 (0.1%)
All	All	0.58	1/4679 (0.0%)	0.74	3/6294 (0.0%)

All (1) bond length outliers are listed below:

Mol	Chain	Res	Type	Atoms	Z	Observed(Å)	Ideal(Å)
1	B	899	VAL	CB-CG2	5.29	1.64	1.52

All (3) bond angle outliers are listed below:

Mol	Chain	Res	Type	Atoms	Z	Observed(^{o})	Ideal(^{o})
1	B	873	ARG	NE-CZ-NH1	5.46	123.03	120.30
1	B	800	LEU	CB-CG-CD2	5.41	120.20	111.00
1	A	873	ARG	NE-CZ-NH1	5.20	122.90	120.30

There are no chirality outliers.

There are no planarity outliers.

5.2 Too-close contacts (i)

In the following table, the Non-H and H(model) columns list the number of non-hydrogen atoms and hydrogen atoms in the chain respectively. The H(added) column lists the number of hydrogen atoms added and optimized by MolProbity. The Clashes column lists the number of clashes within the asymmetric unit, whereas Symm-Clashes lists symmetry-related clashes.

Mol	Chain	Non-H	H(model)	H(added)	Clashes	Symm-Clashes
1	A	2331	0	2356	2	0
1	B	2248	0	2304	1	0

Continued on next page...

Continued from previous page...

Mol	Chain	Non-H	H(model)	H(added)	Clashes	Symm-Clashes
2	A	39	0	0	0	0
2	B	39	0	0	0	0
3	A	36	0	0	0	0
3	B	22	0	0	0	0
All	All	4715	0	4660	3	0

The all-atom clashscore is defined as the number of clashes found per 1000 atoms (including hydrogen atoms). The all-atom clashscore for this structure is 0.

All (3) close contacts within the same asymmetric unit are listed below, sorted by their clash magnitude.

Atom-1	Atom-2	Interatomic distance (Å)	Clash overlap (Å)
1:B:700:GLY:N	1:B:703:SER:HG	2.03	0.57
1:A:778:VAL:HG13	1:A:870:LEU:HD21	1.92	0.52
1:A:915:VAL:HG12	1:A:960:LEU:HD23	1.98	0.46

There are no symmetry-related clashes.

5.3 Torsion angles (i)

5.3.1 Protein backbone (i)

In the following table, the Percentiles column shows the percent Ramachandran outliers of the chain as a percentile score with respect to all X-ray entries followed by that with respect to entries of similar resolution.

The Analysed column shows the number of residues for which the backbone conformation was analysed, and the total number of residues.

Mol	Chain	Analysed	Favoured	Allowed	Outliers	Percentiles	
1	A	285/314 (91%)	274 (96%)	11 (4%)	0	100	100
1	B	272/314 (87%)	265 (97%)	7 (3%)	0	100	100
All	All	557/628 (89%)	539 (97%)	18 (3%)	0	100	100

There are no Ramachandran outliers to report.

5.3.2 Protein sidechains (i)

In the following table, the Percentiles column shows the percent sidechain outliers of the chain as a percentile score with respect to all X-ray entries followed by that with respect to entries of similar resolution.

The Analysed column shows the number of residues for which the sidechain conformation was analysed, and the total number of residues.

Mol	Chain	Analysed	Rotameric	Outliers	Percentiles	
1	A	251/272 (92%)	249 (99%)	2 (1%)	81	88
1	B	243/272 (89%)	241 (99%)	2 (1%)	81	88
All	All	494/544 (91%)	490 (99%)	4 (1%)	81	88

All (4) residues with a non-rotameric sidechain are listed below:

Mol	Chain	Res	Type
1	A	863	GLN
1	A	974	ASP
1	B	974	ASP
1	B	1004	ASP

Sometimes sidechains can be flipped to improve hydrogen bonding and reduce clashes. There are no such sidechains identified.

5.3.3 RNA (i)

There are no RNA molecules in this entry.

5.4 Non-standard residues in protein, DNA, RNA chains (i)

There are no non-standard protein/DNA/RNA residues in this entry.

5.5 Carbohydrates (i)

There are no monosaccharides in this entry.

5.6 Ligand geometry (i)

2 ligands are modelled in this entry.

In the following table, the Counts columns list the number of bonds (or angles) for which Mogul statistics could be retrieved, the number of bonds (or angles) that are observed in the model and the number of bonds (or angles) that are defined in the Chemical Component Dictionary. The Link column lists molecule types, if any, to which the group is linked. The Z score for a bond length (or angle) is the number of standard deviations the observed value is removed from the expected value. A bond length (or angle) with $|Z| > 2$ is considered an outlier worth inspection. RMSZ is the root-mean-square of all Z scores of the bond lengths (or angles).

Mol	Type	Chain	Res	Link	Bond lengths			Bond angles		
					Counts	RMSZ	$\#\|Z\| > 2$	Counts	RMSZ	$\#\|Z\| > 2$
2	Q6G	B	1101	-	41,44,44	1.00	1 (2%)	42,65,65	2.42	16 (38%)
2	Q6G	A	1101	-	41,44,44	1.00	0	42,65,65	2.35	13 (30%)

In the following table, the Chirals column lists the number of chiral outliers, the number of chiral centers analysed, the number of these observed in the model and the number defined in the Chemical Component Dictionary. Similar counts are reported in the Torsion and Rings columns. '-' means no outliers of that kind were identified.

Mol	Type	Chain	Res	Link	Chirals	Torsions	Rings
2	Q6G	B	1101	-	-	3/20/42/42	0/5/6/6
2	Q6G	A	1101	-	-	2/20/42/42	0/5/6/6

All (1) bond length outliers are listed below:

Mol	Chain	Res	Type	Atoms	Z	Observed(Å)	Ideal(Å)
2	B	1101	Q6G	CBC-CBB	2.09	1.55	1.52

All (29) bond angle outliers are listed below:

Mol	Chain	Res	Type	Atoms	Z	Observed(^{o})	Ideal(^{o})
2	A	1101	Q6G	CAN-NAO-NAK	8.00	109.52	103.70
2	B	1101	Q6G	CAN-NAO-NAK	7.96	109.49	103.70
2	B	1101	Q6G	CBF-NBG-CBH	6.74	122.55	116.63
2	A	1101	Q6G	CBF-NBG-CBH	6.67	122.49	116.63
2	B	1101	Q6G	CBI-CBH-NBG	-3.95	118.98	124.87
2	A	1101	Q6G	CBI-CBH-NBG	-3.87	119.10	124.87
2	B	1101	Q6G	OBK-CBH-CBI	3.78	123.29	116.71
2	A	1101	Q6G	OBK-CBH-CBI	3.73	123.19	116.71
2	B	1101	Q6G	CBL-OBK-CBH	3.40	122.70	117.36
2	B	1101	Q6G	CAS-NAT-CAU	3.15	123.05	117.30
2	A	1101	Q6G	CAS-NAT-CAU	3.12	123.00	117.30
2	A	1101	Q6G	CAI-CAJ-NAK	-3.04	117.65	122.64
2	B	1101	Q6G	NAT-CAU-NAX	2.97	121.23	116.79

Continued on next page...

Continued from previous page...

Mol	Chain	Res	Type	Atoms	Z	Observed(^{o})	Ideal(^{o})
2	B	1101	Q6G	CAI-CAJ-NAK	-2.96	117.79	122.64
2	A	1101	Q6G	CAN-CAM-CAP	-2.88	122.70	126.61
2	A	1101	Q6G	CBL-OBK-CBH	2.78	121.72	117.36
2	B	1101	Q6G	CAW-CAR-CAI	-2.73	116.27	120.86
2	A	1101	Q6G	CAW-CAR-CAI	-2.67	116.36	120.86
2	A	1101	Q6G	CAR-CAS-NAT	-2.61	120.02	124.32
2	B	1101	Q6G	CAR-CAS-NAT	-2.52	120.17	124.32
2	A	1101	Q6G	CAV-CAU-NAT	-2.49	118.83	123.41
2	B	1101	Q6G	CAD-OAF-CAG	2.47	125.54	117.81
2	B	1101	Q6G	CAV-CAU-NAT	-2.42	118.97	123.41
2	B	1101	Q6G	CAN-CAM-CAP	-2.41	123.33	126.61
2	B	1101	Q6G	CAS-CAR-CAI	2.23	125.38	121.72
2	A	1101	Q6G	NAT-CAU-NAX	2.21	120.10	116.79
2	A	1101	Q6G	CAD-OAF-CAG	2.15	124.56	117.81
2	B	1101	Q6G	CBD-CBE-CBJ	2.04	124.61	120.77
2	B	1101	Q6G	CAR-CAI-CAJ	2.03	125.09	122.25

There are no chirality outliers.

All (5) torsion outliers are listed below:

Mol	Chain	Res	Type	Atoms
2	B	1101	Q6G	NAT-CAU-NAX-CBC
2	A	1101	Q6G	CBE-CBD-NBA-CBB
2	B	1101	Q6G	CAV-CAU-NAX-CBC
2	B	1101	Q6G	NBA-CBD-CBE-CBF
2	A	1101	Q6G	NAT-CAU-NAX-CBC

There are no ring outliers.

No monomer is involved in short contacts.

The following is a two-dimensional graphical depiction of Mogul quality analysis of bond lengths, bond angles, torsion angles, and ring geometry for all instances of the Ligand of Interest. In addition, ligands with molecular weight > 250 and outliers as shown on the validation Tables will also be included. For torsion angles, if less then 5% of the Mogul distribution of torsion angles is within 10 degrees of the torsion angle in question, then that torsion angle is considered an outlier. Any bond that is central to one or more torsion angles identified as an outlier by Mogul will be highlighted in the graph. For rings, the root-mean-square deviation (RMSD) between the ring in question and similar rings identified by Mogul is calculated over all ring torsion angles. If the average RMSD is greater than 60 degrees and the minimal RMSD between the ring in question and any Mogul-identified rings is also greater than 60 degrees, then that ring is considered an outlier. The outliers are highlighted in purple. The color gray indicates Mogul did not find sufficient equivalents in the CSD to analyse the geometry.

Ligand Q6G B 1101

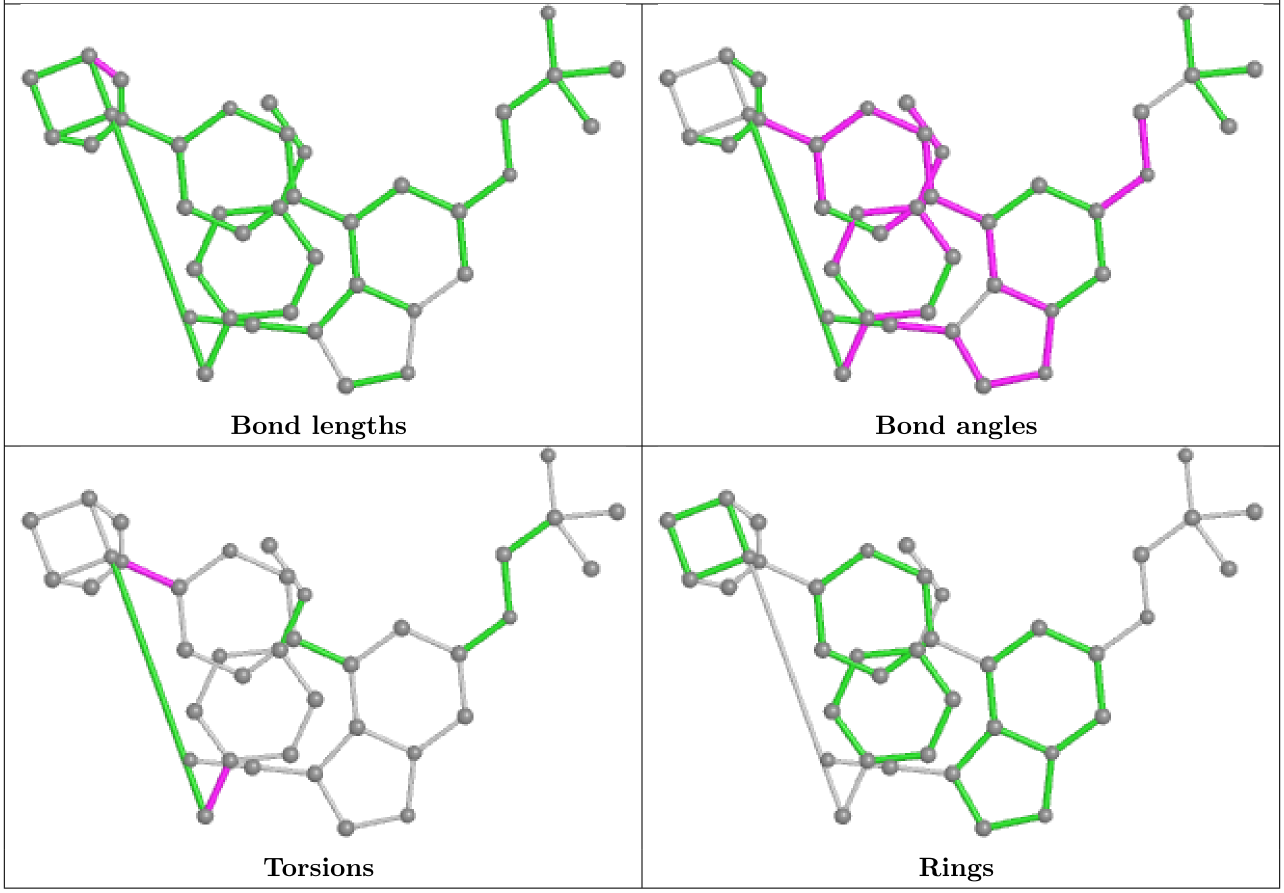

Ligand Q6G A 1101

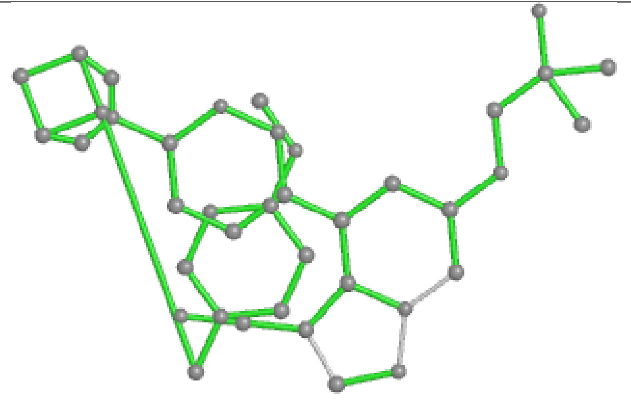

Bond lengths

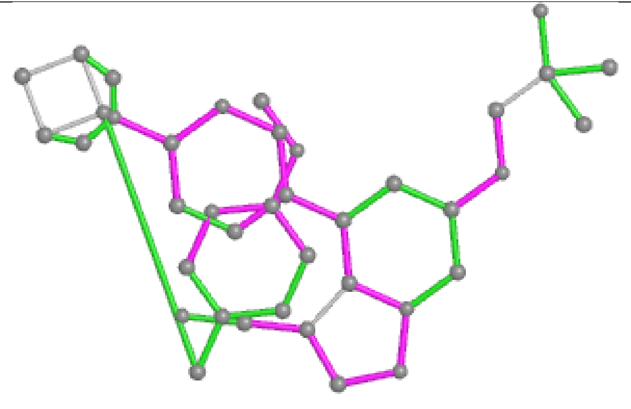

Bond angles

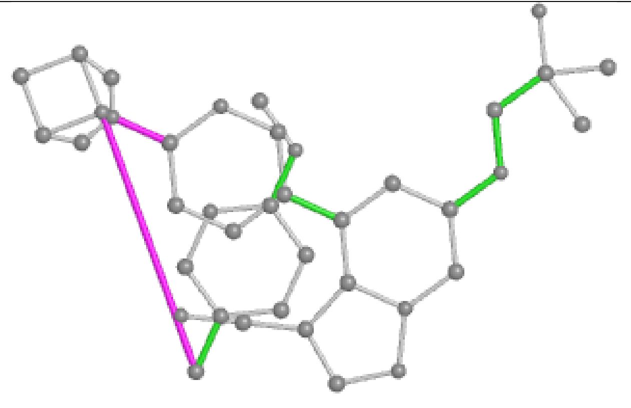

Torsions

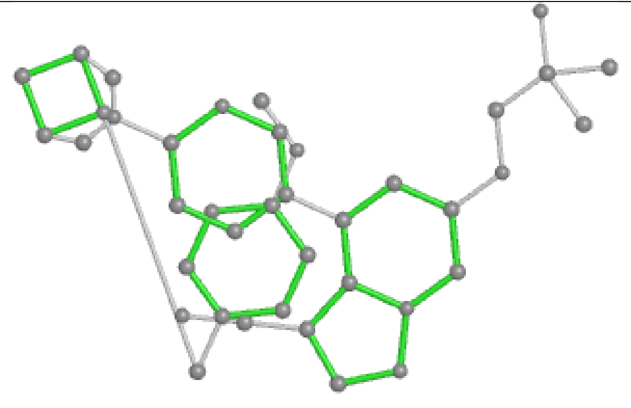

Rings

5.7 Other polymers (i)

There are no such residues in this entry.

5.8 Polymer linkage issues (i)

There are no chain breaks in this entry.

6 Fit of model and data (i)

6.1 Protein, DNA and RNA chains (i)

In the following table, the column labelled '#RSRZ> 2' contains the number (and percentage) of RSRZ outliers, followed by percent RSRZ outliers for the chain as percentile scores relative to all X-ray entries and entries of similar resolution. The OWAB column contains the minimum, median, 95th percentile and maximum values of the occupancy-weighted average B-factor per residue. The column labelled 'Q< 0.9' lists the number of (and percentage) of residues with an average occupancy less than 0.9.

Mol	Chain	Analysed	<RSRZ>	#RSRZ>2			OWAB(Å^2)	Q<0.9
1	A	286/314 (91%)	0.15	6 (2%)	63	72	27, 40, 68, 87	20 (6%)
1	B	279/314 (88%)	0.44	19 (6%)	17	20	31, 44, 77, 106	24 (8%)
All	All	565/628 (89%)	0.29	25 (4%)	34	41	27, 43, 73, 106	44 (7%)

All (25) RSRZ outliers are listed below:

Mol	Chain	Res	Type	RSRZ
1	B	821	LYS	4.8
1	A	797	ASP	4.5
1	B	845	ALA	4.1
1	B	978	GLU	4.0
1	B	819	SER	3.8
1	B	848	MET	3.8
1	B	975	ASN	3.7
1	B	972	ARG	3.7
1	B	822	VAL	3.7
1	B	846	LEU	3.1
1	B	976	CYS	3.0
1	B	844	ARG	2.9
1	B	814	GLY	2.7
1	B	818	GLU	2.7
1	B	971	GLU	2.7
1	B	816	LEU	2.7
1	A	735	PHE	2.7
1	B	981	TYR	2.5
1	B	847	THR	2.4
1	A	749	ARG	2.3
1	A	842	ASP	2.2
1	A	798	GLY	2.1
1	B	954	GLY	2.1
1	A	796	GLN	2.1

Continued on next page...

Continued from previous page...

Mol	Chain	Res	Type	RSRZ
1	B	974	ASP	2.0

6.2 Non-standard residues in protein, DNA, RNA chains (i)

There are no non-standard protein/DNA/RNA residues in this entry.

6.3 Carbohydrates (i)

There are no monosaccharides in this entry.

6.4 Ligands (i)

In the following table, the Atoms column lists the number of modelled atoms in the group and the number defined in the chemical component dictionary. The B-factors column lists the minimum, median, 95th percentile and maximum values of B factors of atoms in the group. The column labelled 'Q< 0.9' lists the number of atoms with occupancy less than 0.9.

Mol	Type	Chain	Res	Atoms	RSCC	RSR	B-factors($Å^2$)	Q<0.9
2	Q6G	A	1101	39/39	0.94	0.18	34,39,50,52	0
2	Q6G	B	1101	39/39	0.94	0.21	29,38,50,53	0

The following is a graphical depiction of the model fit to experimental electron density of all instances of the Ligand of Interest. In addition, ligands with molecular weight > 250 and outliers as shown on the geometry validation Tables will also be included. Each fit is shown from different orientation to approximate a three-dimensional view.

Electron density around Q6G A 1101:

$2mF_o$-DF_c (at 0.7 rmsd) in gray
mF_o-DF_c (at 3 rmsd) in purple (negative)
and green (positive)

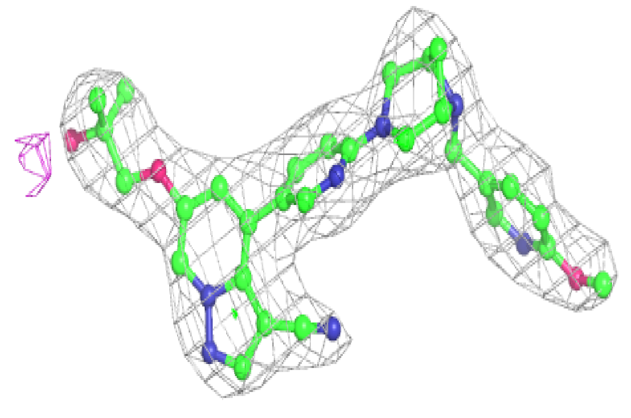

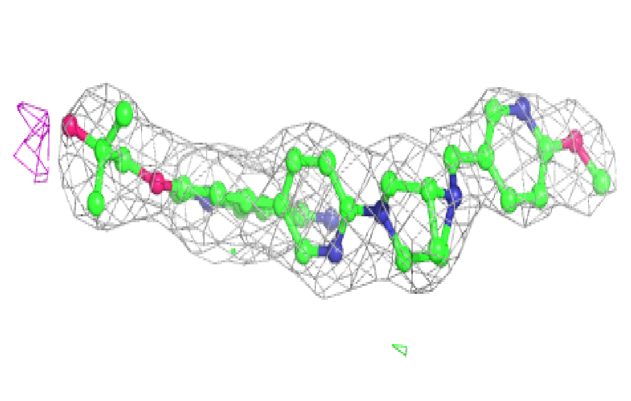

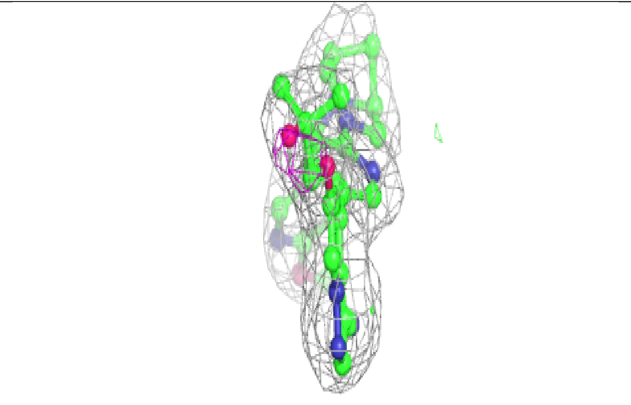

Electron density around Q6G B 1101:

$2mF_o$-DF_c (at 0.7 rmsd) in gray
mF_o-DF_c (at 3 rmsd) in purple (negative)
and green (positive)

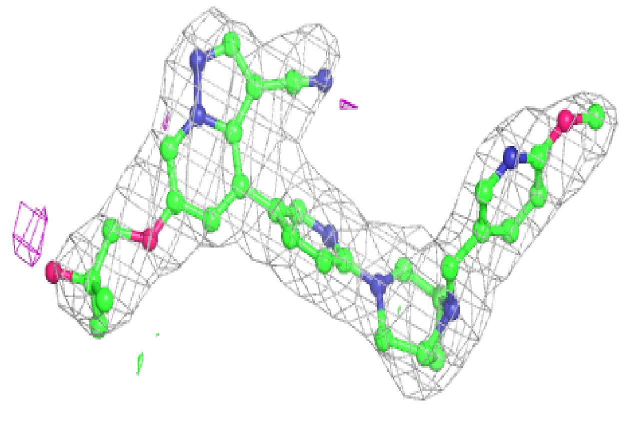

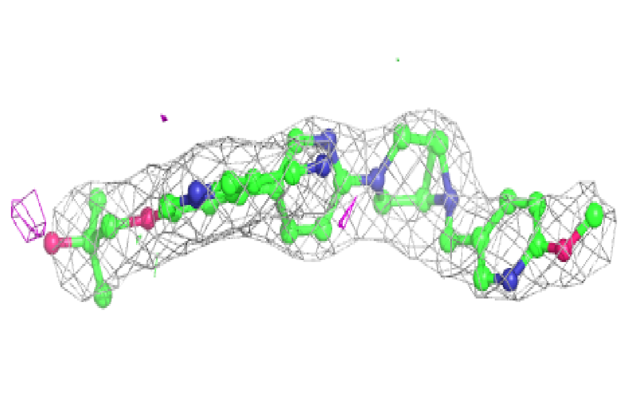

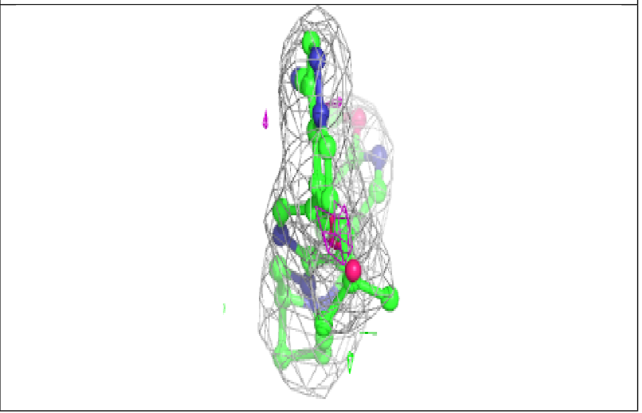

6.5 Other polymers (i)

There are no such residues in this entry.
