# Supplementary material for: Vepafestinib is a pharmacologically advanced RET-selective inhibitor with high CNS penetration and inhibitory activity against RET solvent front mutations
Source: Nat Cancer. 2023 Sep 21;4(9):1345–61. doi: 10.1038/s43018-023-00630-y (PMC10518257; doi:10.1038/s43018-023-00630-y)
Supplement: Supplementary file 7 — This file contains unprocessed images of immunoblots used in Figs. 3, 4 and 7 and Extended Data Fig. 4. [file 43018_2023_630_MOESM7_ESM.pdf]

Source Data Fig. 3a

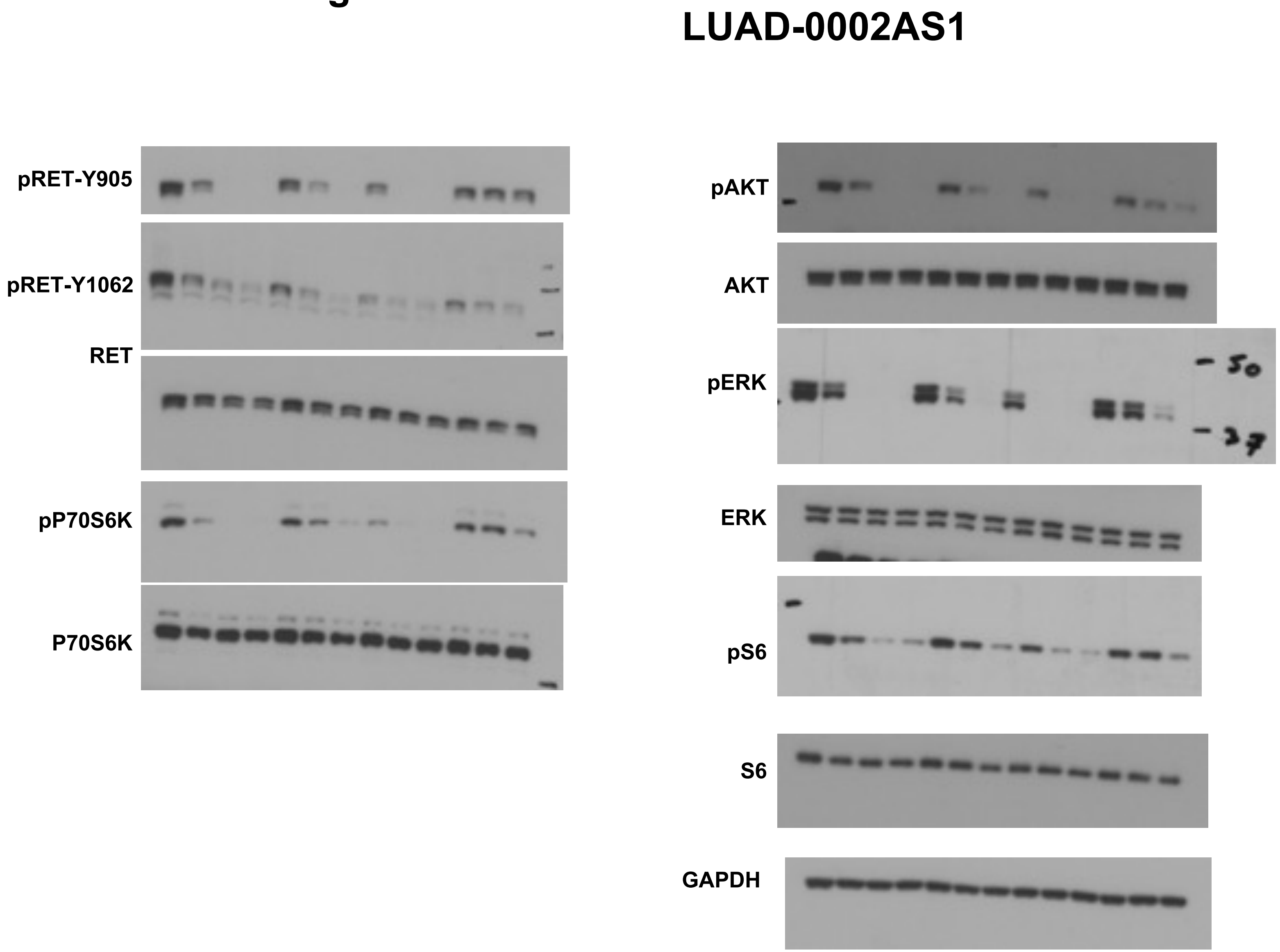

Membranes were cut and each piece probed for the proteins indicated. The blots shown are the entire piece probed and taken directly from the x-ray film without any further modification. Where multiple bands are evident, the arrow indicates the protein of interest. Black check (pen) marks on some blots indicate molecular weight markers placed on film after development.

Source Data Fig. 3a

ECLC5B

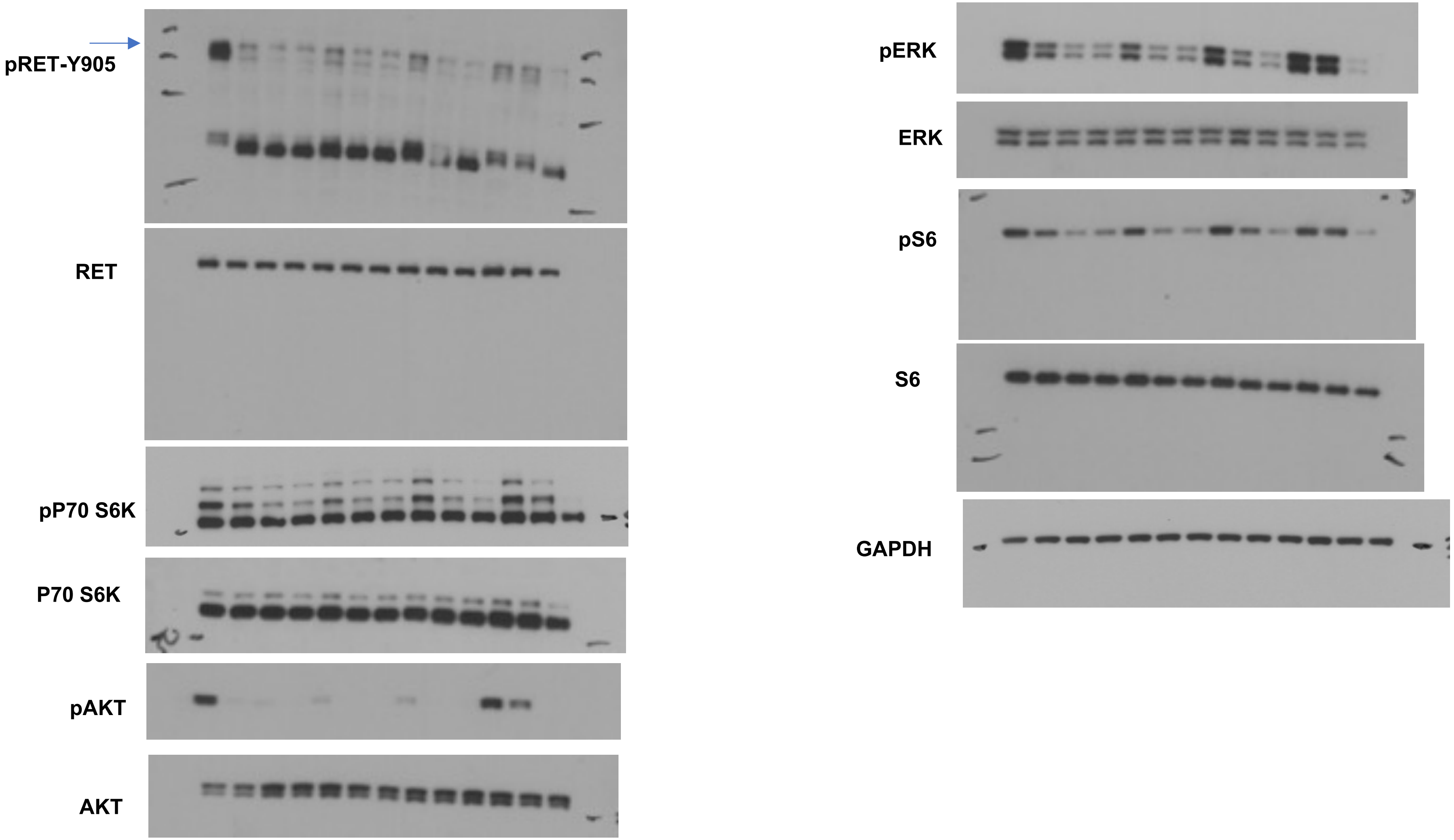

Membranes were cut and each piece probed for the proteins indicated. The blots shown are the entire piece probed and taken directly from the x-ray film without any further modification. Where multiple bands are evident, the arrow indicates the protein of interest. Black check (pen) marks on some blots indicate molecular weight markers placed on film after development.

Source Data Fig. 3a

TT Cells

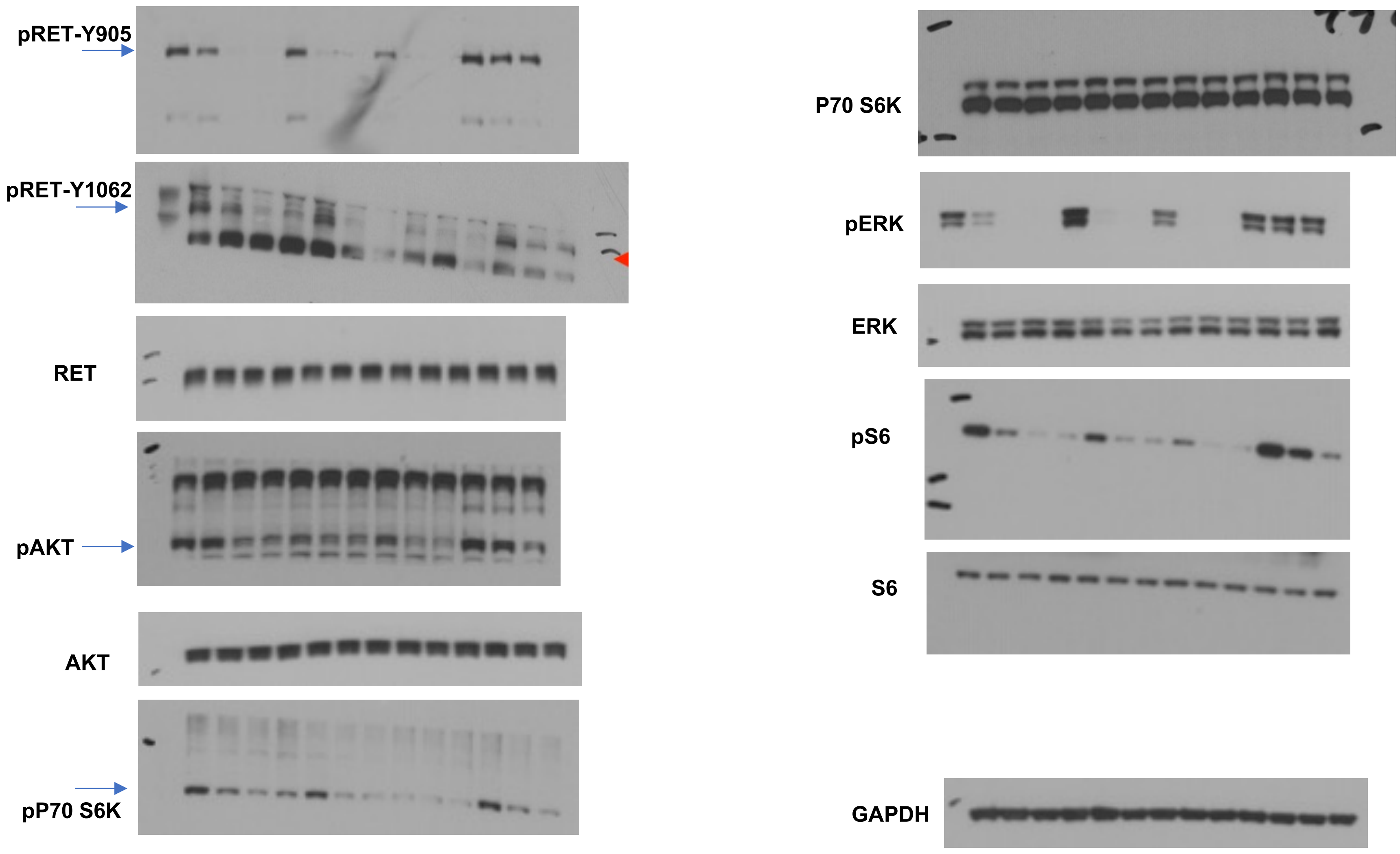

Membranes were cut and each piece probed for the proteins indicated. The blots shown are the entire piece probed and taken directly from the x-ray film without any further modification. Where multiple bands are evident, the arrow indicates the protein of interest. Black check (pen) marks on some blots indicate molecular weight markers placed on film after development.

Source Data Fig. 4a

LUAD-0002AS1

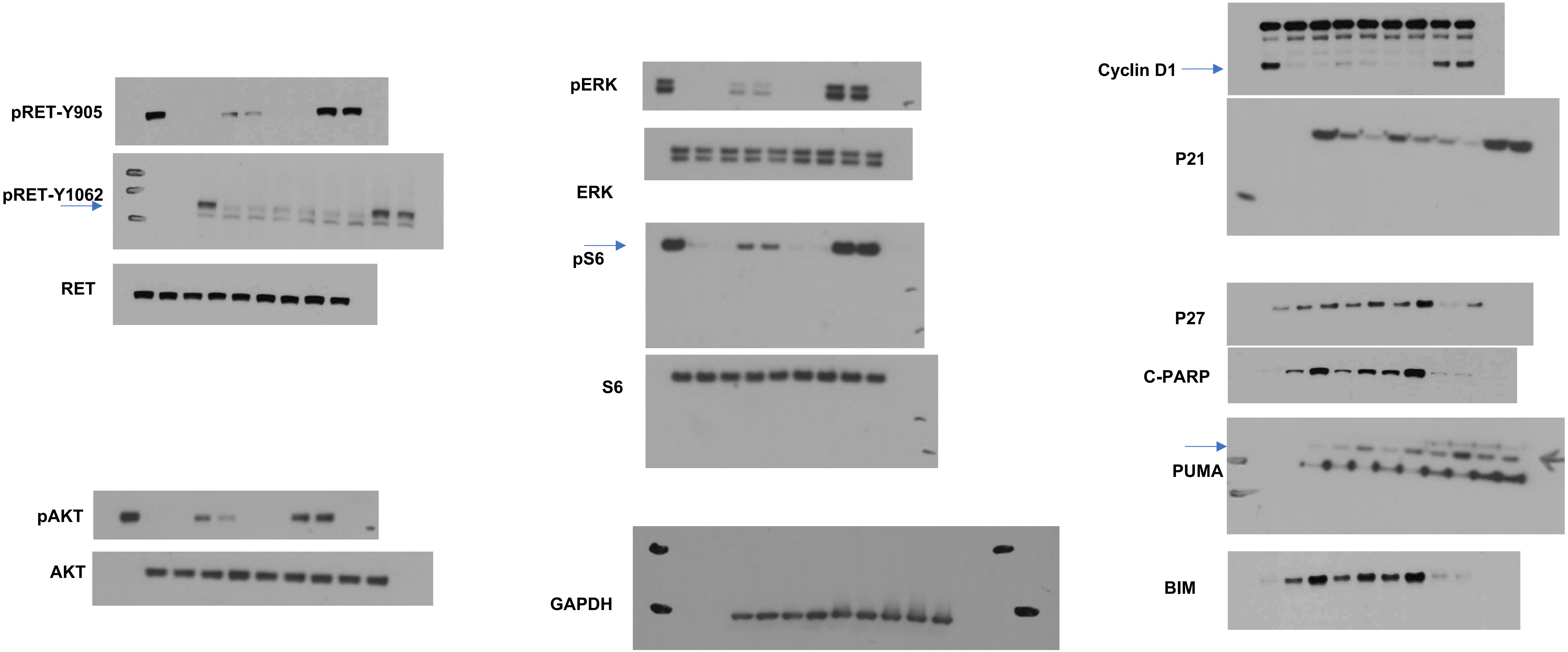

Membranes were cut and each piece probed for the proteins indicated. The blots shown are the entire piece probed and taken directly from the x-ray film without any further modification. Where multiple bands are evident, the arrow indicates the protein of interest. Black check (pen) marks on some blots indicate molecular weight markers placed on film after development.

Source Data Fig. 4a

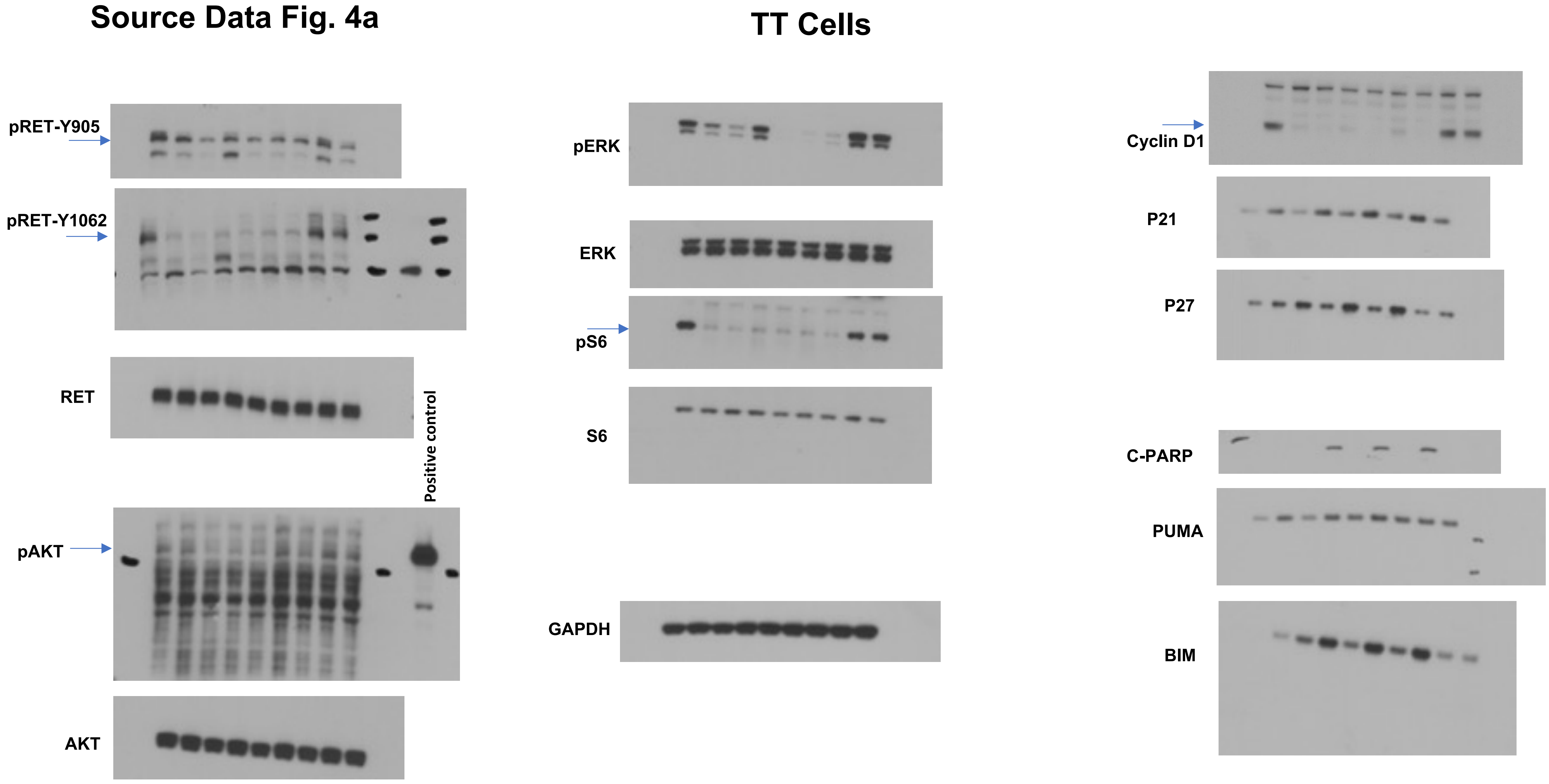

Membranes were cut and each piece probed for the proteins indicated. The blots shown are the entire piece probed and taken directly from the x-ray film without any further modification. Where multiple bands are evident, the arrow indicates the protein of interest. Black check (pen) marks on some blots indicate molecular weight markers placed on film after development.

Source Data Fig. 7b

pRET
(p-KIF5B-RET)

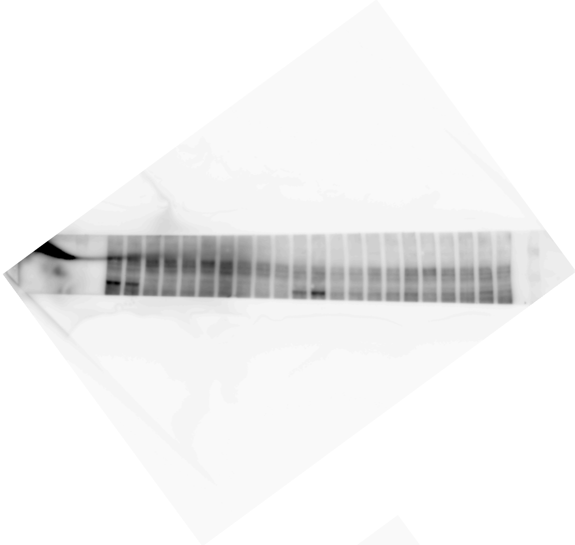

pERK

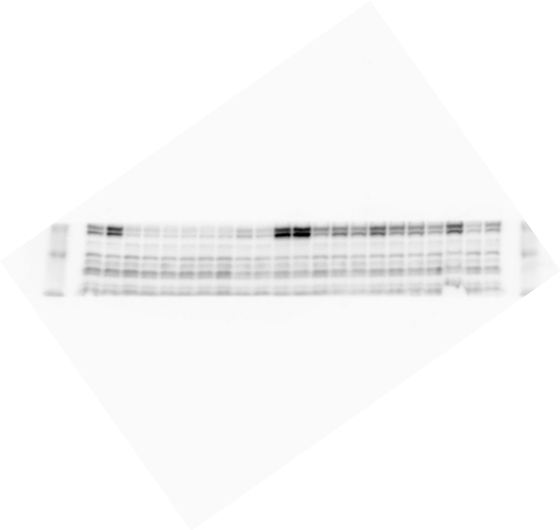

RET
(KIF5B-RET)

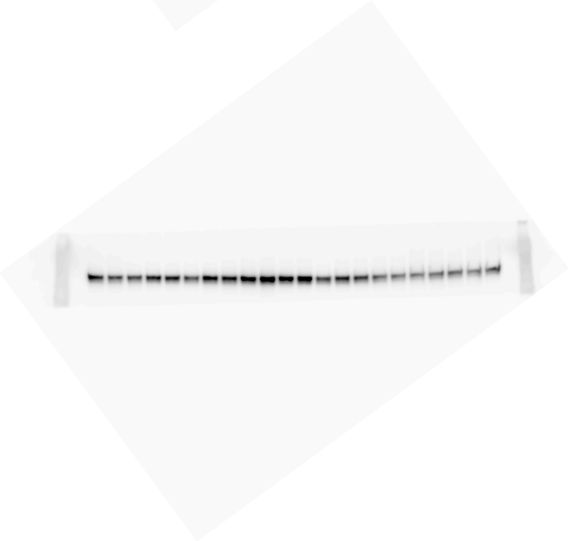

ERK

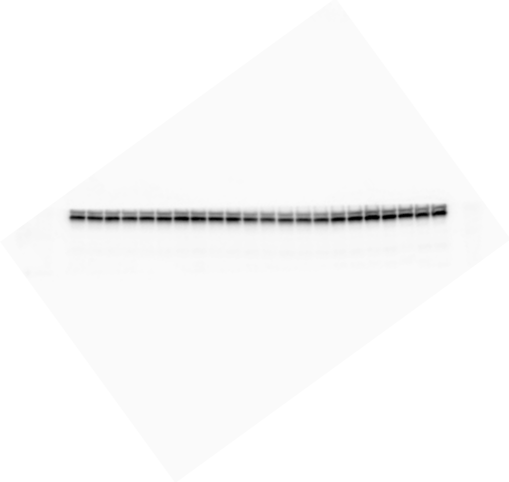

GAPDH

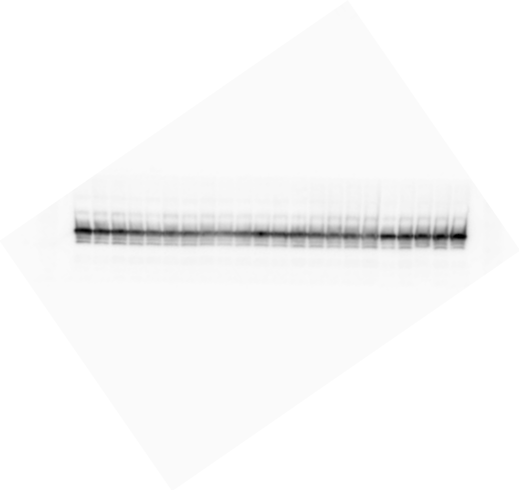

Source Data Fig. 7e

pRET
(p-KIF5B-RET)

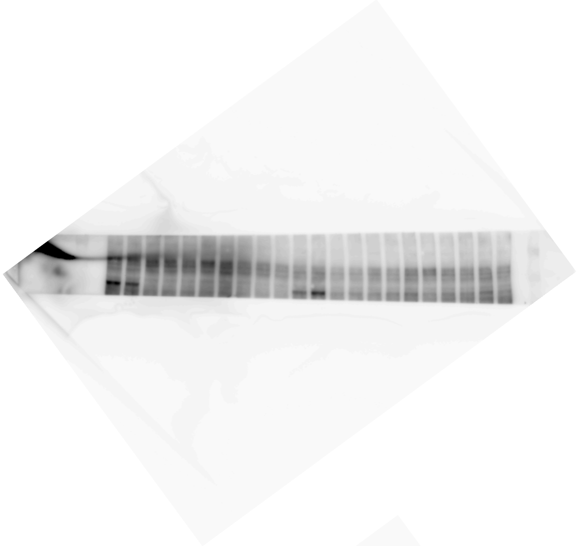

pERK

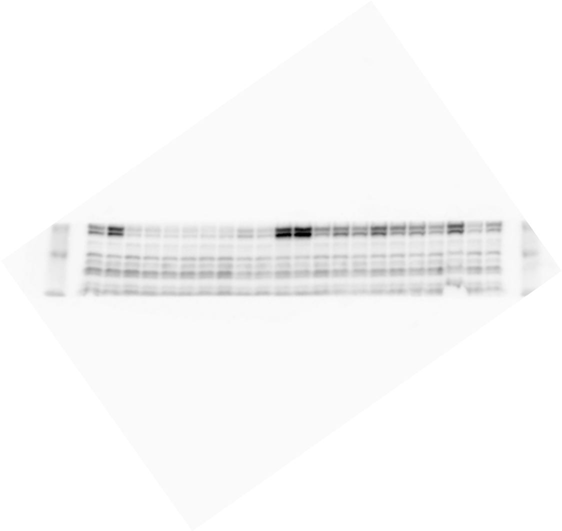

RET
(KIF5B-RET)

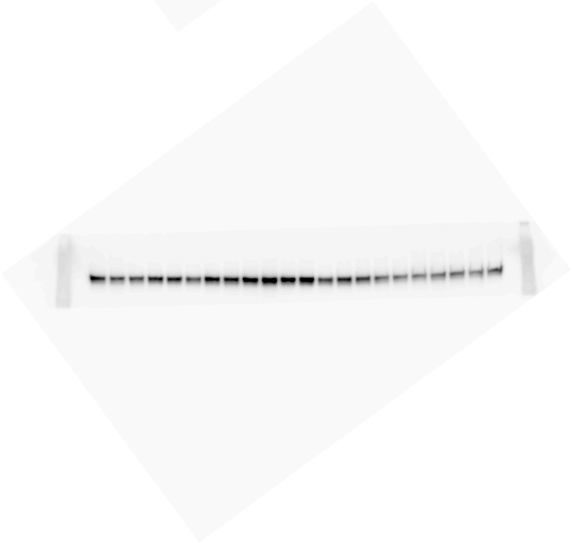

ERK

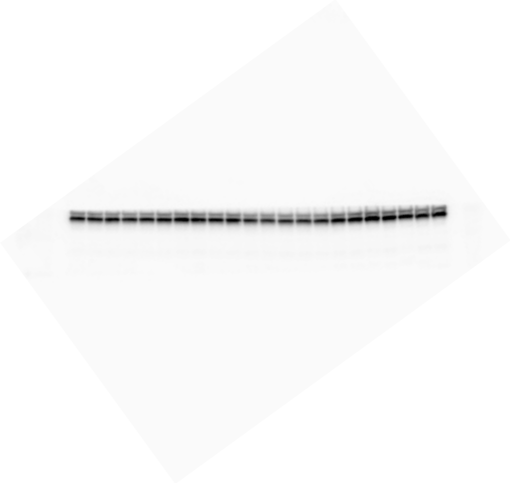

GAPDH

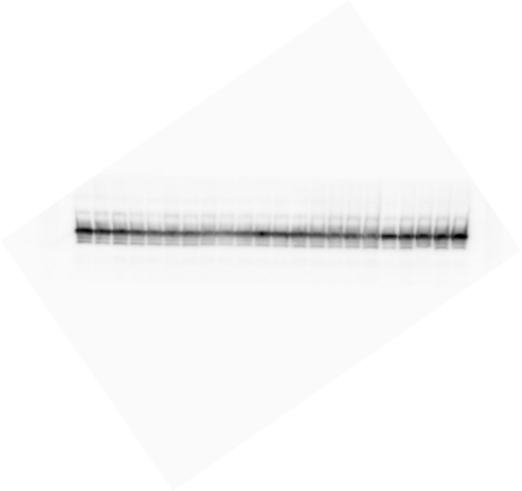

Source Data Extended Data Figure 4a

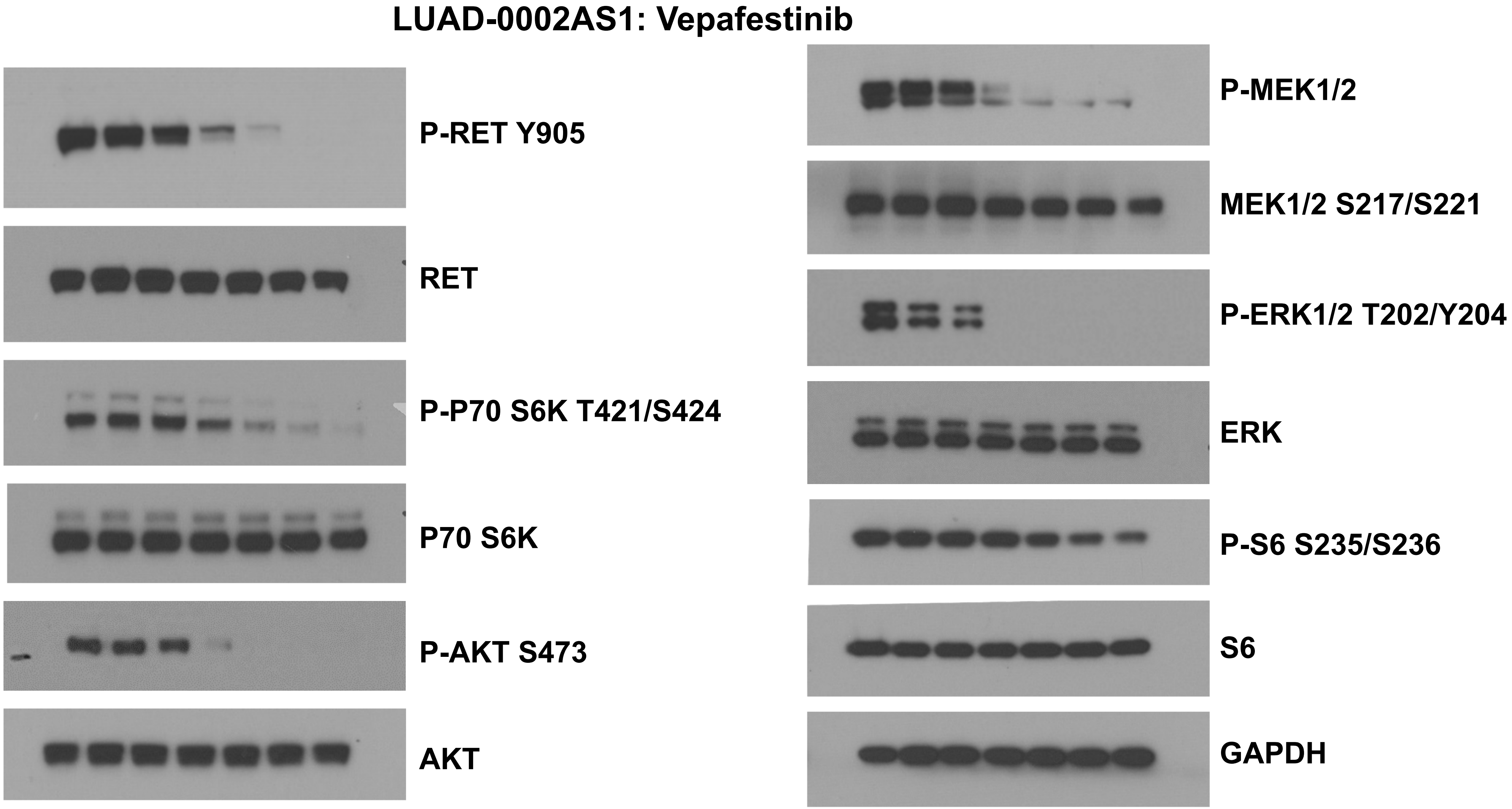

Membranes were cut and each piece probed for the proteins indicated. The blots shown are the entire piece probed and taken directly from the x-ray film without any further modification. Where multiple bands are evident, the arrow indicates the protein of interest. Black check (pen) marks on some blots indicate molecular weight markers placed on film after development.

Source Data Extended Data Figure 4a

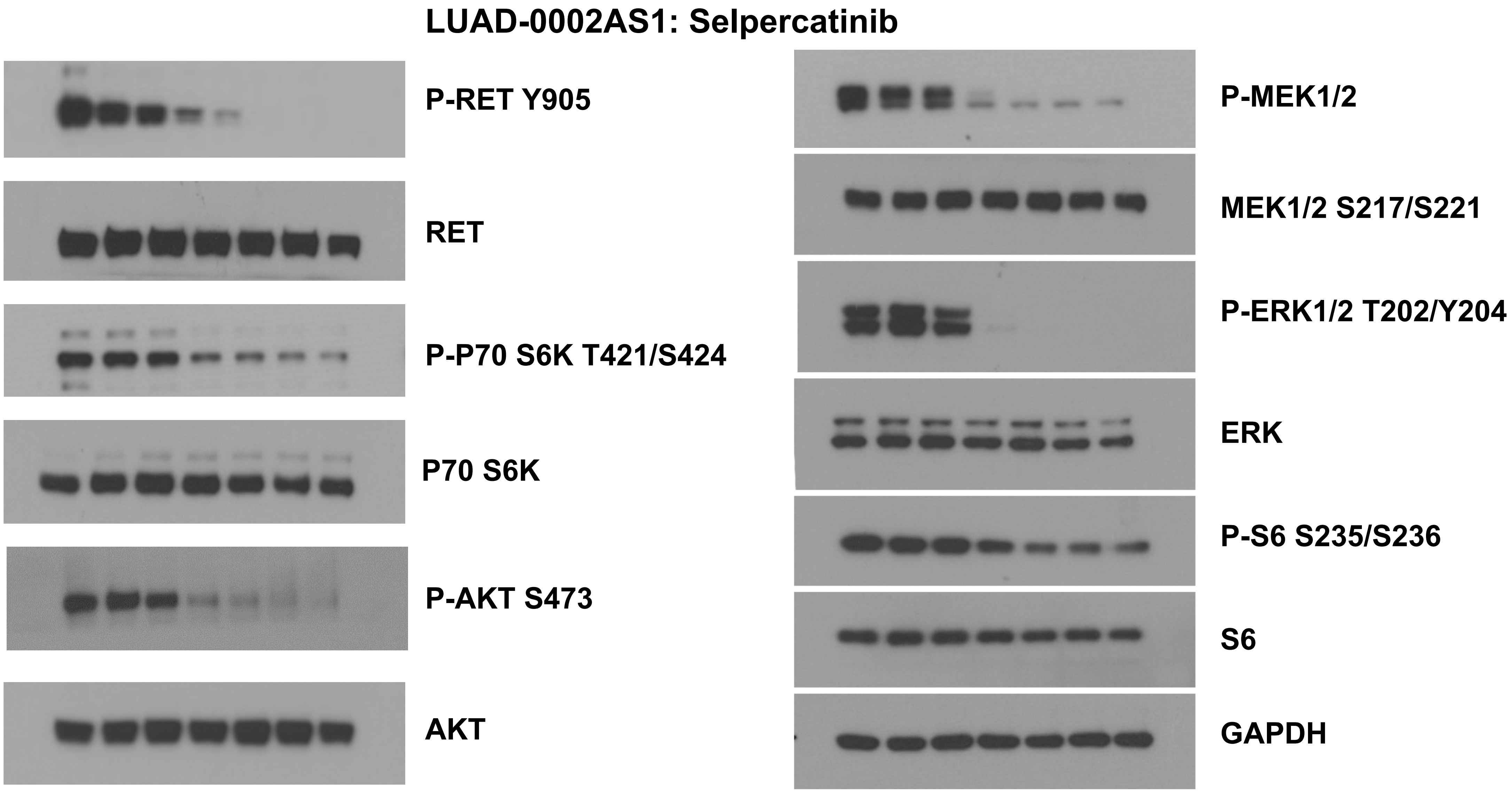

Membranes were cut and each piece probed for the proteins indicated. The blots shown are the entire piece probed and taken directly from the x-ray film without any further modification. Where multiple bands are evident, the arrow indicates the protein of interest. Black check (pen) marks on some blots indicate molecular weight markers placed on film after development.

Source Data Extended Data Figure 4a

LUAD-0002AS1: Pralsetinib

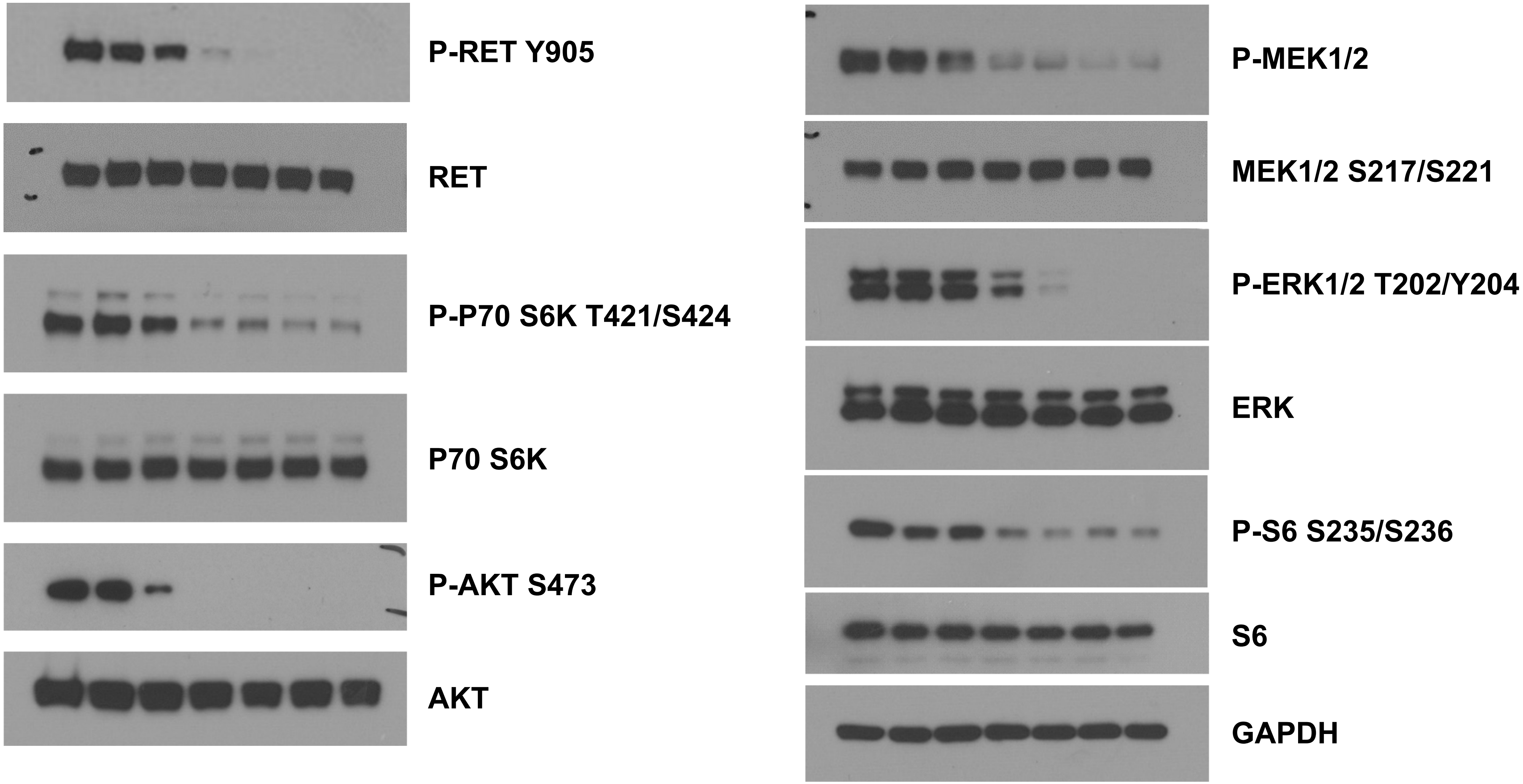

Membranes were cut and each piece probed for the proteins indicated. The blots shown are the entire piece probed and taken directly from the x-ray film without any further modification. Where multiple bands are evident, the arrow indicates the protein of interest. Black check (pen) marks on some blots indicate molecular weight markers placed on film after development.

Source Data Extended Data Figure 4b

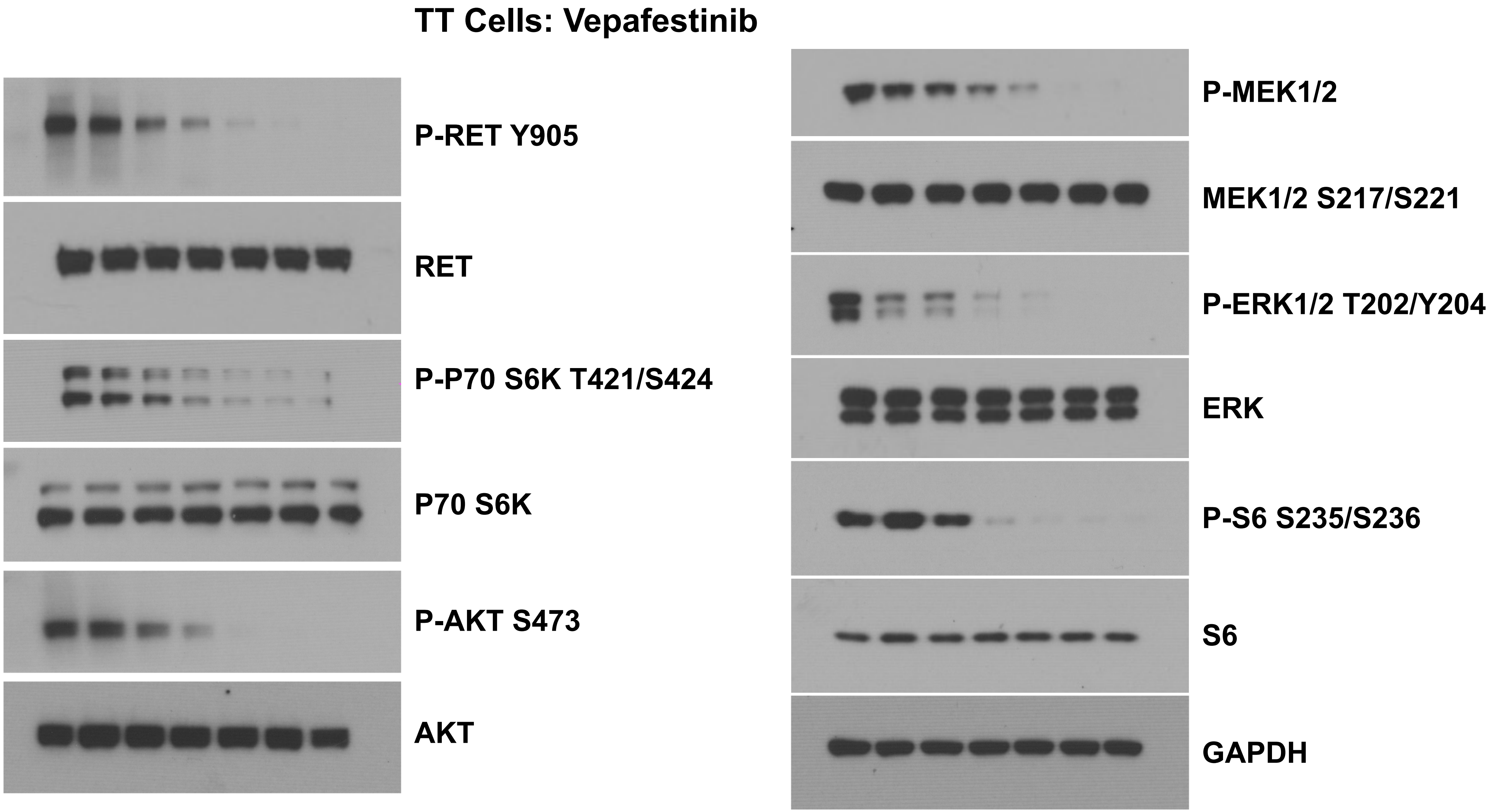

Membranes were cut and each piece probed for the proteins indicated. The blots shown are the entire piece probed and taken directly from the x-ray film without any further modification. Where multiple bands are evident, the arrow indicates the protein of interest. Black check (pen) marks on some blots indicate molecular weight markers placed on film after development.

Source Data Extended Data Figure 4b

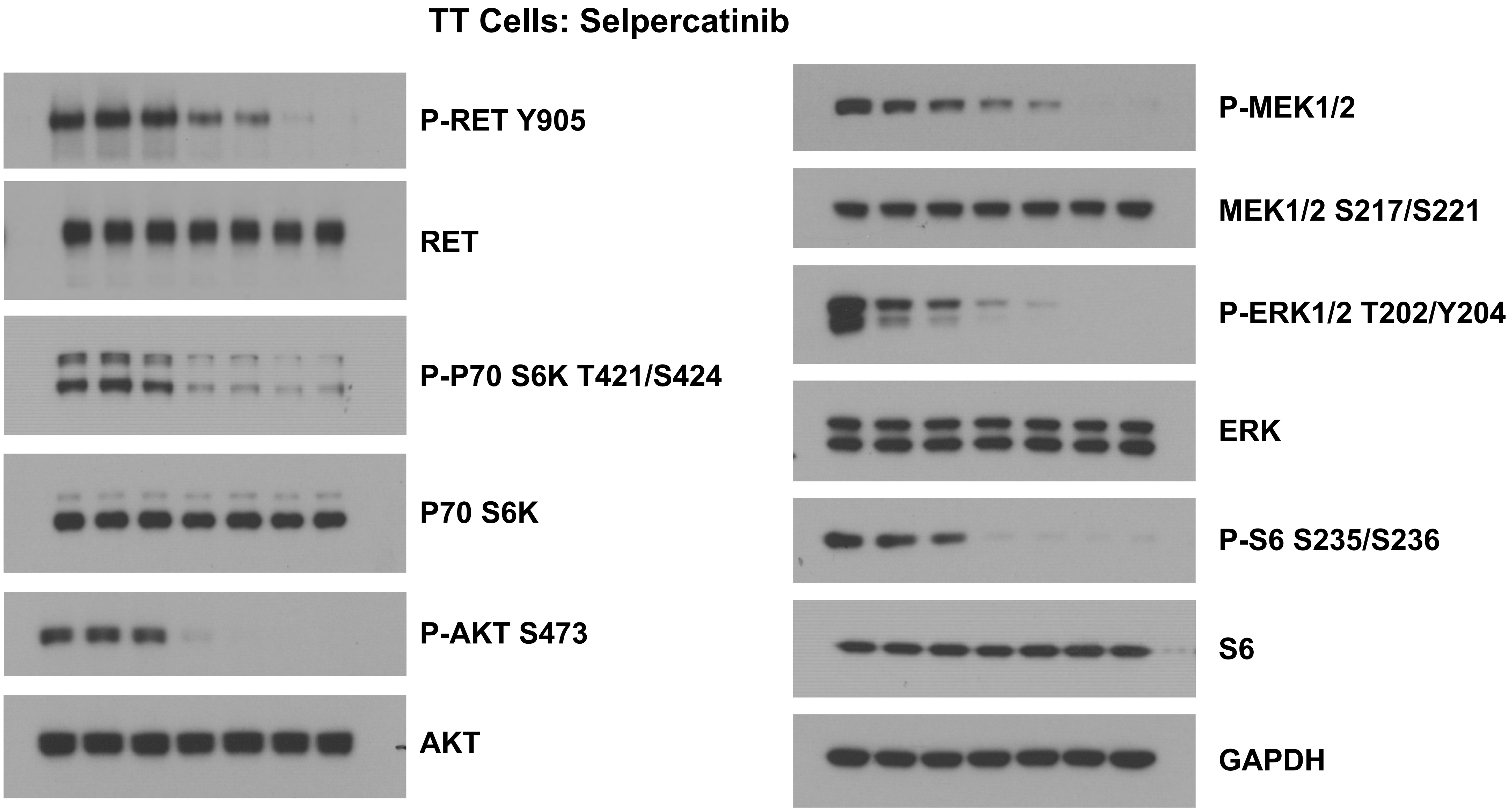

Membranes were cut and each piece probed for the proteins indicated. The blots shown are the entire piece probed and taken directly from the x-ray film without any further modification. Where multiple bands are evident, the arrow indicates the protein of interest. Black check (pen) marks on some blots indicate molecular weight markers placed on film after development.

Source Data Extended Data Figure 4b

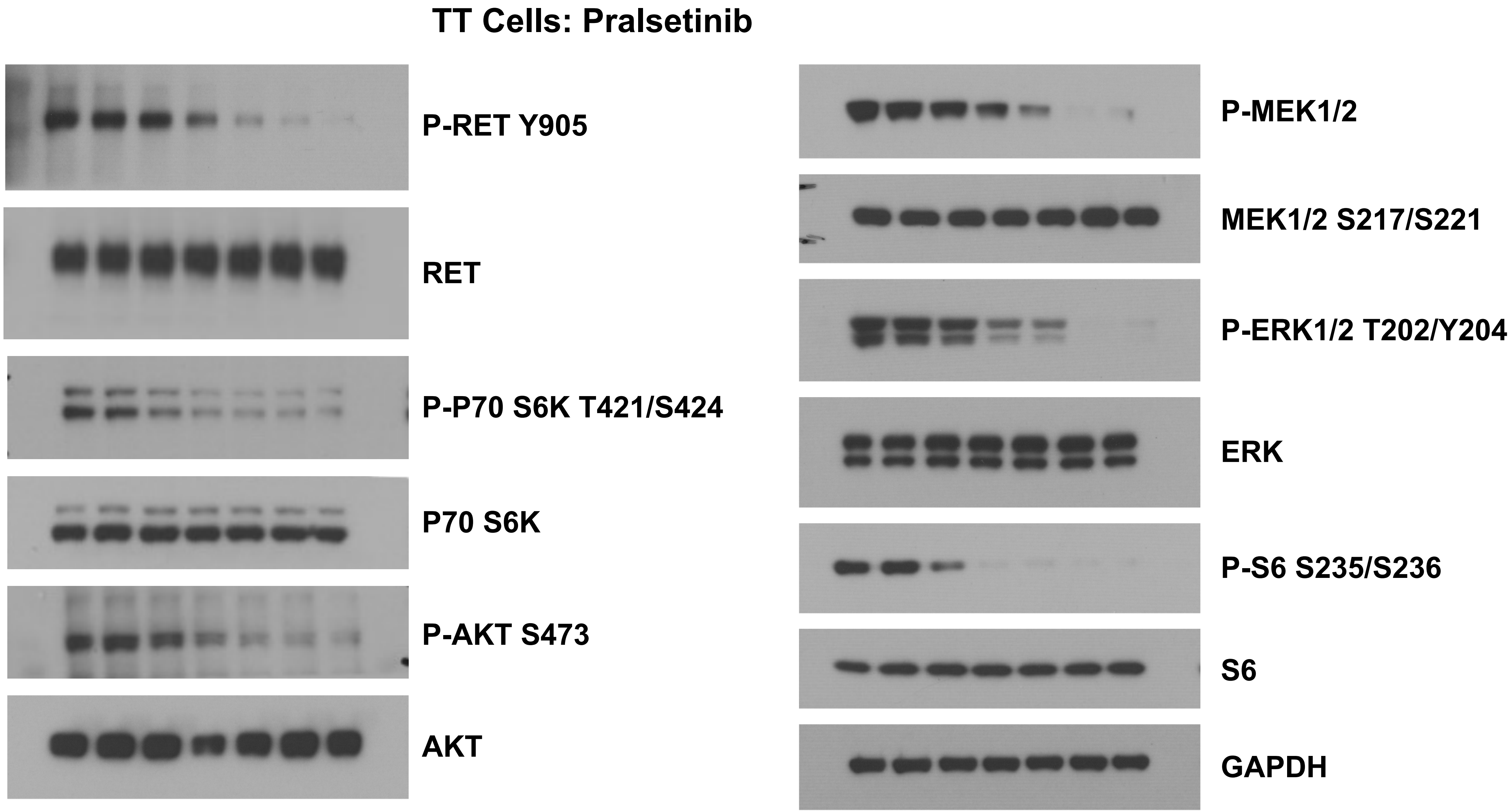

Membranes were cut and each piece probed for the proteins indicated. The blots shown are the entire piece probed and taken directly from the x-ray film without any further modification. Where multiple bands are evident, the arrow indicates the protein of interest. Black check (pen) marks on some blots indicate molecular weight markers placed on film after development.
